# Electronic commensuration of a spin moiré superlattice in a layered magnetic semimetal

**DOI:** 10.1126/sciadv.adu6686

**Published:** 2025-02-05

**Authors:** Takashi Kurumaji, Nisarga Paul, Shiang Fang, Paul M. Neves, Mingu Kang, Jonathan S. White, Taro Nakajima, David Graf, Linda Ye, Mun K. Chan, Takehito Suzuki, Jonathan Denlinger, Chris Jozwiak, Aaron Bostwick, Eli Rotenberg, Yang Zhao, Jeffrey W. Lynn, Efthimios Kaxiras, Riccardo Comin, Liang Fu, Joseph G. Checkelsky

**Affiliations:** ^1^ Department of Physics, Massachusetts Institute of Technology, Cambridge, MA 02139, USA.; ^2^ Department of Physics and Astronomy, Center for Materials Theory, Rutgers University, Piscataway, NJ 08854, USA.; ^3^ Department of Physics, Harvard University, Cambridge, MA 02138, USA.; ^4^ Center for Complex Phase of Materials, Max Planck POSTECH Korea Research Initiative, Pohang 790-884, Republic of Korea.; ^5^ Laboratory for Neutron Scattering and Imaging (LNS), Paul Scherrer Institute (PSI), CH-5232 Villigen, Switzerland.; ^6^ The Institute for Solid State Physics, The University of Tokyo, Kashiwa, Chiba 277-8581, Japan.; ^7^ RIKEN Center for Emergent Matter Science (CEMS), Wako 351-0198, Japan.; ^8^ National High Magnetic Field Laboratory, Tallahassee, FL 32310, USA.; ^9^ National High Magnetic Field Laboratory, LANL, Los Alamos, NM 87545, USA.; ^10^ Advanced Light Source, E. O. Lawrence Berkeley National Laboratory, Berkeley, CA 94720, USA.; ^11^ NIST Center for Neutron Research, National Institute of Standards and Technology, Gaithersburg, MD 20899, USA.; ^12^ Department of Materials Science and Engineering, University of Maryland, College Park, MD 20742, USA.; ^13^ John A. Paulson School of Engineering and Applied Sciences, Harvard University, Cambridge, MA 02138, USA.

## Abstract

Spin moiré superlattices (SMSs) have been proposed as a magnetic analog of crystallographic moiré systems and a source of electron minibands offering vector-field moiré tunability and Berry curvature effects. However, it has proven challenging to realize an SMS in which a large exchange coupling *J* is transmitted between conduction electrons and localized spins. Furthermore, most systems have carrier mean free paths *l*_mfp_ shorter than their spin moiré lattice constant *a*_spin_, inhibiting miniband formation. Here, we discover that the layered magnetic semimetal EuAg_4_Sb_2_ overcomes these challenges by forming an interface with *J* ~ 100 milli–electron volts transferred between a Eu triangular lattice and anionic Ag_2_Sb bilayers hosting a two-dimensional electron band in the ballistic regime (*l*_mfp_ >> *a*_spin_). The system realizes an SMS with *a*_spin_ commensurate with the Fermi momentum, leading to a marked quenching of the transport response from miniband formation. Our findings demonstrate an approach to magnetically engineering moiré superlattices and a potential route to an emergent spin-driven quantum Hall state.

## Teaser

A layered semimetal EuAg_4_Sb_2_ hosts a spin superlattice strongly coupled to quasi-two-dimensional ballistic electrons.

## INTRODUCTION

A heterostructure of two-dimensional (2D) crystals with a relative twist or lattice mismatch produces a moiré superlattice potential. The conduction electrons form mini flat bands enhancing the effects of electronic correlation, resulting in a wealth of correlated electronic phases (*1*, *2*). A distinct approach to the design and construction of moiré flat bands is to embed itinerant electrons in a spin modulation incommensurate with the atomic lattice to impose a superlattice exchange potential. Recent theoretical works have demonstrated that nontrivial spin structures such as skyrmion lattices (SkLs) (*3*–*5*) that form spin moiré superlattices (SMSs) may enable unique modulated electronic behavior (*6*). Similar to conventional moiré systems, the Fermi surface of the conduction electrons is strongly reconstructed into a magnetic Brillouin zone (BZ) defined by the periodicity of the SMS (*5*, *7*). However, compared to crystallographic moiré lattices, it is expected that a magnetically derived moiré system could support relatively straightforward tunability (e.g., with magnetic field) and enable new phenomena with the vector-like nature of the magnetism (compared to a scalar charge modulation) (*8*).

Despite these theoretical predictions, experimental realization of all of the necessary ingredients for SMSs has proven challenging. A first requirement is a modulated magnetic superlattice with a multi-*q* structure. Recent advances have established such textures in a growing number of thin films and bulk crystalline magnets (*9*). However, among the electrically conducting multi-*q* systems, there is a scarcity of electronically clean compounds or Fermi surfaces capable of nesting with the magnetic BZ of the underlying magnetic texture. A further challenge lies in the realization of relatively large exchange coupling constant *J* between conduction electrons and an underlying spin superlattice that can reconstruct the Fermi surface.

## RESULTS

**Fig. 1.**
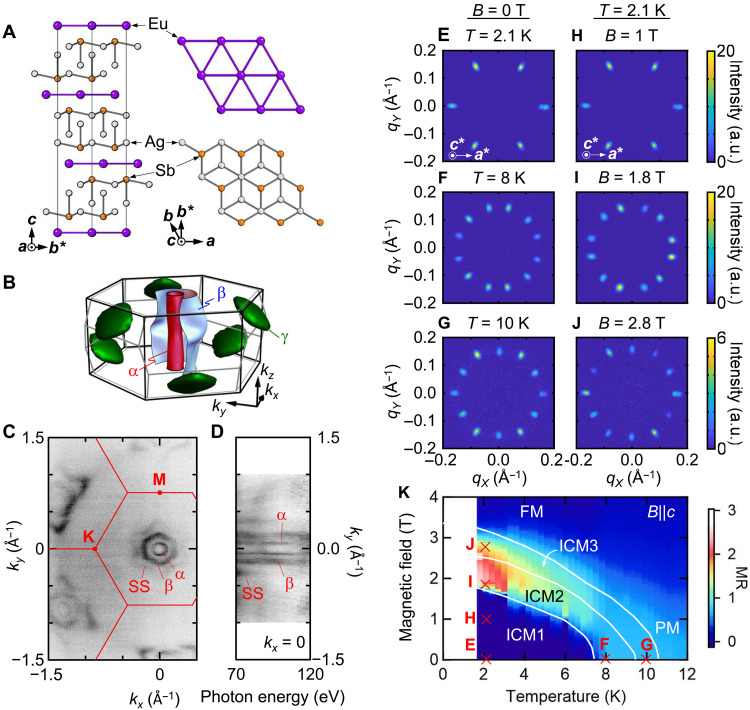
Heterostructure of the 2D electron layer and frustrated magnetic lattice. (**A**) Crystal structure of EuAg_4_Sb_2_. Left: Side view. Right: Top view of the magnetic Eu triangular lattice (top) and conductive SbAg_2_-Ag_2_Sb layer (bottom). (**B**) Fermi surfaces obtained by the DFT calculations. Red, blue, and green sheets are for the α (hole), β (hole), and γ (electron) pockets, respectively. The hexagonal (rhombohedral) BZ is shown by the thick (thin) line. (**C**) Fermi surfaces of EuAg_4_Sb_2_ measured with ARPES. The BZs are marked with the red solid hexagons. (**D**) Photon energy dependence of the Fermi surfaces giving a *k_z_* dispersion in the (0, *k_y_*, *k_z_*) plane. (**E** to **G**) SANS pattern in zero field (E) *T* = 2.1 K for the ICM1 phase, (F) 8 K for ICM2, and (G) 10 K for ICM3. The color scale is the same with those next to (H) to (J), respectively. (**H** to **J**) Corresponding external magnetic field (*B*)–dependent SANS pattern at *T* = 2.1 K for (H) *B* = 1 T for ICM1, (I) 1.8 T for ICM2, and (J) 2.8 T for ICM3 with *B*||*c*. The neutron beam is nearly parallel to the *c* axis, and the intensity is integrated across the rocking scan. a.u., arbitrary units. (**K**) *B*-*T* phase for the external magnetic field *B*||*c* with the color plot of the MR. PM and FM are the paramagnetic and field-induced ferromagnetic state, respectively.

Here, we study the SMS within magnetic layered semimetals, where a magnetic subsystem is alternately stacked with conductive semimetallic layers hosting highly mobile electrons and relatively low Fermi energy *E*_F_ (*10*–*12*), as a route to overcome these challenges. A platform material which connects to these considerations is EuAg_4_Sb_2_, which crystalizes into the rhombohedral CaCu_4_P_2_ type structure (*R*-3*m*, space group 166) (*13*). As shown in Fig. 1A, the crystal structure is the alternate stacking of triangular lattices of magnetic Eu atoms and Ag_2_Sb bilayers of Zintl-Klemm type {Eu^2+^[(Ag_2_Sb)^−^]_2_}. Previous studies reported successive magnetic transitions along with metamagnetism (*14*, *15*), suggesting the presence of magnetic frustration in the Eu triangular lattice layer. The conduction bands are mainly composed of Sb-5s/5p orbitals on the anionic layer (*13*) coordinated with a neighboring Eu layer along the *c* axis.

To shed light on the electronic structure of EuAg_4_Sb_2_, we performed ab initio density functional theory (DFT) calculations of the Fermi surfaces (Fig. 1B). These reproduce the hole pockets observed by the angle-resolved photoemission electron spectroscopy (ARPES) as shown in Fig. 1C for the Fermi surface at 104 eV. We observe intensity for three pockets, α, β, and a surface state (SS) around the Γ point (section S1). The SS spectrum is reproduced by the DFT calculation as well in a slab geometry. The shape of the α pocket is cylindrical with a minimal *k_z_* dispersion, whereas the β pocket shows a stronger warping open along the *k_z_* direction (see the photon energy dependence in Fig. 1D). The 2D nature of the electronic structure can also be inferred from a large anisotropy of single-crystal resistivity. We observed an enhanced out-of-plane to in-plane resistivity ratio, which is highly anisotropic ρ*_zz_*/ρ*_xx_* = 230 at *T* = 1.8 K (section S2 and fig. S5). In high–magnetic field measurements, we observe Shubnikov–de Haas (SdH) oscillations from the α and β pockets open along the out-of-plane (*k_z_*) direction with band parameters consistent with DFT calculations (section S3 and figs. S6 to S8).

We characterized the magnetic modulation of the SMS via single-crystal small-angle neutron scattering (SANS). Three distinct incommensurately modulated phases (ICM1, ICM2, and ICM3) are observed at zero field (see Fig. 1, E to G) and subsequent application of external magnetic field *B*||*c* at *T* = 2.1 K (see Fig. 1, H to J; see also Fig. 1K for the field-temperature phase diagram and details in section S4). The ground (ICM1) state (Fig. 1E) exhibits a pattern with sixfold symmetry around the *c* axis for magnetic modulation vectors equivalent with ***q***_ICM1_ ≈ (0.165, 0, 0.014 Å^−1^) in Cartesian (*q_x_*, *q_y_*, *q_z_*) coordinates, potentially related to the *ab*-cycloid structure previously reported in the structurally similar system EuAg_4_As_2_ (*16*).

**Fig. 2.**
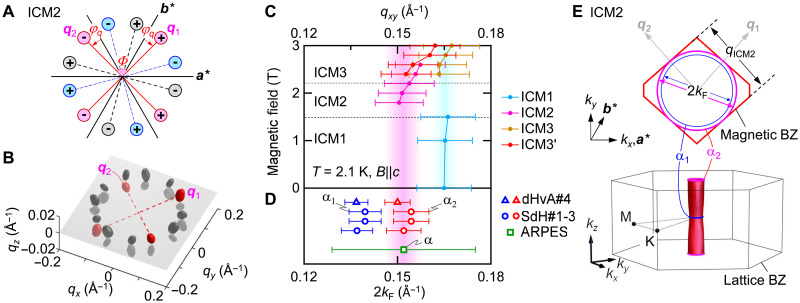
Matching between the Fermi surface and magnetic modulation period of EuAg_4_Sb_2_. (**A**) Schematic *q*-vector configuration for the ICM2 state. Rhombic double-*q* domains are color coded (red, blue, and black). Plus and minus signs in each circle denote that of the *q_z_* coordinate. φ*_q_* is the azimuthal rotation of *q* vectors away from high-symmetry directions. Φ is the mutual angle of rhombic double-*q* vectors, ***q***_1_ and ***q***_2_. (**B**) 3D configuration of the *q* vectors of the ICM2 state in reciprocal space reproduced from SANS experiments (see section S5). The *q*-vector positions, ***q***_1_ and ***q***_2_, belonging to a double-*q* domain are highlighted in red. Note the *q_z_* scale is expanded for visibility. (**C**) Magnetic field–dependent in-plane magnetic modulation *q_xy_* = (*q_x_*^2^ + *q_y_*^2^)^1/2^ in units of Å^−1^ for each magnetic state at *T* = 2.1 K in *B*||*c*. Bar with caps is width of the peak comparable to the instrumental resolution (~0.008 Å^−1^). (**D**) Diameter of the Fermi surface (2*k*_F_) for the α pocket estimated with ARPES and for the α_1_ and α_2_ estimated from SdH and dHvA oscillations in each sample (#1-#4) with *B*||*c* (see table S1). The open symbol is the central value, and the bar with caps is the SD. (**E**) Top: Schematic α-Fermi surface in the lattice BZ. Bottom: Geometric relationship (viewed from the *k_z_* axis) for the extremal cross sections of the α pocket (pink and blue circles) and the magnetic BZ for an SMS (double-*q*) domain (red polygons) at *T* = 2.1 K and *B* = 2 T for the *c* axis, where φ*_q_* ≈ 12° and Φ ≈ 84°.

From the ground state, the magnetic structure evolves with increasing *T* or *B* to a multiple-spot pattern in ICM2 represented by ***q***_ICM2_ ≈ (0.146, 0.031, ±0.007 Å^−1^) (see, e.g., Fig. 1, F and I) with a corresponding magnetic modulation wavelength λ_ICM2_ = 4.6 nm. Tilted field experiments demonstrate the ICM2 magnetic order to be multi-*q* in nature (see section S5 and figs. S13 and S14). The pattern can be described by the superposition of domains of equivalent double-*q* structures, each composed of *q* vectors, ***q***_1_ and ***q***_2_, with a mutual angle Φ ≈ 84° at *T* = 2.1 K and *B* = 1.8 T for *B*||*c* (Fig. 2A illustrates this arising from an azimuthal *q*-rotation φ*_q_* ≈ 12°, leading to the near right angle configuration, i.e., Φ = 60° + 2φ*_q_*). This state is followed by an additional ICM3 phase characterized by a 12-spot pattern, which can be decomposed into two inequivalent six-spot patterns of larger (*q*_ICM3_) and smaller (*q*_ICM3′_) |*q*|. The former and the latter are proximate to *q*_ICM1_ and *q*_ICM2_ in length, respectively. The double-*q* spin structure in the ICM2 phase breaks the symmetry of the original atomic lattice. Whereas contrary to the tendency observed in Gd-based centrosymmetric materials (*17*–*19*), such a lowered symmetry SMS has been reported in Eu-based magnets, e.g., the rhombic SkL in EuAl_4_ (*20*) and distorted triangular SkL in a tetragonal EuNiGe_3_ (*21*).

Although the *q* vectors of the ICM2 state are nearly confined in the layer plane, they have nonzero *q_z_* (see Fig. 2B), which enriches the magnetic texture. The four peaks highlighted in red belong to a single double-*q* domain of the ICM2 state (see section S5). Because of the small *q_z_* component, the plane spanned by the two *q* vectors is tilted from the a^*^b^*^ plane, resulting in a horizontal shift of spin texture at each Eu triangular lattice layer. The length scale of the out-of-plane modulation (λ_ICM2*z*_ ~ 90 nm at *T* = 2.1 K and *B* = 1.8 T) is much longer than the in-plane modulation (λ_ICM2_ ~ 4.6 nm), consistent with the quasi-2D nature of the Fermi surface (see also sections S5 and S10). In the following, we focus on the in-plane interaction of electronic and magnetic structure and impact of the electronic commensuration on the transport response.

In Fig. 2 (C and D), we compare the low-temperature, magnetic field–dependent in-plane magnetic modulation (*q_xy_*) with the α pocket Fermi surface diameter (2*k*_F_) estimated from ARPES, SdH, and dHvA measurements. Quantum oscillation measurements observed two branches α_1_ and α_2_ associated with the *k_z_* warping of a quasi-2D pocket (figs. S6 and S7). Upon increasing magnetic field, the relatively large *q*_ICM1_ evolves to *q*_ICM2_ in the SMS phase, with the latter agreeing well with 2*k*_F_ estimated from these methods. This commensuration relation between *q*_ICM2_ and 2*k*_F_ for the cylindrical α pocket (viz. *q*_ICM2_ = 2*k*_Fα_) suggests a strong coupling between the magnetic and electronic degrees of freedom and may offer insight into the origins of the SMS itself (*5*, *7*). As the field increases, *q*_ICM2_ gradually evolves [a similar phenomenology has been seen in other centrosymmetric rare earth systems (*17*, *19*)] and weakens the commensuration. At higher fields, the *q* vectors for ICM3 are more poorly matched and evolve away from 2*k*_F_ upon approaching the field-induced ferromagnetic phase. Figure 2E illustrates a schematic relationship between the Fermi surfaces and the magnetic BZ defined with two ***q*** vectors for the double-*q* structure of the ICM2 state. In an electronically clean system with much longer electron mean free path than the period of spin modulation (*l*_mfp_ >> *a*_spin_), electrons at the α pocket are expected to respond to the periodic potential produced by the SMS for the ICM2 state, resulting in a reconstructed electronic structure.

**Fig. 3.**
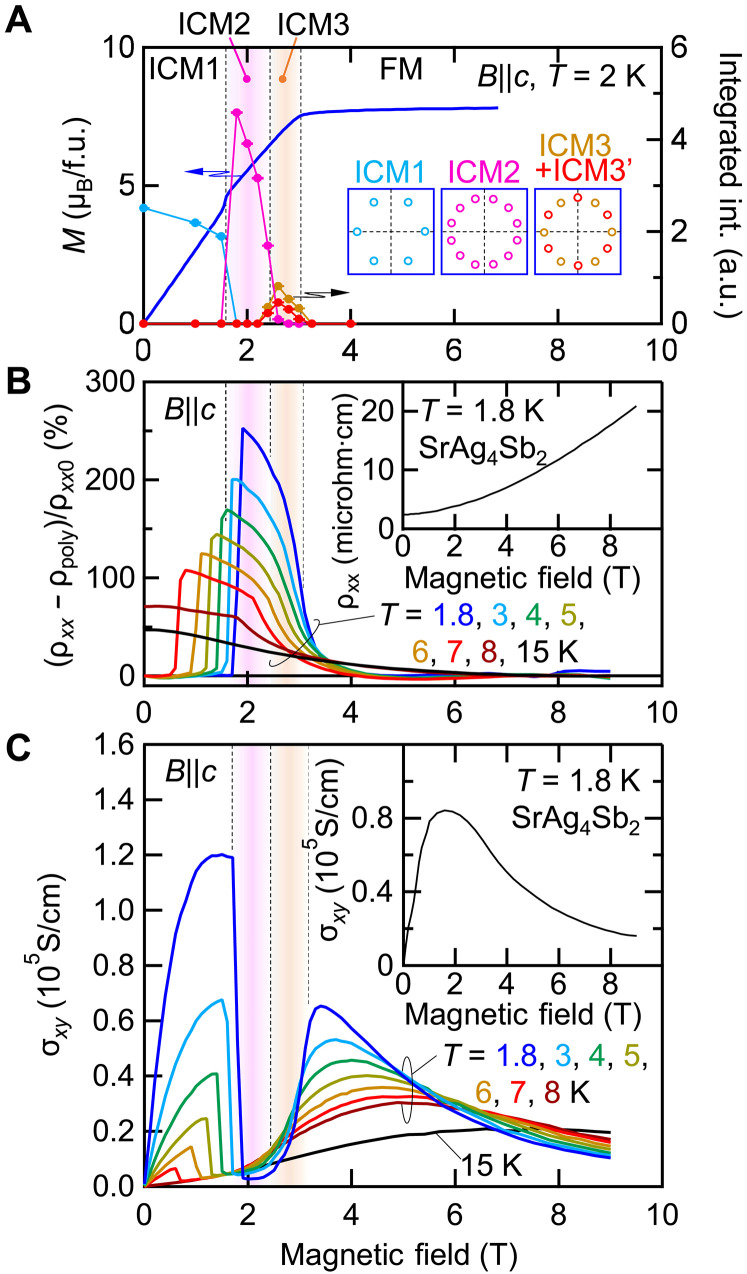
Magnetic field–induced transitions and quenched transport response. (**A** to **C**) Magnetic field dependence of (A) magnetization (*M*) per formula unit (f.u.) and the scattering intensity for each phase as depicted in the inset, (B) MR, and (C) Hall conductivity (σ*_xy_*) for *B*||*c*. For MR, (ρ*_xx_* − ρ_poly_)/ρ_*xx*0_ is obtained by subtracting a smooth polynomial background (ρ_poly_) from ρ*_xx_* and normalized by ρ_*xx*0_, the value at zero field. Inset in (B) and (C) is the field dependence of ρ*_xx_* and σ*_xy_* for nonmagnetic isomorph SrAg_4_Sb_2_ at *T* = 1.8 K.

A comparison of this evolution in magnetic structure to that of magnetotransport reveals an acute coupling between the two. As shown in Fig. 3A, for *B*||*c*, the integrated intensity for each incommensurate phase tracks changes in *M*(*H*) before entering the field-induced ferromagnetic phase (see fig. S9 for details). Schematic *q* positions in reciprocal space for each magnetic phase are depicted in insets in Fig. 3A. The magnetoresistivity (MR) (Fig. 3B) and Hall conductivity (σ*_xy_*; Fig. 3C) in the same parameter range (see section S6 for analysis) show prominent changes upon entering the ICM2 region, with the former undergoing a sharp enhancement with a peak at the optimal 2*k*_F_ = *q*_ICM2_ and the latter being quenched nearly to zero. As can be seen by comparing with the nonmagnetic analog SrAg_4_Sb_2_ [see the insets in Fig. 3, B and C, and section S7; a similar result has been reported in Green *et al*. (*22*)], the high mobility transport response is insensitive to the magnetism in ICM1, begins to recover in ICM3, and reemerges in the FM state. Given their similarity, the large Hall conductivity anomaly exceeding 10^5^ S/m is difficult to reconcile with changes in spin-scattering across these phases. We measured the anomalous magnetotransport properties at various temperatures and fields (fig. S18) and confirmed that this behavior persists across the magnetic phase diagram (see the color plot of magnetoresistance; Fig. 1K), indicating instead a superzone gap opening of the electron bands renormalized in the SMS states. We note that the magnitude of MR (~250%) is remarkably large compared with the other multi-*q* compounds (*17*, *23*–*25*), signifying the impact of the electronic commensuration with the SMS state.

**Fig. 4.**
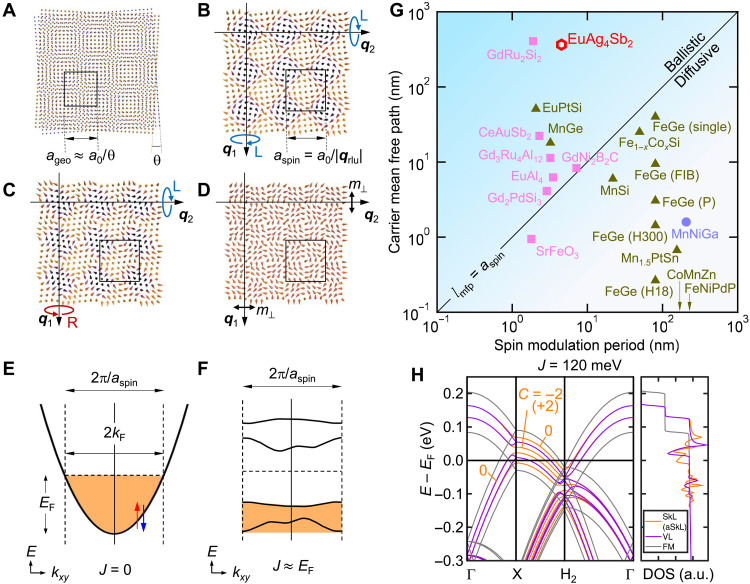
Modeling the reconstructed electronic band in SMSs. (**A** to **D**) Schematic illustration of (A) moiré superlattice due to a geometrical twist angle θ and [(B) to (D)] SMSs produced by the double-*q* configuration of a long wavelength spin modulation with ***q****_i_* (*i* = 1, 2): (B) square SkL, (C) square aSkL, (D) VL. *a*_0_, *a*_geo_, and *a*_spin_ are the original, crystal moiré, and spin moiré lattice constant, respectively. The black line denotes the magnetic unit cell. Arrows denoted by L (R) and *m*_⊥_ represent a left-handed (right-handed) spin helix and an in-plane moment, respectively, for each *q* modulation to compose the SMS. (**E** and **F**) Schematic illustration of (E) the electron band in the absence of magnetic coupling (*J* = 0) and (F) the reconstructed bands via the exchange field resonant to Fermi energy (*J* ≈ *E*_F_) from the spin moiré lattice characterized by the magnetic BZ of the size 2π/*a*_spin_. See Eq. 1 for the definition of *J*. (**G**) Logarithmic plot of the carrier mean free path *l*_mfp_ versus spin modulation period *a*_spin_ (see section S9 for their definitions) for a variety of multiple-*q*–hosting materials including the present material (open hexagon). Closed triangle: noncentrosymmetric magnets; closed square: centrosymmetric magnets; closed circle: a biskyrmion material. The region for *l*_mfp_ > *a*_spin_ (< *a*_spin_) corresponds to the ballistic (diffusive) carrier transport regime. Shown are Gd_2_PdSi_3_ (*17*), Gd_3_Ru_4_Al_12_ (*18*), GdRu_2_Si_2_ (*19*), MnSi (*27*, *28*), Fe_1−*x*_Co*_x_*Si (*29*, *30*), FeGe (single) (*31*, *32*), FeGe (P): thin film (82 nm) (*33*), FeGe (FIB) (*34*), FeGe (H300, H18): thin films, 300 or 18 nm (*35*), MnGe (*31*), EuPtSi (*23*), CoMnZn alloys (*36*), Mn_1.5_PtSn alloys (*37*, *38*), FeNiPdP alloys (*39*), MnNiGa (*40*), GdNi_2_B_2_C (*41*, *42*), EuAl_4_ (*20*), CeAuSb_2_ (*24*, *43*), and SrFeO_3_ (*44*). (**H**) Calculated electronic band structure in the magnetic BZ on the left and the density of states (DOS) in arbitrary units on the right. Orange, purple, and gray lines are calculated in the presence of SkL (aSkL), VL, and uniform ferromagnetic background, respectively, with the exchange interaction *J* = 120 meV. The Chern numbers are labeled for the first several bands.

We hypothesize that the remarkable interplay between the magnetic and electronic properties of the present system can be understood within the common framework of geometrical moiré superlattices and SMS as incommensurate potentials. A bilayer of 2D crystals with a small twist (θ) produces a moiré superlattice with a periodicity *a*_geo_ ≈ *a*_0_/θ incommensurate to the underlying lattice constant *a*_0_ (Fig. 4A). By comparison, the superposition of multiple single-*q* spin modulations forms a variety of SMSs depending on the moment modulation (*3*–*6*, *8*). Under finite magnetic field, this includes a rhombic SkL, composed of the superposition of two helices with identical handedness and finite homogeneous magnetization (Fig. 4B): the associated anti-SkL (aSkL; Fig. 4C), and the double-*q* vortex lattice (VL; Fig. 4D) (see section S5). Such multi-*q* modulations impart a periodic exchange potential on the conduction electrons at *a*_spin_ = *a*_0_/|***q***_rlu_| (where |***q***_rlu_| is the length of one of the magnetic *q* vectors in reciprocal lattice units). This periodic potential reconstructs the electron band yielding a new (magnetic) BZ of width 2π/*a*_spin_. In the case of a large SMS exchange potential *J* on the order of the Fermi level *E*_F_, a conventional band (see Fig. 4E and Eq. 1 below) is converted to a set of folded bands (see Fig. 4F) with reduced dispersion separated across *E*_F_. DFT calculations here yield *J*/*E*_F_ ≈ 0.93, consistent with this energetic regime (this estimate is consistent with that determined from analysis of magnetic scattering, confirming the physical relevance of *J* ~ *E*_F_; see section S8).

In order for the SMS to reconstruct the electronic band structure, a further requirement is that the carrier mean free path (*l*_mfp_) sufficiently exceeds the magnetic superlattice constant (*a*_spin_), viewed naturally as a momentum-space reconstruction (*26*). Figure 4G shows the comparison between the *l*_mfp_ and *a*_spin_ for various spin texture–hosting materials (see section S9) (*17*–*20*, *23*, *24*, *27*–*44*). Several 3d transition metal–based chiral magnets show relatively lower mobility compared with rare earth intermetallics. Carrier transport in EuAg_4_Sb_2_ is in the ballistic regime (*l*_mfp_ ≈ 250 nm > λ_ICM2_ = *a*_spin_ ≈ 4.6 nm); analysis of the SdH oscillations (fig. S8) shows that the average quantum mean free path (*l*_q_) of the α pocket among various EuAg_4_Sb_2_ samples is estimated to be *l*_q_ = 48 nm (table S1), which is more than 10 times the SMS modulation wavelength and among the cleanest ever reported.

To examine the emergent SMS electronic structure, we construct an effective model simulating the electronic miniband in the magnetic BZ for a 2D system. We start with the double-exchange model

$$H=-t\sum_{<r\;r\prime >}\left(c_{r\sigma }^{†}c_{r\prime \sigma }+h.c.\right)-\sum_{r}Jc_{r\alpha }^{†}\left({\vec{s}}_{r}\cdot 1/2{\vec{\sigma }}_{\alpha \beta }\right)c_{r\beta }+\mathrm{Bg}s_{r}^{z}$$ (1)

where *t* = (3m^*^a_0_^2^)^−1^ is the nearest-neighbor hopping on the triangular lattice with lattice constant *a*_0_, which reproduces the dispersion of the α pocket and ${\vec{s}}_{r}$ is the vector field describing a spin moiré texture of the Eu local spins with lattice constant *a*_spin_ (see also section S10). We also include a Zeeman field *B*. The Fermi wave number *k*_Fα_ and exchange coupling *J*/*E*_F_ ≈ 0.93 are based on the DFT calculations.

As shown in Fig. 4H, we find that the SkL, aSkL, and VL all fold bands in the moiré BZ and are thus capable of strongly renormalizing the transport response. Because of their *S*^2^ winding density, the SkL and aSkL further produce folded Chern bands (*3*, *45*). For each texture, we find folded bands confirmed by the peak of the density of states. This implies an effective mass renormalization capable of quenching of high mobility bulk transport. This model also highlights the importance of the matching between *q*_ICM2_ and 2*k*_F_ as it guarantees a near integer filling of the magnetic BZ and suppressed band dispersion transport response (see section S10).

## DISCUSSION

Among the key factors in realizing the present SMS response, we highlight the significance of the “resonant condition” *J* ≈ *E*_F_ (*46*, *47*) along with a large mean free path. When the Ruderman-Kittel-Kasuya-Yosida (RKKY) mechanism is the driving force for magnetic ordering, the uniform ferromagnetic state is unstable to a twisted texture below a critical *J*/*E*_F_ ≈ 1.8, a universal ratio at low densities (section S10). Therefore, *J* > 2*E*_F_ favors a ferromagnetic state whereas *J* << *E*_F_ favors a spiral phase but does not strongly alter the band structure (i.e., nearly free electron behavior is expected). Meanwhile, *J* ≈ *E*_F_, the regime of EuAg_4_Sb_2_, features a noncollinear phase with largely reconstructed minibands and a large overall effect of ~100 meV (fig. S25), which can effectively reduce the dispersion across the magnetic BZ.

An ultimate goal of the combination of spin textures and 2D electron sheets is a designer renormalization of the electronic structure. The present system provides important design principles for this, in particular the matching of exchange and Fermi energies and long electronic mean free paths. Furthermore, in contrast to 3d element–based materials, where electronic structure and magnetic properties are derived from the same atomic orbitals, the present lanthanide-based systems are composed of rare earth magnetic layers spatially separated from semimetal layers with low carrier density. Combining such functional components in a single material facilitates an augmented material design space, here enabling high electron mobility and exfoliatability (*11*, *48*). In the pursuit of magnetic texture–driven quantum Hall effect (*3*, *4*), crucial next steps would be to evaluate and control the detailed spin texture in this family of compounds, overcome the large negative bandgap, and obtain a truly 2D form via exfoliation or thin film synthesis (the latter enabling the use of strain and electric/displacement fields). This system provides design principles for realizing emergent magnetic textures in centrosymmetric materials that are strongly coupled to exotic electronic systems.

## METHODS

Single crystals of EuAg_4_Sb_2_ and SrAg_4_Sb_2_ were grown via a self-flux method. The starting materials Eu/Sr, Ag, and Sb were mixed in the molar ratio Eu/Sr:Ag:Sb = 1:24:12. They were loaded into a 2-ml alumina crucible and sealed in an evacuated quartz tube. The growth ampoule was heated to 1100°C and slowly cooled to 650°C at a rate of 1.5°C/hour. The single crystals were separated by decanting the flux in a centrifuge. The typical size of the crystals is 2 by 2 by 0.5 mm^3^ with the wide *ab* planes. The single phase nature of the crystals was checked by powder x-ray diffraction, and the orientation of single crystals was checked by a single-crystal x-ray diffractometer.

Electrical transport measurements were performed by a conventional five-probe method with a typical ac excitation current of 1 mA at typical frequency near 15 Hz. The transport response in low temperatures and a magnetic field was measured using a commercial superconducting magnet and cryostat. The obtained longitudinal and transverse signals were field symmetrized and antisymmetrized to correct for a contact misalignment, respectively. Magnetization measurements were performed using a commercial SQUID (superconducting quantum interference device) magnetometer.

SANS experiments were performed using the SANS-I beamline at the Paul Scherrer Institute, Switzerland. Magnetic SANS patterns were obtained from a 44-mg single crystal of size 6 by 6 by 0.2 mm^3^. The (001) plane was the widest and aligned perpendicular to the neutron beam. The crystal was mounted inside a superconducting cryomagnet that provided temperatures down to 2 K and horizontal magnetic fields up to 6.8 T along the *c* axis. The background measured at 15 K and 0 T was subtracted from the SANS data. The 3D SANS plot is generated by using the GRIP (GRASP Integrated 3D Plotter) (*49*). Single-crystal unpolarized neutron diffraction measurements were carried out using the double focusing triple-axis spectrometer BT-7 at the NIST Center for Neutron Research (*50*) and PONTA at JRR-3. Details are described in section S5.

High–magnetic field magnetization measurements were performed in the National High Magnetic Field Laboratory (NHMFL) pulse field (Los Alamos National Laboratory) facilities. Measurements in fields up to 50 T were performed in both ^3^He and ^4^He atmospheres. Temperatures between 1.5 and 4 K were taken with the sample immersed in ^4^He liquid. High–magnetic field transport measurements were carried out in the NHMFL at the dc field (Tallahassee, FL). Measurements in the dc field up to ±31.65 T were performed using standard ac lock-in techniques. To obtained longitudinal resistivity, the signals were field symmetrized.

ARPES measurements were performed at two different beamlines of the Advanced Light Source: the beamline 4.0.3 (MERLIN) and the beamline 7.0.2 (MAESTRO). The two endstations are equipped with R8000 and R4000 hemispherical electron analyzers (Scienta Omicron), respectively. EuAg_4_Sb_2_ crystals were cleaved inside ultrahigh vacuum chambers with a base pressure better than 5 × 10^−11^ torr using conventional the top-post cleaving method. All spectra were measured with linear horizontal light polarization. The photon energy–dependent experiments were performed while tuning the photon energy from 70 to 150 eV, which covers the complete BZ of EuAg_4_Sb_2_ along *k_z_*. The Fermi surface and energy-momentum dispersions were measured with 104-eV photon that maximizes the matrix elements of the bands centered at Γ.

DFT calculations were performed with the Vienna ab initio simulation package and further processed by Wannier90 to use Wannier transformation in analyzing the electronic structure. The electronic structures of EuAg_4_Sb_2_ and SrAg_4_Sb_2_ were converged with a nonmagnetic ground state with spin-orbit coupling. In contrast, calculations with ferromagnetic EuAg_4_Sb_2_ ground states based on LDA+*U* treatment for Eu f electrons were used to motivate the magnetic interaction energies.

## Supplementary Materials

### This PDF file includes:

Sections S1 to S10 Figs. S1 to S25 Tables S1 to S3 References
