# Supplementary material for: Electronic commensuration of a spin moiré superlattice in a layered magnetic semimetal
Source: Sci Adv. 2025 Feb 5;11(6):eadu6686. doi: 10.1126/sciadv.adu6686 (PMC11797546; doi:10.1126/sciadv.adu6686)
Supplement: Supplementary file 1 — Sections S1 to S10 Figs. S1 to S25 Tables S1 to S3 References [file sciadv.adu6686_sm.pdf]

Science Advances
AAAS

Supplementary Materials for

Electronic commensuration of a spin moiré superlattice in a layered magnetic semimetal

Takashi Kurumaji *et al.*

Corresponding author: Takashi Kurumaji, kurumaji@mit.edu; Joseph G. Checkelsky, checkelsky@mit.edu

Sci. Adv. **11**, eadu6686 (2025)
DOI: 10.1126/sciadv.adu6686

This PDF file includes:

Sections S1 to S10
Figs. S1 to S25
Tables S1 to S3
References

1. Angle resolved photoemission spectroscopy of $EuAg_4Sb_2$ and comparison with density functional theory calculations for the bulk and slab geometry.

In Fig. S1, we summarize termination dependence of x-ray photoemission spectra (XPS) and band dispersions. We determined the surface termination *in situ* using XPS on Sb 4d core levels with beam spot focused down to 50×30 μm^2 as shown in Fig. S1A. For an Sb terminated surface, there are two different Sb sites on the sample, *i.e.*, one on the surface and the other in bulk. Due to different electronic potential for each Sb site, the Sb core levels show a broadened peak in XPS (blue curve in Fig. S1A) while all Sb sites experience an identical nearest-neighbor environment in the sample with Eu termination to give rise to a sharp single peak (red curve in Fig. S1A). Figures S1B-C show the angle resolved photoemission spectroscopy (ARPES) results obtained from different termination samples. Comparing the band dispersions, we confirmed that the Fermi surfaces are composed of Sb orbitals; the 4f orbitals from Eu are located approximately -1.5 eV below E_F.

The photon-energy dependent experiments were performed while tuning photon energy from 70 eV to 150 eV which covers the complete Brillouin zone (BZ) of $EuAg_4Sb_2$ along k_z. The Fermi surface and energy-momentum dispersions in the k_xk_y-plane as shown in Fig. 1C and Figs. S2C-F were measured with 104 eV photon that maximizes the matrix elements of the bands centered at Γ. We have performed temperature-dependent experiments at 15 K, 8.7 K, and 6.9 K, but did not observe any appreciable changes in the spectra (data for 15 K are shown throughout unless otherwise noted), potentially due to the incommensurate nature of the underlying superlattice potential [51] and the finite energy resolution relative to the modulation expected from the superlattice [52]. As finite magnetic field and lower temperature amplifies the transport response, experiments deeper in the ICM2 phase (or with higher resolution to capture supermodulation effects) are of significant interest.

In Fig. S2, we first compare the ARPES measurements and the first principles density functional theory (DFT) calculations of the band structure for $EuAg_4Sb_2$. The details of the DFT calculations are given in Sec. S8. Note that the *k* points here are labeled using the conventions of the hexagonal unit cell and the associated BZ is folded from the rhombohedral convention (Fig. S2A). The electronic bands here are calculated in the rhombohedral unit cell setting, which can be viewed as the unfolded electronic structure from the hexagonal setting. Therefore, the Γ and Γ’ points in Fig. S2B are inequivalent with distinct bands, and similarly for M/M’ and K’/K” point locations. The Fermi level in the DFT is adjusted to match the overall

band structure. In the comparison, we can trace the features associated with the α and β hole pockets as probed in ARPES and transport measurements.

In Fig. S3, we further analyze the k_z dependence of the characteristic band properties in the $EuAg_4Sb_2$ DFT calculation. Figure S3A shows the k_z dispersion at $k_x = k_y = 0$. No bands cross E_F so that the α and β pockets form an open Fermi surface along the k_z direction as observed in ARPES measurements. We computed the average Fermi wave vector k_F and velocity v_F as a function of out-of-plane momentum k_z. Here, we obtain k_F at each k_z by $k_F = [A(k_z)/\pi]^{1/2}$, where $A(k_z)$ is the cross section of the Fermi surface in the k_xk_y-plane at k_z, and define v_F as the average of $|v(k_x, k_y)|=1/\hbar\cdot((\partial E/\partial k_x)^2+(\partial E/\partial k_y)^2)^{1/2}$ on the Fermi surface in the k_xk_y-plane. k_F and v_F at $k_z = 0$ (Γ) and $k_z = 3\pi/c$ (A) for each pocket are tabulated in Table S1 and compared with the quantum oscillations and ARPES measurements. Figures S3D-H show the cross section of Fermi surfaces (see also Fig. 1B) at various cutting planes. The α pocket has a nearly ideal cylindrical shape as observed in ARPES measurements while the β pocket has a cylindrical shape but relatively complex warping. The γ pockets are located near the F point for the rhombohedral BZ setting and are closed electron-type Fermi surfaces. We have observed signatures of the electron pocket γ in ARPES, but with relatively weak intensity; we confirmed the contribution from electron-type carriers in transport measurements (see Sec. S2, S6).

In order to simulate the surface states in $EuAg_4Sb_2$, we have considered different slabs geometries as shown in Figs. S4A-C with different surface terminations. The ground state for these slab calculations were converged with a 9×9×1 k-grid, without spin-orbit coupling. To distinguish the surface and bulk electronic features, we define the surface region (Ag_2Sb unit(s) at the termination surface) and bulk region (in the middle) in these slabs for the wave function weight projections. The corresponding electronic band structures with the surface weight projections are shown in Figs. S4D-F. At the Fermi level, we project the spectral function in the k_xk_y-plane weighted by the surface wave function components in Figs. S4G-I. Among these types of slabs, the $Eu_4Ag_{20}Sb_{10}$ geometry with five Ag_4Sb_2 bilayer units and Ag_4Sb_2 bilayers surface terminations (in Figs. S4B, S4E, and S4H**)** shows pronounced surface state features consistent with ARPES observations (Fig. 1C).

Finally, we note that while we are able to resolve two distinct pockets α_1 and α_2 in quantum oscillations, we report only a single α pocket in ARPES. While the instrumental resolution in the present study is approximately 0.01 Å^{-1}, surface roughness, finite electron lifetime, and

other effects may increase the observed band thickness. In the present ARPES data, the k_F peak for the α pocket occurs at $k_F = 0.075$ Å^{-1} with a FWHM of 0.023 Å^{-1} (the range plotted in Fig. 2D). The pockets α_1 and α_2 observed in quantum oscillations (see Sec. S3) thus fall within the ARPES peak broadness, thus precluding their possible distinction.

2. Out-of-plane transport properties of $EuAg_4Sb_2$

Figure S5A compares the in-plane resistivity (ρ_{xx}) with out-of-plane resistivity (ρ_{zz}). ρ_{zz} is more than one order of magnitude larger than ρ_{xx} at all temperatures. The ratio ρ_{zz}/ρ_{xx} reaches 230 at the lowest temperature as shown in Fig. S5B. This value is two orders of magnitude larger than the other Eu-based layered compound $EuCd_2Sb_2$ (< 2 at 2 K, 0 T) [53], and larger than $EuAg_4As_2$ (> 100 at 1.8 K) [54]. Comparable magnitude with archetypical two-dimensional magnetic systems such as $PdCrO_2$ (> 150 at all temperatures) [55] and $EuMnBi_2$ (> 480 at 50 K, 0 T) [12] suggests consistency with the two-dimensional nature of the electronic structure in $EuAg_4Sb_2$. The anisotropy of transverse magnetoresistance (MR) is large (Fig. S5C): in contrast to almost negligible MR in ρ_{zz}, that for ρ_{xx} approaches near 800% at $T = 2$ K in $B = 9$ T. This corresponds to a mobility $\mu_{MR} \approx 3200$ cm^2/Vs via $\Delta\rho/\rho(B = 0) = (\mu_{MR}B)^2$. We observed an electron-type carrier in the Hall effect measurement for the applied charge current $I||z$ (Fig. S5D), corresponding to the Hall coefficient $R_H = -0.25$ cm^3/C. The different sign with the in-plane transport agrees with the feature that the open Fermi surfaces for α and β hole pockets would show low out-of-plane mobility in contrast with the three-dimensional electron pockets γ at the zone edges. An estimation of mobility ($\mu_{zz} = R_H/\rho_{zz}$) and $k_{F\gamma}$ (= $(3\pi^2 n_\gamma)^{1/3}$) are 565 cm^2/Vs and 0.04 Å, respectively. Here, n_γ is the charge density of each γ pocket, which is approximately given by $n_\gamma = 1/3R_He$ (note there are three γ pockets in the BZ).

3. Shubnikov-de Haas and de Haas-van Alphen oscillations of $EuAg_4Sb_2$.

Under an external magnetic field (B) applied along the c axis, ρ_{xx} shows non-saturating parabolic increase up to the highest magnetic field, $B = 31.65$ T (Fig. S6A). For $B > 5$ T, we observed Shubnikov-de Haas (SdH) oscillations (see inset); these are absent near $B \perp c$ (blue curve). Figures S6B-D summarize the angular dependence of the SdH oscillations with a single crystal sample (DCSdH-Eu#1). The orientation of the external magnetic field (B) was varied

from the c axis ($\theta_a = 0°$) towards the a axis, which is parallel to I (see the inset in Fig. S6A). To identify oscillation frequencies, we subtract a smooth background from the raw resistivity data (Fig. S6B). The Fast Fourier transformation (FFT) gives the oscillation frequencies as peaks (Figs. S6C-D). By using different magnetic-field windows for the FFT (Fig. S6E), we identified six oscillations in total at the lowest temperature with $B\|c$, where the higher frequencies, β_1, β_2, β_3 become clearer with higher-field window (blue curve in Fig. S6E). Multiple peaks between α_2 and β_1 are higher harmonics for α_1 or α_2, consistent with the exponential decay of amplitudes as a function of harmonics number (Fig. S6F). Figure S6G shows the temperature dependence of amplitudes of each oscillation branch for $B\|c$. From these data we estimate the physical parameters such as the effective mass by using a Lifshitz-Kosevich (LK) formula, which are summarized in Table S1.

Figure S6H plots the angle dependence of oscillation frequency for each branch. We note that the branches α_1 and α_2 behave in good agreement with the function ($\propto 1/\cos\theta_a$) as expected from the cylindrical Fermi surfaces. The DFT calculations reproduce the cylindrical α pocket with weak k_z-warping showing consistency with the observations (see details in Sec. S1). We observed the branch ω as a weak peak beside the α branches and its frequency shifts lower and disappears as θ_a increases. This feature suggests the three-dimensional small pockets and potentially relates with the electron pocket γ at BZ edge. All the other branches, β_1, β_2, and β_3, get higher as the magnetic field rotated towards the in-plane, reflecting the two-dimensional character of Fermi surfaces. We note that β_1 and β_2 merge at around $\theta_a = 30^\circ$. In the DFT calculations, we are able to reproduce two extrema in the Fermi surface radius for the β pocket (Fig. S3B). We assume a local plateau in Fermi surface cross section at an intermediate k_z in addition to the BZ center ($k_z = 0$) and edge ($k_z = 3\pi/c$), resulting in three separated frequency branches. We measured the SdH oscillations with two additional samples (DCSdH-Eu#2 and DCSdH-Eu#3), which provide reproducible results as summarized in Figs. S7A-F. The obtained physical parameters are tabulated in Table S1.

We observed de Haas-van Alphen (dHvA) oscillations in the magnetization measurement with a single crystal of $EuAg_4Sb_2$ (PsdHvA-Eu#4) under a pulsed field near the c axis. Magnetization curves as a function of magnetic field at various temperatures are shown in Fig. S7G. We discerned three peaks in the FFT of the oscillations (Fig. S7H), where a smooth background was subtracted, which correspond to ω, α_1, and α_2 branches observed in the SdH measurements. Again, we observed ω branch as a weak peak. Temperature dependence of

oscillation amplitude provides the estimation of the effective mass of each branch (see the inset) as described below.

The overall SdH and dHvA oscillation with multiple oscillations can be written [56], respectively, as

$$\Delta\rho = \sum_i \sum_p N_{i,p} B^{1/2} R_T^{i,p} R_\mathrm{D}^{i,p} R_\mathrm{S}^{i,p} \cos\left[2\pi\left(\frac{pf_i}{B} + \gamma_{i,p}\right)\right], \tag{S1}$$

$$\Delta M = \sum_i \sum_p N_{i,p} B^{1/2} R_T^{i,p} R_\mathrm{D}^{i,p} R_\mathrm{S}^{i,p} \cos\left[2\pi\left(\frac{pf_i}{B} + \gamma_{i,p}\right)\right], \tag{S2}$$

where $i = \alpha_1, \beta_1...$ is the index for frequency branch, $p = 1, 2\ldots$ is the number of harmonics, and $N_{i,p}$, f_i, and $\gamma_{i,p}$ are oscillation amplitude, frequency, and phase factor, respectively. $R_T{}^{i,p}$, $R_\mathrm{D}{}^{i,p}$, and $R_\mathrm{S}{}^{i,p}$ are the thermal damping factor, the Dingle damping factor, and the modulation due to spin splitting induced by magnetic field, respectively, which are given as follows.

$$R_T^{i,p} = \frac{2\pi^2 p k_\mathrm{B} T m_i^*}{\hbar e B} \sinh^{-1}\left(\frac{2\pi^2 p k_\mathrm{B} T m_i^*}{\hbar e B}\right), \tag{S3}$$

$$R_\mathrm{D}^{i,p} = \exp\left(-\frac{2\pi^2 p k_\mathrm{B} T_{\mathrm{D}i} m_i^*}{\hbar e B}\right), \tag{S4}$$

$$R_\mathrm{S}^{i,p} = \cos\left(\frac{p\pi g_i^* m_i^*}{2m_\mathrm{e}}\right), \tag{S5}$$

where $\hbar$ is the Planck constant divided by 2π, k_B is the Boltzmann constant, e (> 0) is the elementary charge, m_i^* is the effective mass, $T_{\mathrm{D}i}$ is the Dingle temperature, g_i^* is the spin-splitting factor [56].

Equation (S3) provides an LK formula to be applied in the estimation of m_i^* from the temperature dependence of oscillation amplitudes. In the fitting process, we used the magnetic field B in Eq. (S3) with the mean field $B_\mathrm{av} = [(B_\mathrm{min}{}^{-1} + B_\mathrm{max}{}^{-1})/2]^{-1}$ for the FFT window between the minimum (B_min) and the maximum (B_max) field. We also estimated the radius of Fermi surface $k_{\mathrm{F}i}$ by assuming a cylindrical Fermi surface: oscillation frequency f_i and the area of the extremal orbit of the Fermi surface A_i are related through $A_i = 2\pi e f_i/\hbar$, and thus $k_{\mathrm{F}i}$ is calculated

with $A_i = \pi k_{Fi}^2$. Fermi velocity v_{Fi} can also be calculated through $v_F = \hbar k_{Fi}/m_i^*$. The obtained physical parameters are summarized in Table S1.

We also performed the fit of the overall raw oscillation data at the measured lowest temperature using Eqs. (S1-S2) as shown in Fig. S8. The fitting results at the measured lowest temperatures agree for each sample, which enables us to estimate the Dingle temperature T_D as well as the quantum lifetime. We confirmed the fitting quality is not affected by the fitting equations with or without higher harmonics terms. The quantum lifetime τ_{qi}, quantum mobility μ_{qi}, and mean free path l_{qi} for each branch i are calculated from the obtained Dingle temperature with $T_{Di} = \hbar/2\pi k_B \tau_{qi}$, $\mu_{qi} = e\tau_{qi}/m_i^*$, and $l_{qi} = v_{Fi}\tau_{qi}$, where m_i^* and $v_{Fi} = (2\hbar e f_i)^{1/2}/m_i^*$ are also obtained in the same fitting base. Table S1 includes the summary of the physical parameters for this analysis.

Table S1 summarizes the corresponding parameters estimated by ARPES measurements and DFT calculations (see details in Sec. S1 and S8). We have confirmed that the estimations of the size of the α pocket are consistent with those in the quantum oscillation measurements within the range of uncertainty (see Fig. 2D). The Fermi velocities are also consistent within an order of magnitude.

4. Magnetization and specific heat measurements for the magnetic phase diagrams of $EuAg_4Sb_2$.

Figures S9A-D summarize magnetization data for a single crystal of $EuAg_4Sb_2$. We confirmed easy-plane type magnetic anisotropy in $EuAg_4Sb_2$ via magnetization measurements. This feature can be known from the moderate temperature dependence M/H_{ext} for $B||c$ below the magnetic transition temperatures in contrast to the behavior in that for $B||a$ (Fig. S9E). M/H_{ext} for $B||a$ keeps decreasing below T_{N3} = 10.6 K and shows large drop at T_{N1} =7.5 K, suggesting the evolution of in-plane component of Eu spin moments. It is consistent with the anisotropy of saturation magnetic field, which are estimated as being B = 3.2 T for $B||c$ and B = 1.1 T for $B||a$ (Fig. S9F) at T = 2 K. Similar feature has been observed in the arsenic isomorph [14,16,54,57]. Magnetic susceptibility gives an anisotropic effective magnetic moment, p_{eff}, and Weiss temperature, Θ_W: $p_{eff||c}$ = 8.53 μ_B, $p_{eff||a}$ = 8.85 μ_B, $\Theta_{W||c}$ = 8.25 K, and $\Theta_{W||a}$ = 10.64 K. The saturation magnetic moment (M_s) is estimated as being $M_{s||c}$ = 7.81 μ_B, and $M_{s||a}$ = 8.04 μ_B, which are larger than free atomic value 7 μ_B.

The specific heat (C_p) measurement at zero field (Fig. S9G) identifies two first-order magnetic transitions at T_{N3} and T_{N1}, and a transition at T_{N2} = 9.5 K identified by a weak kink in dC_p/dT (Fig. S9H). These critical temperatures coincide with those obtained in the magnetic susceptibility measurements (Figs. S9C-D) where dM/dT at B = 0.1 T shows two sharp peaks at T_{N1} and T_{N3} and weak peak at T_{N2}. We estimated the specific heat around T_{N1} and T_{N3} for $EuAg_4Sb_2$ by an analysis of relaxation curve of the sample temperature under the assumption of an infinite thermal conductivity between sample and platform following Refs. [58,59]. The specific heat of the sample C_x can be estimated by:

$$C_x(t) = \left\{\frac{P(t) - K_1[T_p - T_0]}{dT_p/dt}\right\} - C_a, \qquad \text{(S6)}$$

where $P(t)$ is the time (t) dependence of heater power, K_1 is the thermal conductivity between heat bath and the platform, $T_p(t)$ is the platform temperature, T_0 is the heat bath temperature, and C_a is the platform specific heat. $K_1(T)$ and $C_a(T)$ were obtained from the addenda measurements and can be assumed as constants within the temperature change for the heat pulse. The phase boundary between ICM2 and ICM3 at T_{N2} = 9.5 K is not clearly identified in ρ_{xx} or $d\rho_{xx}/dT$ (Fig. S9I) while the corresponding anomaly in the field-scan of ρ_{xx} and ρ_{yx} is more pronounced as discussed in Sec. S6.

The magnetic phase diagram for $B \| c$ is shown in Fig. S9J. The phase boundaries are determined by kinks in the raw data (Figs. S9A, S9C) and peaks in dM/dB (Figs. S9B and S9F) or dM/dT (Fig. S9D) (herein we use $B = \mu_0 H_{ext}$, with H_{ext} being the applied magnetic field) as well as the field-derivative of magnetotransport properties (Fig. S20). We identified three magnetically modulated phases, ICM1, ICM2, and ICM3, in addition to the paramagnetic phase (PM) and field-induced ferromagnetic phase (FM). We note that the arsenide sibling compound [60] has also been reported to show a similar magnetic phase diagram as well as the magnetoresistance behavior associated with the metamagnetic transitions. Due to the disorder at the silver sites, the arsenide shows a relatively reduced electron quality as judged from RRR and the parabolic MR at high field. In this sense, we can expect to reveal the impact of defects on the coupling between magnetism and conduction electrons through a comprehensive investigation in $EuAg_4(As,Sb)_2$.

5. Small-angle neutron scattering and neutron diffraction on $EuAg_4Sb_2$

In order to understand the difference between the spin structures in $EuAg_4Sb_2$, we performed SANS experiment using the SANS-I instrument at the Paul Scherrer Institut (PSI). Figures S10A-F summarize the SANS pattern obtained in a cooling process from the paramagnetic state at zero field. The neutron beam is directed near parallel to the c axis, and the intensity was integrated in rocking-scans. The wavelength of neutrons is $\lambda_n = 5$ Å. A background pattern taken at $T = 15$ K in zero field is subtracted from all plotted data. We observed the evolution of the SANS patterns from ICM3 (Figs. S10A-B), to ICM2 (Figs. S10C-D), and eventually to ICM1 (Figs. S10E-F) as described in the main text (Figs. 1E-G). We introduce Cartesian coordinates (q_x, q_y, q_z) for the magnetic modulation in the sample, where q_x and q_z are parallel to the a^* and c^* axes, respectively. In addition to the dominant q_xq_y component, we observed a small but nonzero q_z component for the magnetic modulation vectors, q_{ICM1}, q_{ICM2}, and q_{ICM3} as summarized in Fig. S10G. The hexagonal pattern for $q_{\mathrm{ICM3'}}$ in the ICM3 state is confined to the a^*b^*-plane. The in-plane component of the $\boldsymbol{q}$ vectors, $q_{xy} = (q_x^2 + q_y^2)^{1/2}$, are summarized in Fig. S10H. We note that $q_{\mathrm{ICM3}xy}$ and $q_{\mathrm{ICM3'}xy}$ are proximate in length to $q_{\mathrm{ICM1}xy}$ and $q_{\mathrm{ICM2}xy}$, respectively. As such, the ICM3 state has a more complex structure than ICM2; further polarized neutron studies are highly desired to evaluate its structure. Figure S10I gives the in-plane orientation of the q vectors measured as an angle φ_q from the a^*-axis (see inset of Fig. S10D). Here q_{ICM2} is rotated away from the a^* axis by approximately $\varphi_q = \pm 15°$. The thermal evolution of the scattering intensity clearly reflects the phase transitions detected by thermodynamic and magnetic properties measurements as summarized in Fig. S10J.

After zero-field cooling, we measured the field-induced phase transitions upon increasing field at $T = 2.1$ K for $B \| c$ as described in Figs. 1H-J. Figures S11A-F capture the successive transitions from ICM1 (Figs. S11A-B) to ICM2 (Figs. S11C-D), which is mixed with the ICM3 state (Fig. S11E), and eventually to the pure ICM3 state (Fig. S11F) before entering the FM state above $B = 3.25$ T. The field-induced changes of q_z and φ_q are summarized in Figs. S11G-H (see Fig. 3A and Fig. 2C for integrated intensity and q_{xy}, respectively). Figures S11I-K show q_z dependence of the radial-integrated intensity for each phase.

While these q-vectors are largely in the a^*b^*-plane, they have a finite component along the c^* axis. The complete scattering patterns (azimuthal angle (φ) dependence and the three-dimensional peak positions) for all the ICM states are shown in Figs. S12A-F, which are reproduced from the rocking-scan of the SANS scattering pattern with magnetic field fixed

along the c axis of the crystal. There are 24 magnetic Bragg spots for the ICM2 state as schematically depicted in Fig. S12E, reflecting the domains of the double-q structure with monoclinic in nature breaking three-fold rotational symmetry of the underlying crystal.

To check the multi-q nature of the ICM2 state, we investigated the SANS pattern as a function of field tilt angle η from the c axis. The experimental geometry is shown in Figs. S13A-C. Multi-q structures are often observed to be favored by field applied normal to the multi-q vector ($\boldsymbol{n}$) [17, 61, 62], *i.e.*, domains with larger $(\boldsymbol{n}\cdot\boldsymbol{B})^2$ are favored [63]. Due to the finite q_z component, $\boldsymbol{n}_{\mathrm{A}}$, for example, is slightly tilted away from the exact $\boldsymbol{c}^*$ axis towards the -$\boldsymbol{a}^*$ direction (see Fig. S13D). Tilting the field towards $\boldsymbol{n}_{\mathrm{A}}$ is expected to select the A domain. To further lift the degeneracy of the ICM2-domains under the tilt-field, the field is rotated within the plane (see the cyan plane in Fig. S13C) that is not the exact a^*c^*-plane, but rotated by $\psi \sim +14°$ around the c axis. At $\eta \neq 0$, the in-plane component of the field (B_{in}) is deviated from $\pm\boldsymbol{a}^*$ axis. Figure S13E is a picture of the single crystals for the SANS measurements. We used the sample SANS-Eu#2 for the tilt-field experiment. As shown in Figs. S13F-G, the SANS pattern of the ICM1 state is rotated by $\psi = 13.8°$, compared to the sample SANS-Eu#1 with $\psi = 0°$. In this experiment, we used a shorter wavelength neutron beam $\lambda_{\mathrm{n}} = 3$ Å.

We zero-field cooled (ZFC) the sample and then applied $B = 2.2$ T at $T = 1.5$ K with $B||c$ (defined as $\eta = 0°$, Fig. S14A). The field is then tilted to $\eta = 12°$ (Fig. S14B). Figures S14B-H show the sequential evolution of the Bragg spots at each η upon subsequent tilting back towards $\eta = -12°$ (with fixed T and magnitude of B). As shown in Fig. S14A, we observed a multiple peak structure consistent with ICM2. Notably, the identified (ICM2) peaks disappear at $\eta = 12°$ (Fig. S14B) and are replaced with single-q domain states (lime green and yellow, which are nearly related with each other by $C_2||\boldsymbol{a}^*$ axis). Figures S14C-E show the corresponding evolution tilting back to $\eta = 0°$. The single-q state is replaced by the spontaneous appearance of the multi-q pattern as denoted by four-fold peak patterns color-coded by pink (domain A) and black (domain C').

For the ICM2 state, a single-q proper screw structure is excluded as the q-vectors A_1 and $\mathrm{C'}_2$ survive (see Figs. S14I-J). These are expected to be the most unstable q vectors under the present tilt field unless they belong to a group of q-vectors composed of a multi-q state. Similarly, a single-q cycloidal structure is also excluded. The triple-q state composed of q

vectors oriented 120° away from each other is also unlikely because the mutual positions of q vectors A_1, A_2, C'_1 and C'_2 are incompatible with C_3 symmetry.

Instead, the double-q state can provide a consistent explanation for the experimental results. First, twenty-four peaks are decomposed to six domains as shown in Fig. S14I. We note three domains, A, B, C (Fig. S14I-left) are connected to the domains A', B', C' (see Fig. S14I-right) by the C_2 rotation around the a^* axis, which is due to twinning of the rhombohedral crystal (obverse-reverse twin). The domains A and C' are stabilized by the tilted field (see Fig. S14J). The selected two domains, A and C', have the in-plane component of the $\boldsymbol{n}$ vectors, n_{Ain} and $n_{\mathrm{C'in}}$ towards B_{in} because of the finite q_z component (see Fig. S14I; plus/minus marks indicate the sign of q_z for each Bragg spot). This means that they are the two domains with $\boldsymbol{n}$ relatively closer to the $\boldsymbol{n}||\boldsymbol{B}$ condition in the $\eta > 0$ configuration.

Figures S14E-H shows the evolution in the field-tilting to the opposite direction. We note that for $\eta = -3°$ (Figs. S14G) domains A and C' are substantially weakened and instead domains C and A' with n_{in} towards B_{in} are most favored as shown in Fig. S14K. Faint contributions from domain B' remain because the orientation of $n_{\mathrm{B'in}}$ near perpendicular to $-B_{\mathrm{in}}$ is marginally influenced by the field tilt. Figure S14L shows the representative η dependence of the integrated intensity for the Bragg spots A_1 and A_2. The A_1 and A_2 peak intensities evolve collectively with respect to η even though the finite ψ breaks the configurational symmetry relative to B_{in}, indicating that they belong to the same double-q domain. The intensity of the peak is not symmetric with respect to η because $(\boldsymbol{n}_{\mathrm{A}}\cdot\boldsymbol{B})^2$ takes the maximum at positive η due to the intrinsic tilt of $\boldsymbol{n}_{\mathrm{A}}$.

We also performed a single-crystal neutron diffraction study using a triple-axis spectrometer (TAS) BT-7 at NIST Center for Neutron Research, and a triple-axis spectrometer PONTA in Japan Research Reactor 3 (JRR-3). For the former, the spectrometer is equipped with a position sensitive detector and the incident neutron beam was monochromatized at λ_{n} = 1.64 Å (E_{n} = 30.5 meV) using the (002) reflection of pyrolytic graphite PG monochromator. We measured the magnetic satellite peaks around (H, K, L) = (0, -1, 1) to search for the incommensurate magnetic modulations in the ordered phases at zero field, ICM1, ICM2, and ICM3 (see the scattering intensity profile in Figs. S15A-C). The positions of the q vector for the ICM2 and ICM3 are consistent with the SANS measurement while the transition between ICM2 and ICM3 or finite q_z component was not clearly resolved due to the wider resolution function of thermal neutrons. The apparent double-q spot in ICM1 state can be understood as

projected scattering from other q-vectors forming the six-fold-q which are away from the scattering plane. The temperature dependence of the q positions and integrated intensity is summarized in Figs. S15D-F. For the PONTA in JRR-3, the incident neutron beam with the energy 14.7 meV was obtained by a Heusler (111) crystal monochromator. We measured the magnetic satellite peaks around (0, 0, 6), and observed the incommensurate modulations at zero field, consistent with the observations by BT-7.

We note that in the case of the current system, magnetic frustration [64] plays an important role. The complete understanding of the phase diagrams of such systems including centrosymmetric skyrmion-hosting Gd_2PdSi_3 [17], $Gd_3Ru_4Al_{12}$ [18], $GdRu_2Si_2$ [19], and $EuAl_4$ [20] is an ongoing theoretical challenge. The phase diagram of $EuAg_4Sb_2$ is reminiscent of that in noncentrosymmetric GaV_4Se_8 [65], where an in-field skyrmion-lattice state is directly connected to the FM state, compared with others such as MnSi and Gd_2PdSi_3 which have another transition to a single-q state. The main double-q-phase, ICM2, is characterized by a slope change at the transition from the ICM1 state in the magnetization as shown in Fig. S9F (note that the heights of dM/dB are different for the ICM1 and ICM2 states) while another possible multi-q phase, ICM3, does appear adjacent to the FM phase. It is also notable that ICM2 extends to $B = 0$. We hypothesize this may connect to recent theoretical proposals for multi-q phases in itinerant, centrosymmetric systems [66-68] and *e.g.* recent experiments in Gd_2PdSi_3 reporting a zero field triple-q (meron-antimeron) lattice state at zero field [17]. Understanding of the magnetic phase diagram may require establishing the appropriate spin Hamiltonian and interaction terms (*e.g.* RKKY interactions [5,66], local/interaction anisotropy [69,70], dipole-dipole interactions [71], and higher-order spin-spin interactions [72], and competition among different spin modulations [73]).

Here, we discuss candidate spin configurations for the ICM2 state. First, for simplicity, we consider two-dimensional textures and simplify the rhombic tilting between q_1 and q_2. We simulate an orthogonal double-q spin texture in 2D space using the ansatz below.

$$\boldsymbol{S}(x,y) = \mathrm{Re}\left[\frac{1}{2}\left(0,-\mathrm{i},S_{z,q}\right)\exp(\mathrm{i}qx) + \frac{1}{2}\left(\mathrm{i}h,0,S_{z,q}\right)\exp(\mathrm{i}qy) + \left(0,0,S_{z,0}\right)\right], \quad \text{(S7)}$$

where the first and second terms represent the double-q modulation along the x and y axes, respectively. The real variable $S_{z,q}$ describes the out-of-plane component of helical spin

modulation. h (= ±1) in the second term represents the handedness of helical spin modulation of $q||y$ identical or opposite to that for $q||x$. The third term is the homogeneous spin moment ($S_{z,0}$) induced by *e.g.*, an applied magnetic field. By fixing h = +1 and choosing $S_{z,q}$ and $S_{z,0}$, a square skyrmion lattice, a meron-antimeron lattice, and a vortex lattice as shown in Figs. S16A-F are produced. We also calculate the topological number (n_{sk}) as a function of $S_{z,0}$ and $S_{z,q}$ as shown in Figs. S16G using the formula below.

$$n_{\mathrm{sk}} = \frac{1}{4\pi}\int \boldsymbol{n} \cdot \left(\frac{\partial \boldsymbol{n}}{\partial x} \times \frac{\partial \boldsymbol{n}}{\partial y}\right) \mathrm{d}x\mathrm{d}y. \quad \text{(S8)}$$

We note that a simple harmonic superposition of two helices ($S_{z,0}$ = 0) does not produce a skyrmion lattice state. This is because some magnetic sites may have a vanishing $\boldsymbol{S}(\boldsymbol{r})$ to become a singularity point in the spin texture (Fig. S16C). Instead, it becomes a meron-antimeron lattice state, with diverging skyrmion density ($\phi_{\mathrm{sk}} = \boldsymbol{n} \cdot \left(\frac{\partial \boldsymbol{n}}{\partial x} \times \frac{\partial \boldsymbol{n}}{\partial y}\right)$, where $\boldsymbol{n} = \boldsymbol{S}/|\boldsymbol{S}|$) as depicted in Fig. S16D. The divergences cancel with each other in the spatial integration of ϕ_{sk}. The emergent magnetic field also cancels on average. A square skyrmion lattice state (Fig. S16A) arises in an applied magnetic field when the finite moment ($S_{z,0}$) is induced at the singularity points on a meron-antimeron lattice, and the topological number becomes nonzero (Fig. S16G). We note that the flipping of the handedness of the $q||y$ modulation to h = -1 gives rise to the antiskyrmion lattice state, which has the opposite topological number to that of the skyrmion lattice state. By setting the modulation of the out-of-plane moment zero ($S_{z,q}$ = 0), the spin state becomes a square lattice of vortices with topological number zero. The above discussion is unchanged with respect to the introduction of the finite q_z and the rhombic canting ($\Phi \neq 90°$) between $\boldsymbol{q}_1$ and $\boldsymbol{q}_2$. We show candidate real-space spin configurations for the ICM2 state under a magnetic field in Figs. S17A-C. Aside from q_z and Φ, these correspond to the SMSs schematically depicted in Figs. 4B-D.

6. Magnetotransport properties of $EuAg_4Sb_2$

Figure S18A shows the temperature dependence of in-plane resistivity of $EuAg_4Sb_2$ at zero field. The curve is linear down to the magnetic transitions, which clearly manifest themselves as sharp features, followed by a smooth decrease at the lowest T. Figures S18B-C show the magnetic field dependence of transport properties at various temperatures. At T = 1.8 K, for example, ρ_{xx} shows an increase as entering the ICM2 phase under $B||c$ around B = 2 T (Fig.

S18B). This resistivity increase is accompanied by a Hall resistivity anomaly (Fig. S18C), while the low field Hall coefficient for $|B| < 0.1$ T is nearly temperature independent (see Fig. S18A). Above T_{N3}, at $T = 15$ K, ρ_{xx} shows negative magnetoresistance in low field.

To estimate carrier density and mobility of carriers in $EuAg_4Sb_2$ with two-band model, we converted ρ_{xx} and ρ_{yx} into longitudinal conductivity (σ_{xx}) and Hall conductivity (σ_{xy}) through $\sigma_{xx} = \rho_{xx}/(\rho_{xx}^2 + \rho_{yx}^2)$ and $\sigma_{xy} = \rho_{yx}/(\rho_{xx}^2 + \rho_{yx}^2)$. Within the two-band model, σ_{xx} and σ_{xy} are given by

$$\sigma_{xx} = \frac{n_{\mathrm{e}} e \mu_{\mathrm{e}}}{1 + (\mu_{\mathrm{e}} B)^2} + \frac{n_{\mathrm{h}} e \mu_{\mathrm{h}}}{1 + (\mu_{\mathrm{h}} B)^2}, \quad \text{(S9)}$$

$$\sigma_{xy} = -\frac{n_{\mathrm{e}} e \mu_{\mathrm{e}}^2 B}{1 + (\mu_{\mathrm{e}} B)^2} + \frac{n_{\mathrm{h}} e \mu_{\mathrm{h}}^2 B}{1 + (\mu_{\mathrm{h}} B)^2}, \quad \text{(S10)}$$

where n_e and n_h are carrier density and μ_e and μ_h are mobility for electron-type and hole-type carriers, respectively. As summarized in Figs. S18D-E, the field dependence of σ_{xx} and σ_{xy} show typical two-carrier feature except for the anomaly associated with the ICM2 and ICM3 states. We simultaneously fit σ_{xx} and σ_{xy} (except for the ICM2-ICM3 region) with Eqs. (S9-S10) up to $T = 7$ K (above this negative MR complicates the analysis). In Figs. S18D-E, the resultant fitting curves are also shown. The fittings show agreement except for the ICM2-ICM3 region. The temperature dependence of the fitting parameters are plotted in Figs. S18F-G. The mobility of the hole bands reaches 5000 cm^2/Vs at the lowest temperature.

Phenomenologically, we can reproduce the jump of the longitudinal and transverse responses across the transition from the low field state (ICM1) to the intermediate-field SMS state (ICM2) based on our two-band model (Eqs. (S9-S10)), where the conductivity tensors, σ_{xx} and σ_{xy}, are functions of carrier density (n) and mobility (μ). We note that the SMS formation opens a gap at one of the hole bands, α pocket, and renormalized mass enhancement causes the reduction of mobility through $\mu = e\tau/m_{\mathrm{eff}}$, where τ is carrier relaxation time and m_{eff} is the effective mass. As a result, both σ_{xx} and σ_{xy} are expected to show a sharp drop at the entering the ICM2 state from the ICM1 state, which are indeed consistent with what we observed in experiments (see Fig. 3C and S18D-E). This prominent change of σ_{xy} (~ 10^5 S/cm) masks any expected topological Hall response (estimated upper bound as $\sigma_{xy}^{\mathrm{T}} \sim 500$ S/cm from $e^2/h{\cdot}d$, where d is the layer distance). We can probe the mass enhancement due to the miniband formation with suppressed dispersion by extracting the resistivity jump upon entering the ICM2

phase. We plot the temperature dependence of the MR peak of Fig. 3B in Fig. S18H, which corresponds to the temperature evolution of m_{eff}/n, from $\rho \sim m_{\mathrm{eff}}/ne^2\tau$ and MR $\sim \Delta\rho/\rho_0 \propto m_{\mathrm{eff}}/n$. The MR increases below the magnetic transition temperature, suggesting a gap opening as well as a resultant mass enhancement. This is in contrast to a scattering mechanism, which would saturate at low temperature for disorder scattering.

In order to simulate the band reconstruction effect, we introduce a field-dependent effective mass (inversely proportional to mobility) and carrier density. To do this, we employ a phenomenological assumption that the field-dependence of the MR (= $\Delta\rho_{xx}/\rho_0$) can be used as a scale for n_{h} and μ_{h}: $n_{\mathrm{h}}/n_{\mathrm{h0}} = 1 - a\cdot$MR; $\mu_{\mathrm{h}}/\mu_{\mathrm{h0}} = 1 - b\cdot$MR, where n_{h0} and μ_{h0} are carrier density and mobility for hole carriers without the band reconstruction, and the parameters a and b are factors determined in the fitting. The physical meaning of this parameterization is that n_{h} and μ_{h} are renormalized by the electronic commensuration where a captures the drop in carrier density due to gap opening at $2k_{\mathrm{F}} = q$ and b that from changes in the effective mass due to band folding. We note that the aim is to demonstrate how a change in n_{h} and μ_{h} can affect the Hall conductivity. While this introduces additional parameters, these are constrained given the MR = $1 - n_{\mathrm{h}}/n_{\mathrm{h0}}\cdot\mu_{\mathrm{h}}/\mu_{\mathrm{h0}} \sim (a + b)$MR and thus $a + b = 1$. With this simplification, we obtain the fitting results in Fig. S19. We note that obtained parameters only provide a phenomenological description of the Hall response; further refinement requires additional investigation of the microscopic behavior of the ICM2 phase.

As shown in the phase diagram (Fig. S9J), we plot the phase boundary between ICM2 and ICM3 captured by the field scans of magnetotransport properties. We show the field-derivative of ρ_{xx} and ρ_{yx} in Figs. S20A-B. Anomalies are marked by arrows as seen by a small peak or kink, which corresponds to the subtle anomalies in temperature/field-derivative of magnetization (Figs. S9D and S9F) and specific heat (Fig. S9H).

7. Transport properties and Fermi surfaces of nonmagnetic isomorph $SrAg_4Sb_2$.

$SrAg_4Sb_2$ is isostructural with $EuAg_4Sb_2$ [13,22] and can be viewed as nonmagnetic analogue as it shows comparable qualities in terms of electron transport properties and Fermi surfaces. A single crystal of $SrAg_4Sb_2$ shows metallic resistivity as shown in Fig. S21A without signature of structural phase transition as reported in $SrAg_4As_2$ [74]. It is consistent with

previous report in terms of fixed silver atomic positions for $SrAg_4Sb_2$ in a unit cell [13]. RRR and ρ_{xx} at T = 1.8 K are 32 and 3.1 μΩcm, respectively, showing similar values with those for $EuAg_4Sb_2$. The Hall coefficient R_H (Fig. S21B) is nearly temperature independent as also confirmed in Eu compounds (Fig. S18A). We fit both σ_{xx} and σ_{xy} as a function of a magnetic field for $B||c$ with two band model at the same time as shown in Figs. S21C-D. The agreement of fitting is reasonable for all the measured temperatures. Fitting parameters are summarized in Figs. S21E-F. Electron and hole type carrier densities (n_e and n_h) are moderately compensating at 3.2×10^{20} and 3.5×10^{20} cm^{-3}, respectively, at T = 2 K. At the lowest temperature, mobilities are 2.4×10^{3} and 4.9×10^{3} cm^2/Vs for electron and hole carriers, respectively. Both carrier density and mobility for each carrier type show the same order of magnitudes with those of Eu compound.

Figure S21G summarizes the angular dependence of the SdH oscillation frequencies in $SrAg_4Sb_2$. We identified four main branches (α_1, α_2, β_1, and β_2) as well as the second harmonics from one of the branches. All of the observed branches show the quasi-2D nature as being well described by the solid curves ($\propto 1/\cos\theta_a$). We also confirmed the cylindrical hole pockets along the Γ-A line by DFT calculations (inset of Fig. S21G). Physical parameters for $SrAg_4Sb_2$ obtained in the SdH oscillation measurements and DFT calculations are summarized in Table S2; the electronic structure for Sr compound is similar with that for the Eu variant.

8. Details of density functional theory calculations: Wannier functions and exchange coupling.

The DFT calculations were performed with the Vienna ab initio simulation package (VASP) [75,76] for $EuAg_4Sb_2$ and $SrAg_4Sb_2$ crystals. The computations employed are based on the pseudopotential formalism and the Projector Augmented-Wave method [77]. For the Eu atoms, we used the pseudopotential with frozen 4f electrons for the divalent oxidation state (Eu^{2+}) in the non-magnetic ground state. Whereas in the ferromagnetic electronic state, the pseudopotential with the 4f orbital states is used with an additional onsite Hubbard $U\sim 4.5$ eV based on the DFT+U approach [78], here they are chosen such that 4f levels are located around -1 to -1.5 eV as observed in the ARPES measurements (see Fig. S1B). These computations were converged with exchange-correlation energy functional parametrized by Perdew–Burke-

Ernzerhof (PBE) [79], a Γ-centered 11×11×11 Monkhorst-Pack k-mesh grid [80], an energy cutoff 320 eV, with and without the relativistic spin-orbit coupling terms.

Figure S22 shows the orbital character of each band along K-Γ-M. The valence band at the Γ point that generates the α pocket (α band) is mainly composed of the $5p_z$/5s orbitals (Figs. S21G, and S21I) of anionic Sb atoms. A finite component from core electrons at Ag-$4d_{z2}$ orbital (Fig. S22A) and unoccupied Ag-$5p_z$/5s orbitals (Fig. S22D, and S22F) are due to the hybridization at the Sb-Ag bond in the c direction (see the bonding in Fig. 1A).

In order to gain further insights into the electronic properties of the electron pockets near $k_x = k_y = 0$, we perform Wannier analysis [81] of the converged DFT calculations. Due to the quasi-2D nature, we only consider the partial Wannier transformation in the k_z direction, which gives a basis localized only in the z axis, but extended in the xy 2D plane. Such a choice of basis states can give us insights into the chemical bonding for the itinerant electrons, and the coupling to the local magnetic moments on the Eu atoms. This is crucial to the enhanced magnetic exchange couplings, which enables band folding and band flattening of the conduction electrons through the spin moiré superlattice formation.

We then examine the electronic states without SOC for simplicity, and compare them with the bands with SOC in Fig. S23A. Despite the SOC gaps at $k_x = k_y = 0$, the pockets observed are derived from the states (Ψ_α and Ψ_β for the singlet and doublet states, respectively). With SOC, the wave functions are augmented by the spinor components. Therefore, we can simplify the orbital wave function analysis by focusing on the non-SOC states, and the wave function evolutions with k_z at $k_x = k_y = 0$. To derive the Wannier functions in k_z, we performed the Wannier transformation implemented in Wannier90 code [82], with a 1×1×9 grid (which limits the analysis to the states with $k_x = k_y = 0$ and transform only along k_z). The bands along the Γ-A line are shown in Fig. S23B, which defines the projection space. The initial atomic projectors used are the Eu s and p_x/p_y orbitals for states respectively. These Eu orbitals together with those for Sb are shown to have non-zero weights for the states in Fig. S23C. The derived quasi-2D Wannier functions are shown in Figs. S23D-E. From the derived wave functions, the Eu s or p_x/p_y orbitals couple to the wave functions from Ag_2Sb layers both above and below the Eu layer. The weights decay quickly outside of the Ag_2Sb-Eu-$SbAg_2$ block.

These results imply that the Ag_2Sb layer prefers binding with another Ag_4Sb_2 layer across the Eu layer, rather than the closest Ag_2Sb layer within the same Ag_4Sb_2 bilayer unit. This

coupling involves the intermediate Eu orbitals. Since the local magnetic moments reside on the Eu atoms, such chemical bonding can facilitate the proximity effects in magnetic exchange coupling, compared to other heterostructure systems where the wave functions for the itinerant electrons and local moments occupy different spatial regions of the heterostructure [83].

To further support this bonding picture, we analyze the complex phase ($e^{i\phi}$) of the wave function on the Ag_2Sb layers. For an Ag_2Sb unit, this is dominated by the Sb 5s component, and we measure the relative phase along k_z. We found that the relative phase (ϕ_1) in Fig. S23F stays approximately constant for the two Ag_2Sb blocks across the Eu spacer layer, which suggests a robust chemical bonding in between. On the other hand, two Ag_2Sb layers within the bilayer unit show a 2π phase winding in ϕ_2 under k_z evolution across the BZ, which rules out binding between them. This agrees with the Wannier-function based analysis above for the chemical bonding formations.

The quasi-2D nature of the α and β pockets can be understood from the viewpoint of chemical bonding. In the view of $SbAg_2$-Ag_2Sb bilayer unit, the top and bottom Sb atoms are connected with different Ag atoms (Fig. S23G). This feature weakens the intralayer coupling of Sb-5p_z orbitals resulting in the moderate dispersion along the k_z direction (Fig. S3A). One may invoke a similar argument in the context of the Sb-5p_x/5p_y orbitals for the β pocket band. This quasi-2D nature in $EuAg_4Sb_2$ is distinct from the structural sibling compound $EuCd_2Sb_2$, where Sb-5p orbitals has large dispersion along the k_z direction and cross E_F [84]. The crystal structure of the latter is obtained from the former by "merging" two Ag sites in Ag_4Sb_2 unit [13], which potentially leads to stronger intralayer coupling between Sb sites in the Cd_2Sb_2 unit by shortening the electron hopping path from Sb-Ag-Ag-Sb to Sb-Cd-Sb.

This unusual bonding pattern also occurs in other materials such as Fe_3Sn_2 [85] where two kagomé layers across the Sn spacer layer in the middle for the bonding and anti-bonding pairs for the Dirac cone states, rather than the two closest kagomé layers within the same bilayer unit. This bonding state in Fe_3Sn_2 enhances the SOC in the Dirac states from the coupling to the spacer Sn layers. The result in $EuAg_4Sb_2$ is the enhancement of the wave function overlap and energy scales in the proximity effects in deriving magnetic coupling to the conducting electrons. It is of significant interest to consider generalizations of such coupling mechanism in other heterostructure systems to enhance proximity effects.

To model the electronic structure with magnetic exchange coupling to the Eu layers, we consider ferromagnetic states. In Fig. S24A, we first show the electronic band structure with SOC corrections, assuming a non-magnetic ground state. In Fig. S24B, the electronic structure is calculated assuming a ferromagnetic state (FM). The group of bands near E_F = -1 eV to -1.5 eV are the Eu f orbitals which are not included in the non-magnetic ground state calculations in Fig. S24A (Eu^{2+} pseudo-potential is used). For the converged magnetic ground states, the magnetic moments for Eu ions are $\langle -2gS_z \rangle \approx 7\ \mu_B$. Figure S24B also shows the magnetic-moment projections for these spin split bands. The majority (minority) spin states are in red (blue). From the band spin splitting, the signs for the magnetic exchange couplings between the local Eu moment and conduction spins can be determined to be ferromagnetic, which shows a sign flipping in the spectrum moving away from the α pocket. From the orbital projections in Fig. S22, we attribute this to the changes in the wave function orbital contents (Ag and Sb) which affects the magnetic coupling to Eu atoms. Due to such magnetic interactions, the bands become spin split, and the magnetic coupling can be modeled as an effective Zeeman term. For the α bands, the exchange splitting energy J at Γ is 119.7 (122.0) meV when the FM moment is out-of-plane (in-plane).

For the multi-q textures, the local magnetic moment rotates slowly in real space, with a wavelength longer than the microscopic lattice constants. Within the local approximation, we can extend the DFT calculations to consider ferromagnetic states with rotated magnetic orderings. These parameters serve as the inputs for deriving the low-energy $k{\cdot}p$ model to be discussed in Sec. S10.

The electron-spin coupling constant J is experimentally determined by spin-disorder scattering in the paramagnetic region in zero field. Following the Matthiessen rule, we assume the resistivity is the sum of residual resistivity ρ_0, phonon-resistivity ρ_{ph}, and magnetic resistivity ρ_{mag}: $\rho = \rho_0 + \rho_{ph} + \rho_{mag}$. The ρ_{mag} for $EuAg_4Sb_2$ is obtained from ρ by subtracting $\rho_0 + \rho_{ph}$, which is evaluated from $SrAg_4Sb_2$ (see Fig. S24C). The magnetic scattering time in semimetals or semiconductors can be calculated according to the theory developed in Ref. [86] which we generalize to the 2D case, expressing the mobility in terms of the electron-spin coupling constant J as

$$\mu_{xx} = \frac{8\pi\hbar^3}{6\pi/c} \frac{e}{m_{\mathrm{eff}}^2} \left(\frac{Ng\mu_{\mathrm{B}}S}{J}\right)^2 \frac{1}{k_{\mathrm{B}}T(\chi_z + 2\chi_x)}, \tag{S11}$$

where we consider a cylindrical FS of the length $6\pi/c$, m_{eff} is effective mass, N is the number of magnetic ions in a unit volume, J is the same as defined in Eq. (1), S is 7/2, and χ_z and χ_x are magnetic susceptibility along the c and a axes, respectively. Introducing the average effective mass for hole pockets $0.4m_0$ (α: $m_{\mathrm{eff}} \sim 0.15m_0$, β: $m_{\mathrm{eff}} \sim 0.65m_0$), we obtained a reasonable agreement by $J = 90$ meV between the mobility above and the magnetic Hall mobility ($R_H/(\rho_{xx} - \rho_{\mathrm{ph}} - \rho_0)$ as shown in Fig. S24D. The estimated J is consistent with that extracted from DFT.

9. Estimation of mean free path of various multi-*q*-hosting materials.

In diffraction experiments, the magnetic modulation is observed as the modulation wavenumber q_{mag} in reciprocal space, which is converted into the magnetic modulation wavelength λ_{mag} by $\lambda_{\mathrm{mag}} = 2\pi/q_{\mathrm{mag}}$. In the form of the triangular lattice, λ_{mag} is related with the magnetic superlattice constant a_{spin} by $\lambda_{\mathrm{mag}} = \sqrt{3}/2a_{\mathrm{spin}}$. The double-$q$ state including in $GdRu_2Si_2$ and the ICM2 state in $EuAg_4Sb_2$, on the other hand, $a_{\mathrm{spin}} = 2\pi/q_{\mathrm{mag}}$ We have collected values of a_{spin} for a range of multi-q-hosting systems as summarized in Table S3 and used in constructing Fig. 4G. It is of interest to compare this spin length scale to that of the conduction electrons. As noted in the main text, for the case of $EuAg_4Sb_2$, we observed quantum oscillations and can therefore construct the quantum mean free path $l_q = v_F\tau_q$, v_F and τ_q are the Fermi velocity and the quantum scattering time for the α pocket, respectively (this exceeds a_{spin}, see Table S3). Using information about the Fermi surface, from the Boltzmann transport theory it is also possible to construct a transport mean free path via $l_{\mathrm{mfp}} = 12\pi^3\hbar/(e^2\rho S_F)$, where ρ is the resistivity and S_F is the Fermi surface area [31]. If information about the Fermi surface from quantum oscillations or electronic structure calculations is available these can determine S_F; this is often challenging in multi-q-hosting material systems and conventionally an approximation using carrier density n derived from the Hall constant $|R_H| = 1/ne$ is used to determine $S_F = 4\pi(3\pi^2 n)^{2/3}$ [29, 31]. We construct Fig. 4G using this convention for Gd_2PdSi_3, $Gd_3Ru_4Al_{12}$, $GdRu_2Si_2$, $Fe_{1-x}Co_xSi$, FeGe, MnGe, EuPtSi, $Mn_{1.5}PtSn$ alloys, MnNiGa, $GdNi_2B_2C$, $EuAl_4$, $CeAuSb_2$, and $SrFeO_3$ (see Table S3); for direct comparison we also calculate and include this for $EuAg_4Sb_2$ (a similar result is obtained using a quasi-2D transport approach [87]). For FeNiPdP alloys and CoMnZn alloys only the magnetic modulation is available (these are shown along the horizontal axis of Fig. 4G). For MnSi, l_{mfp} is taken from

literature measurements of the dHvA effect [27,28], estimated to be approximately 6 nm in the skyrmion phase at $T = 28$ K [88].

We note that our estimation of the mean free path of $GdRu_2Si_2$ is comparable with that of $EuAg_4Sb_2$ whereas the Hall conductivity anomaly associated with the multi-q phase formation in $GdRu_2Si_2$ is not so large [19] compared with $EuAg_4Sb_2$. They would be ascribed to the location of the filled 4f level below E_F, ($E_{4f} \sim$ -4 eV for $GdRu_2Si_2$ [89], $E_{4f} \sim$ -1.5 eV for $EuAg_4Sb_2$ (Fig. S2)) dimensionality of the Fermi surfaces [90], and the difference of the ratio of charges participating the topological magnetotransport to the whole carrier density ($n \sim 10^{22}$ cm^{-3} for $GdRu_2Si_2$, $n \sim 10^{20}$ cm^{-3} for $EuAg_4Sb_2$). These salient distinctions would be ascribed to the stacking of quasi-2D anionic Ag_2Sb layers with magnetic Eu^{2+} lattices, which provides useful insights to future material designs.

10. *k·p* model and interaction between a carrier pocket and spin moiré superlattice.

In this section, we discuss the effective $k{\cdot}p$ Hamiltonian for the α pocket under the nesting effects of the candidate spin moiré superlattice states for the ICM2 phase (depicted in Figs. 4B-D). We assume a quasi-2D electrons gas with the effective mass m^*, as supported by the energy dispersions observed:

$$H(k_x, k_y) = E_0 - \frac{\hbar^2 \vec{k}^2}{2m^*}. \tag{S12}$$

We performed numerical calculations in a model with low-energy description given by the above dispersion considering the coupling to underlying spin superstructure. From this, we obtain the band structure, Chern numbers, density of states (Fig. 4H).

As our starting point, we consider the triangular lattice double-exchange model

$$H_0 = -t \sum_{<r\, r'>} (c^\dagger_{r\sigma} c_{r'\sigma} + h.c.) - \sum_r J c^\dagger_{r\alpha} \left(\vec{s}_r \cdot 1/2 \vec{\sigma}_{\alpha\beta}\right) c_{r\beta} \tag{S13}$$

where $t = (3m^* a_0^2)^{-1}$ is the nearest-neighbor hopping on the triangular lattice with lattice constant a_0, J is the ferromagnetic spin exchange coupling, and $\vec{s}_r$ is the local Eu moment for the vector field describing a spin-moiré texture with the lattice constant a_{spin}. Strictly speaking, H_0 at low densities describes electrons in a parabolic band bottom subject to J, while the α pocket is hole-like. However, minimizing H_0 with an electron filling f is equivalent to

minimizing -H_0 (which has a parabolic band top) with a hole filling f because the sum of single-particle energies is constant (in fact, zero) independent of $\vec{s}_r$. In the following, we use the language of minimizing H_0 with an electron filling f to emphasize that no role is played by the higher energy bands.

The filling is $f \approx 2$ carriers per moiré unit cell. At low filling, only the parabolic dispersion of the band edge is important. We also include a Zeeman field B:

$$H = H_0 + Bgs_r^z. \tag{S14}$$

For a density $x = k_F^2/2\pi$ the carriers induce the well-known RKKY interaction between magnetic moments which strongly favors a periodic modulation at wavevector $2k_F$. Indeed, the observation that $2k_F$ is commensurate with the moiré BZ diameter points strongly in favor of an RKKY-organized spin moiré superlattice in $EuAg_4Sb_2$, that reconstructs the electronic band structure in the moiré BZ. When intrinsic spin-spin interactions are small, the total electronic energy dictates the ground state spin texture. We note that additional terms such as four-spin interactions [72,91] and uniaxial/trigonal/hexagonal single-site anisotropies [69,70] may be key to engineering a variety of SMS.

In Ref. [92] a variational calculation reveals that the ferromagnetic state is unstable to wavevector $2k_F$ ordering below a critical value of $J/E_F \approx 1.8$. This is calculated from the slope of the phase boundary in Fig. 1b of Ref. [92] at low densities. Below the critical value of J/E_F, spin moire superlattice states can be self-consistently stabilized by their reconstructed electronic bands, with $2k_F$-ordering due to the RKKY interaction. We refer to the condition $J \approx E_F$ as the “resonant condition”. The condition $J \approx E_F$ is crucial in generating electronic bands with suppressed dispersion as only in this regime will the exchange gap balance the dispersion across the Brillouin zone (see Fig. 4F) [5,46,47]. This matching thus undergirds both the magnetic and electronic states observed and is crucial for the observed phenomena. In the strong coupling limit, $J >> E_F$, the self-consistent stable state is ferromagnetic. We also note that the J/E_F for the β band and SS are 0.2 and 0.1, respectively (see Figs. S3A and S4E) even though J is expected to be comparable to 100 meV (see Fig. S24B). These off-resonant condition suggests that these bands do not critically contribute to the formation of SMS, while the effect of periodic modulation to the β band carriers is expected to be relevant (as J itself is non-negligible). We hypothesize that the hole-like remnant Hall conductivity in the ICM2 (Fig. 3C) derives from imperfect quenching of the β band.

For Fig. 4H, we used a two-dimensional tight-binding model of a triangular lattice (as the Eu sublattice for each layer) with $t = 0.75$ eV from $m^*/m_0 = 0.15$ and $a_0 = 4.79$ Å. Consistent with the DFT results at the Γ pocket we took $J = 0.12$ eV, implying $J/E_F \sim 0.93$ since $E_F = 0.129$ eV. The magnetic superlattice is taken to be the commensurate super unit cell on top of the triangular lattice as shown in Fig. S25A in the real space and the reciprocal lattice space, which is close to the observation $1/q_{ICM2} = 11.4$ and orientation with respect to the crystal axes. The mutual angle (Φ) between the q_1 and q_2 are close to 90°, which is slightly larger than the experimental results ($\Phi \approx 84°$, see Sec. S5). The value of t is also rescaled to maintain the carrier density, allowing for a tunable atomic unit cell size. Due to the near orthogonal q vectors, q_1 and q_2, the magnetic BZ becomes close to a square (Fig. S25B). The spin configurations were formed from a double-q ansatz as given in Eq. (S7). Each multi-q state was obtained by taking $S_{z,q} = 1$, $h = 1$, and $S_{z,0} = -0.3$ (SkL, Fig. 4B), $S_{z,q} = 1$, $h = -1$ and $S_{z,0} = -0.3$ (aSkL, Fig. 4C), and $S_{z,q} = 0$, and $S_{z,0} = -0.3$ (VL, Fig. 4D) They produce qualitatively similar band foldings (Fig. 4H).

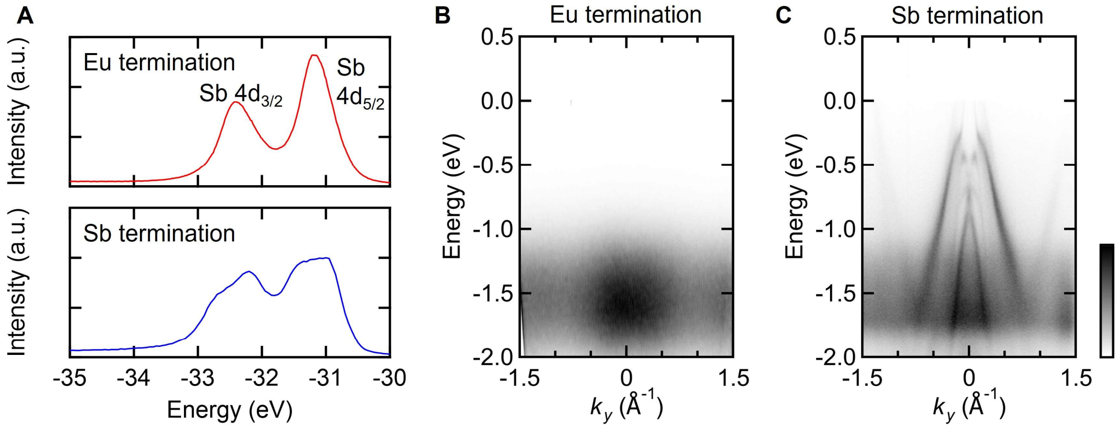

Fig. S1|Termination dependence of XPS spectrum for $EuAg_4Sb_2$.

A Two representative XPS spectra of in situ cleaved $EuAg_4Sb_2$, from which we identified the surface termination as Eu (red curve) and Sb (blue curve) layers. **B** and **C** Band dispersion along Γ-M line measured on Eu and Sb terminations, respectively. The color bar at the bottom right indicates intensity from minimum (bottom) to maximum (top).

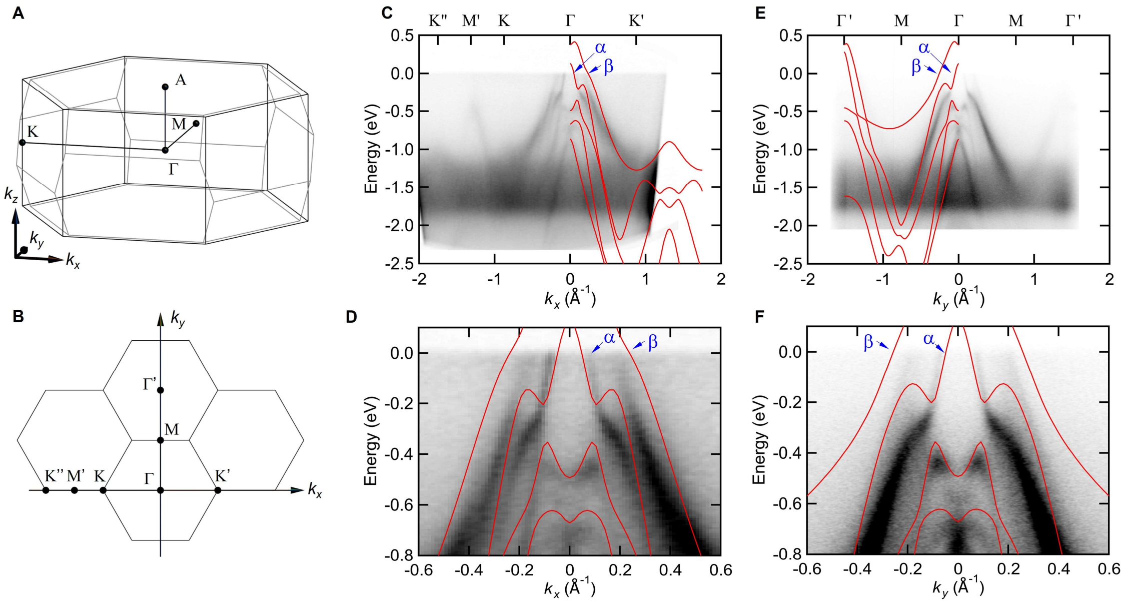

Fig. S2|Band dispersion in ARPES measurements and DFT calculations for $EuAg_4Sb_2$.

A Relationship between rhombohedral (gray lines) and hexagonal (black lines) Brillouin zone (BZ), and definition of symmetry points. **B** Symmetry points in k_z = 0 plane for the hexagonal BZ notation. Note that Γ', M', and K'' are distinct from the Γ, M, or K' points in the rhombohedral BZ notation. **C-F** ARPES results for the band dispersion **C-D** in the k_x direction (K-Γ-K' cut), and **E-F** in the k_y direction (M-Γ-M cut). The k_x and k_y axes are defined in Fig. S2B. Corresponding DFT calculations are superimposed as a comparison. The Fermi energy for the DFT calculations is shifted by -100 meV.

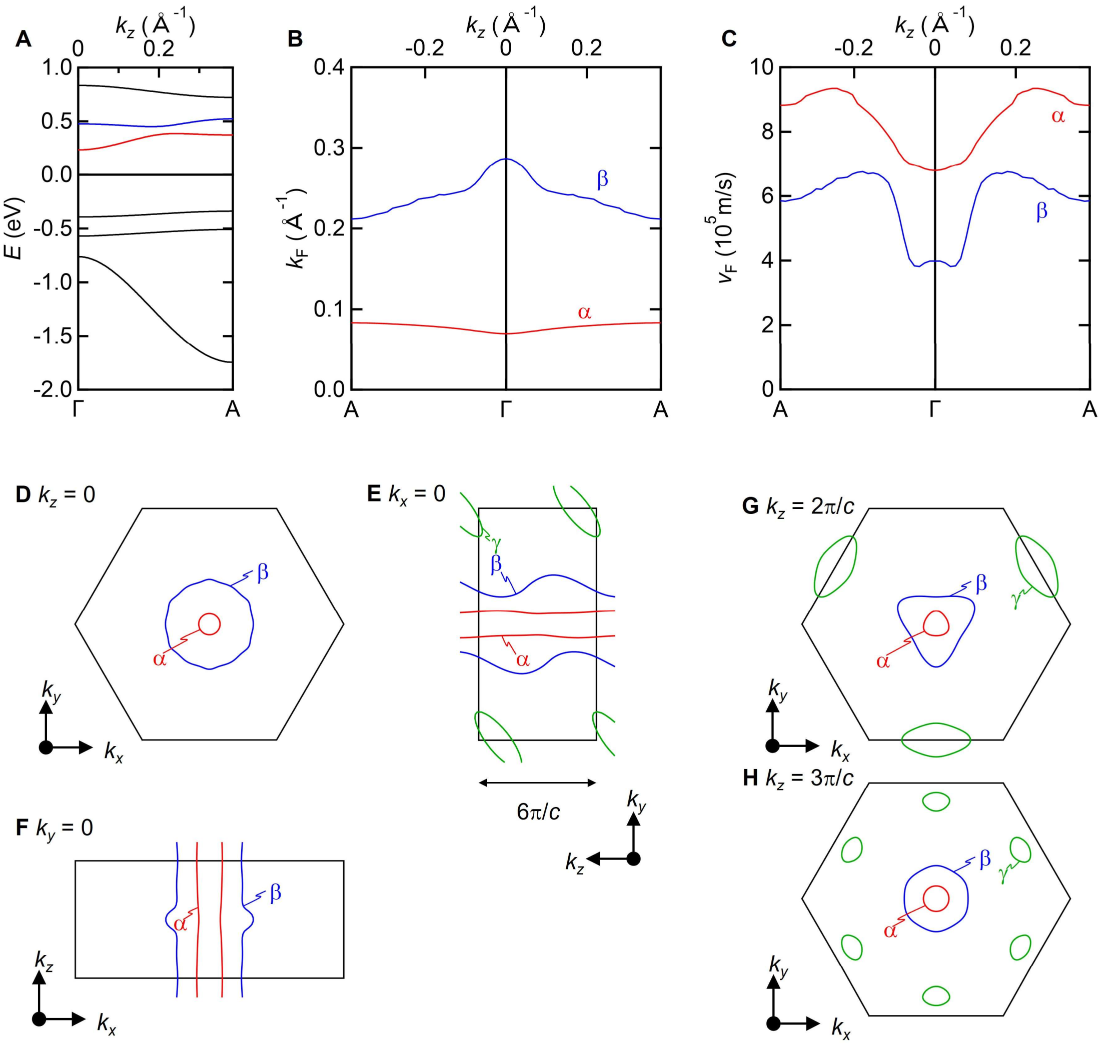

Fig. S3|The k_z dependence of Fermi surface properties of $EuAg_4Sb_2$.

A Energy dispersion of each band around E_F in the Γ-A cut. The red (blue) line denotes the α (β) band. **B-C** The k_z dependence of the averaged **B** Fermi surface radius (k_F) and **C** Fermi velocity (v_F) for α (red) and β (blue) pockets (see the text in Sec. S1 for the definitions of k_F and v_F). **D-H** Cross sections of the Fermi surfaces at each plane defined by **D** $k_z = 0$, **E** $k_x = 0$, **F** $k_y = 0$, **G** $k_z = 2\pi/c$, and **H** $k_z = 3\pi/c$.

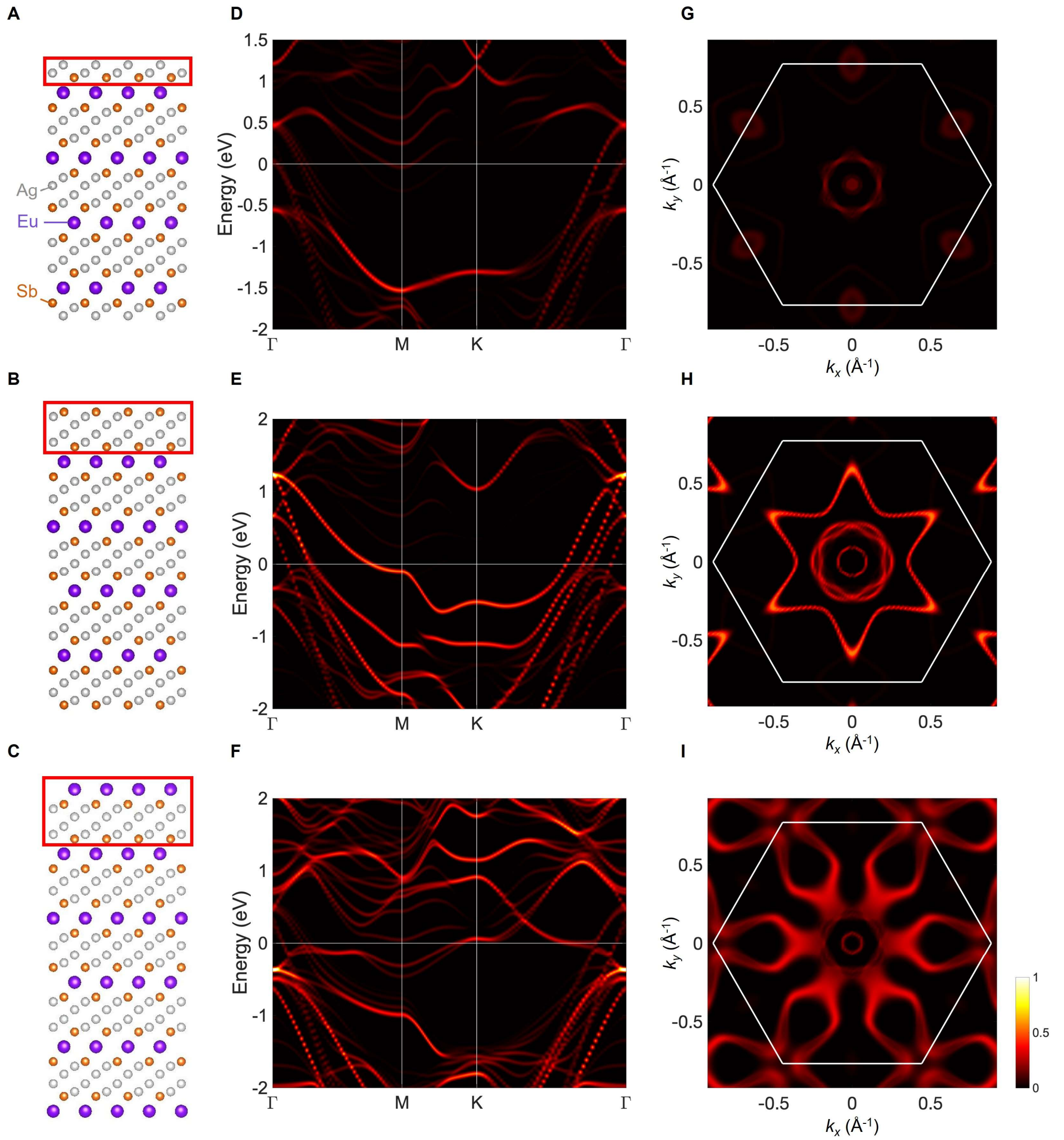

Fig. S4| $EuAg_4Sb_2$ thin film slabs and surface spectral functions.

A-C Three types of $EuAg_4Sb_2$ thin film slabs with Ag_2Sb, Ag_4Sb_2, Eu terminations, respectively. **D-F** The electronic band structures for these slabs weighted by surface wave function projections, defined by the regions highlighted by the red box in **A-C**. **G-I** The surface spectral function in the momentum space at Fermi level of these different thin film slab types. From the simulations, the electronic structure of $Eu_4Ag_{20}Sb_{10}$ slab (middle) with Ag_4Sb_2 terminations resembles the surface features identified in the ARPES.

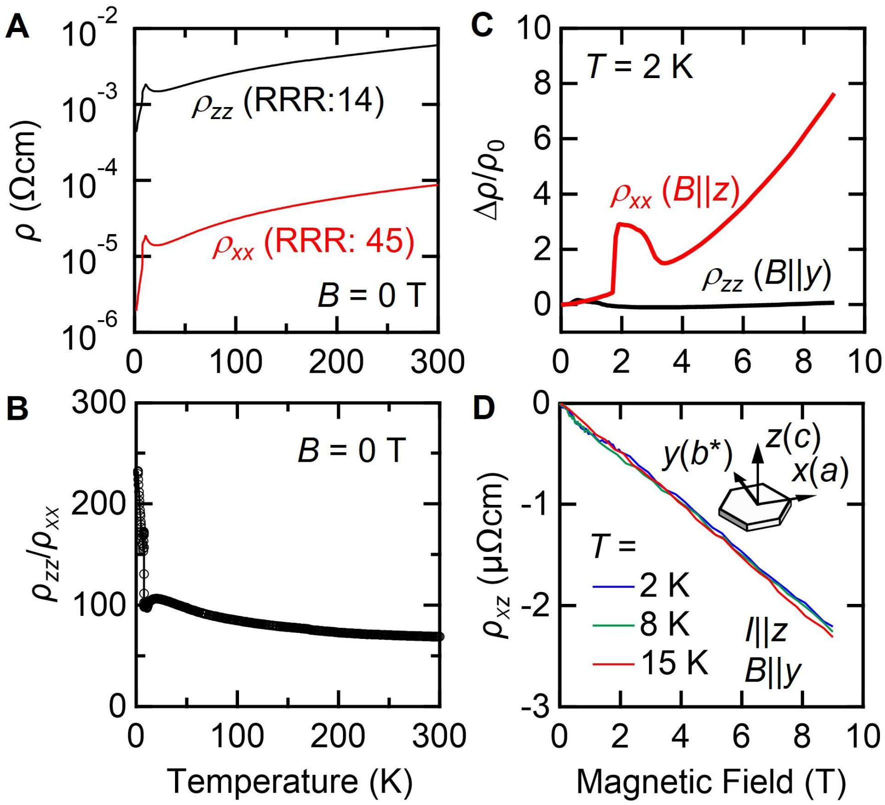

Fig. S5|Out-of-plane transport properties of $EuAg_4Sb_2$.

A-B Temperature dependence of (**A**) the in-plane (ρ_{xx}) and the out-of-plane (ρ_{zz}) resistivity, and (**B**) resistivity ratio (ρ_{zz}/ρ_{xx}) in zero field. **C** Transverse magnetoresistance ($\Delta\rho/\rho_0 = [\rho(B) - \rho(B = 0)]/\ \rho(B = 0)$) at $T = 2$ K. **D** Hall resistivity (ρ_{xz}) with the current $I \| z$ and $B \| y$ at different temperatures. The inset defines the cartesian coordinates, x, y, z, in terms of the crystal axes, a, b^*, c.

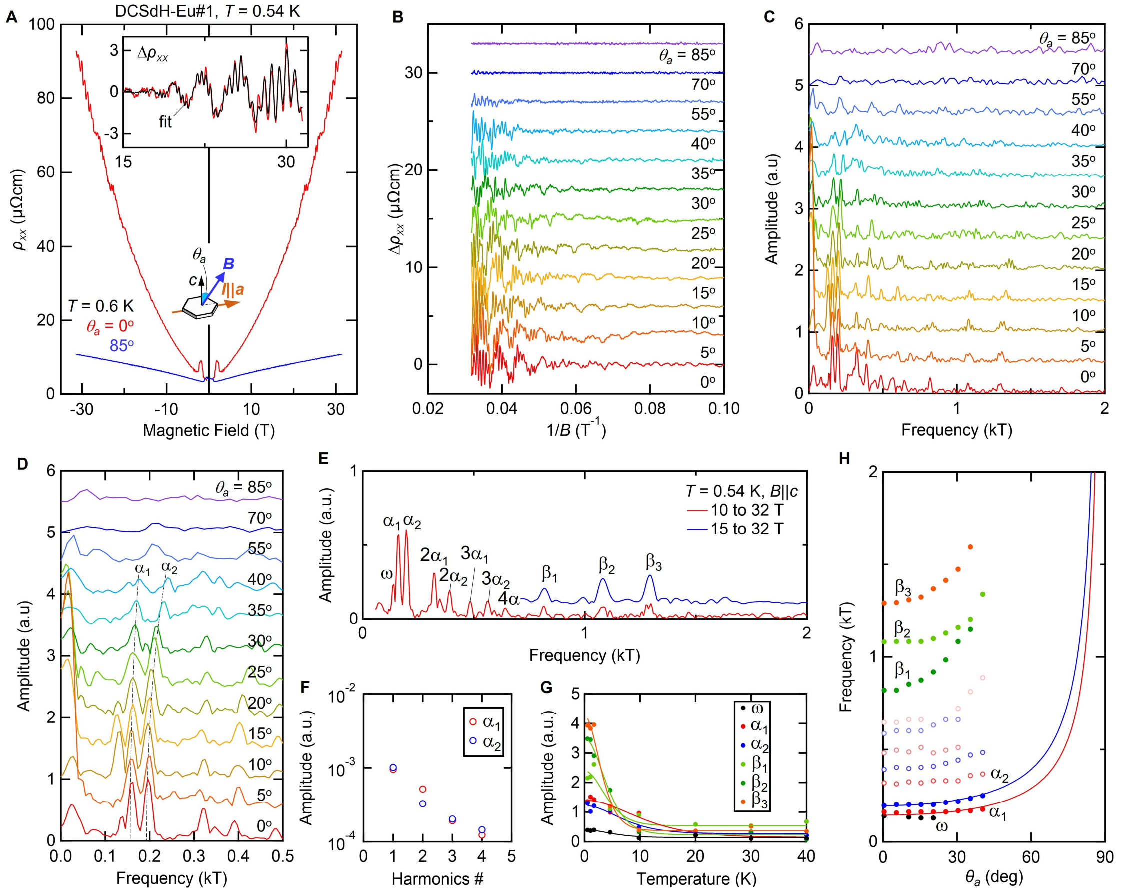

Fig. S6|Shubnikov-de Haas oscillations in $EuAg_4Sb_2$.

A External magnetic induction (B) dependence of the in-plane resistivity (ρ_{xx}) for B along the c axis (red) and close to the current (I) direction (blue). DCSdH-Eu#1 is the sample indicator. θ_a is the angle between B and c axis. The inset magnifies the quantum oscillations of resistivity (red curve) and a fitting curve (black curve, see Sec. S3). A smooth background is subtracted. **B** Inverse field (B^{-1}) dependence of the background-subtracted resistivity ($\Delta\rho_{xx}$) measured at T = 0.54 K. **C** FFT of the data in **B**. **D** Magnified view of **C** around low-frequency region. Dashed lines are guide to the eyes for α_1 and α_2 branches. Each data with different θ_a appearing in through **B**-**D** are shifted upwards for clarity. **E** FFT of SdH oscillations at T = 0.54 K with $B\|c$ analyzed with different B windows. Red: B = 10 to 32 T; blue: B = 15 to 32 T. The latter is shifted upward for clarity. Positions of the branches α_1-β_3 and ω are assigned. $n\alpha_1$ and $n\alpha_2$ (n = 2, 3, 4) are higher harmonics of α_1 and α_2, respectively. **F** Frequency peak-amplitude vs. harmonics number (n) for α_1 and α_2. **G** Temperature dependence of peak amplitude (closed circles) and fits with LK formula (solid lines) for each branch following a clear angular evolution. **H** Angular (θ_a) dependence of frequency for each branch (closed circles). Open circles are for the higher harmonics (red: $n\alpha_1$; blue: $n\alpha_2$). Solid lines are fits with the model of a cylindrical Fermi surface.

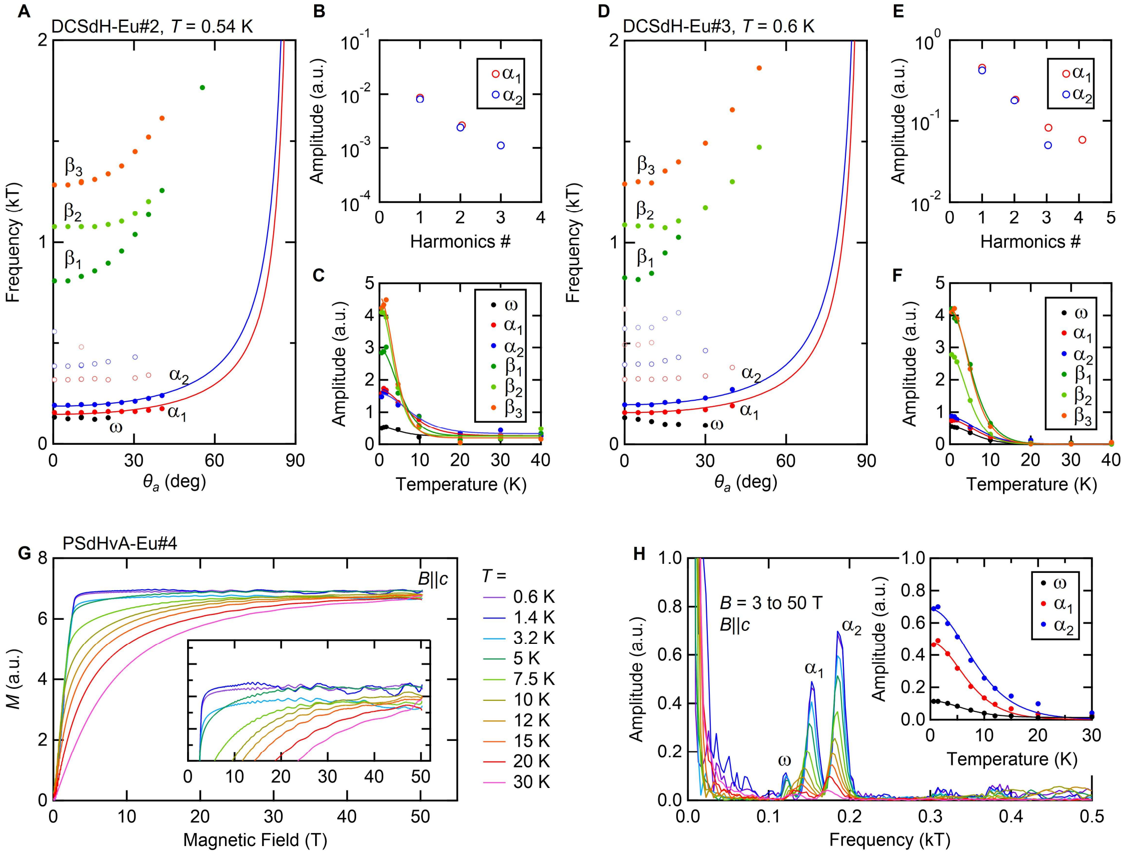

Fig. S7|SdH and dHvA oscillations in $EuAg_4Sb_2$.

A-F SdH oscillations data set for samples (**A-C**) DCSdH-Eu#2 and (**D-F**) DCSdH-Eu#3. Definitions of the symbols are the same as in Fig. S6. **G** Pulse-field magnetization (*M*) data for $B||c$ at various temperatures obtained with the sample PsdHvA-Eu#4. The inset is the magnified view around the *M*-saturation region. **H** FFT of the dHvA oscillations between $B = 3$ and 50 T, where a smooth background was subtracted from the raw data. The inset shows the temperature dependence of peak amplitude (closed circles) for the observed branches (ω, α_1, α_2), and fit with the LK formula as solid lines.

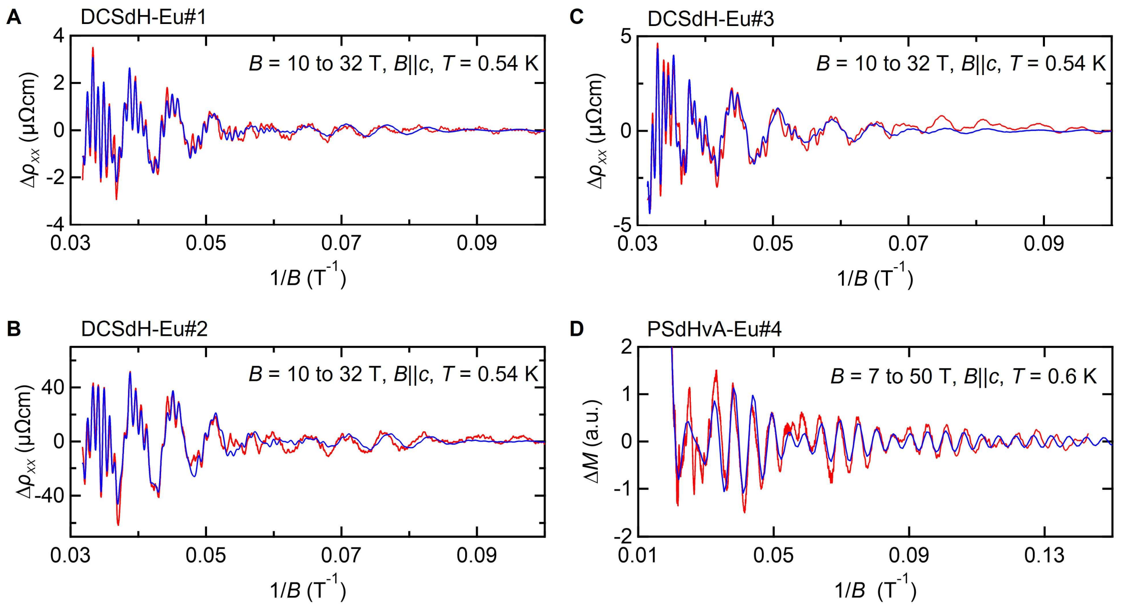

Fig. S8|Fitting of the SdH and dHvA oscillations of $EuAg_4Sb_2$.

A-D B^{-1} dependence of (**A-C**) SdH and (**D**) dHvA oscillations (shown in red) and a fit (blue curves), respectively (see Sec. S3).

Table S1|Fermi surface parameters of $EuAg_4Sb_2$ estimated by SdH, dHvA, ARPES measurements, and DFT Calculations.

m_{eff}: effective mass; m_0: free electron mass; f: oscillation frequency; k_F: Fermi wave number; v_F: Fermi velocity; τ_q: quantum lifetime; μ_q: quantum mobility; l_q: quantum mean free path. In each panel, parameters on the upper half are estimated from the temperature dependence of oscillation amplitude obtained by FFT (Figs. S6-S7), and those on the lower half are done by the fitting of the raw data (Fig. S8). For ARPES and DFT, they are obtained from Figs. S2, and S3, respectively.

DCSdH-Eu#1	ω	α_1	α_2	β_1	β_2	β_3
Temperature dependence of FFT						
m_{eff}/m_0	0.434	0.122	0.177	0.734	0.722	0.948
f (T)	139.8	160.5	196.2	822.0	1079	1297
k_F (Å^{-1})	0.0652	0.0698	0.0772	0.158	0.181	0.199
v_F (m/s)	1.7×10^5	6.6×10^5	5.1×10^5	2.5×10^5	2.9×10^5	2.4×10^5
Fit raw data						
m_{eff}/m_0	0.254	0.122	0.180	0.427	0.320	0.430
freq. (T)	138.8	159.0	189.5	810.0	1085	1286
τ_q (ps)	0.075	0.058	0.040	0.067	0.052	0.066
μ_q (cm^2/Vs)	1577	1209	843	1409	1079	1385
l_q (nm)	22	38	20	29	34	35

DCSdH-Eu#3	ω	α_1	α_2	β_1	β_2	β_3
Temperature dependence of FFT						
m_{eff}/m_0	0.196	0.180	0.169	0.526	0.615	0.564
f (T)	118.8	160.3	196.0	831.3	1081	1289
k_F (Å^{-1})	0.0601	0.0698	0.0772	0.159	0.181	0.198
v_F (m/s)	3.5×10^5	4.5×10^5	5.3×10^5	3.5×10^5	3.4×10^5	4.1×10^5
Fit raw data						
m_{eff}/m_0	0.217	0.167	0.168	0.610	0.655	0.693
freq. (T)	132.3	160.9	191.8	833.5	1084	1293
τ_q (ps)	0.045	0.036	0.089	0.097	0.12	0.086
μ_q (cm^2/Vs)	365	756	1861	2034	2429	1789
l_q (nm)	15	18	47	29	37	28

DCSdH-Eu#2	ω	α_1	α_2	β_1	β_2	β_3
Temperature dependence of FFT						
m_{eff}/m_0	0.250	0.168	0.154	0.581	0.776	0.742
f (T)	130.8	154.6	190.2	802.6	1089	1290
k_F (Å^{-1})	0.0630	0.0685	0.0760	0.156	0.182	0.198
v_F (m/s)	2.9×10^5	4.7×10^5	5.7×10^5	3.1×10^5	2.7×10^5	3.1×10^5
Fit raw data						
m_{eff}/m_0	0.249	0.168	0.153	0.477	0.925	0.726
freq. (T)	140.0	157.1	188.9	805.2	1083	1278
τ_q (ps)	0.093	0.090	0.062	0.15	0.19	0.13
μ_q (cm^2/Vs)	1953	1876	1289	3149	4054	2674
l_q (nm)	28	43	35	57	44	40

PSdHvA-Eu#4	ω	α_1	α_2	β_1	β_2	β_3
Temperature dependence of FFT						
m_{eff}/m_0	0.126	0.179	0.148			
f (T)	121.2	154.0	185.0			
k_F (Å^{-1})	0.0607	0.0684	0.0750			
v_F (m/s)	5.6×10^5	4.4×10^5	5.9×10^5			
Fit raw data						
m_{eff}/m_0	0.0841	0.102	0.046			
freq. (T)	114	159.9	193.0			
τ_q (ps)	0.005	0.051	0.073			
μ_q (cm^2/Vs)	105	876	2823			
l_q (nm)	4.1	40	142			

ARPES	α	β
k_F (Å^{-1})	0.076(10)	0.166(25)
v_F (m/s)	9.2×10^5	5×10^5

DFT	α_1	α_2	β_1	β_2
k_F (Å^{-1})	0.070	0.083	0.212	0.287
v_F (m/s)	6.80×10^5	8.82×10^5	5.85×10^5	3.98×10^5

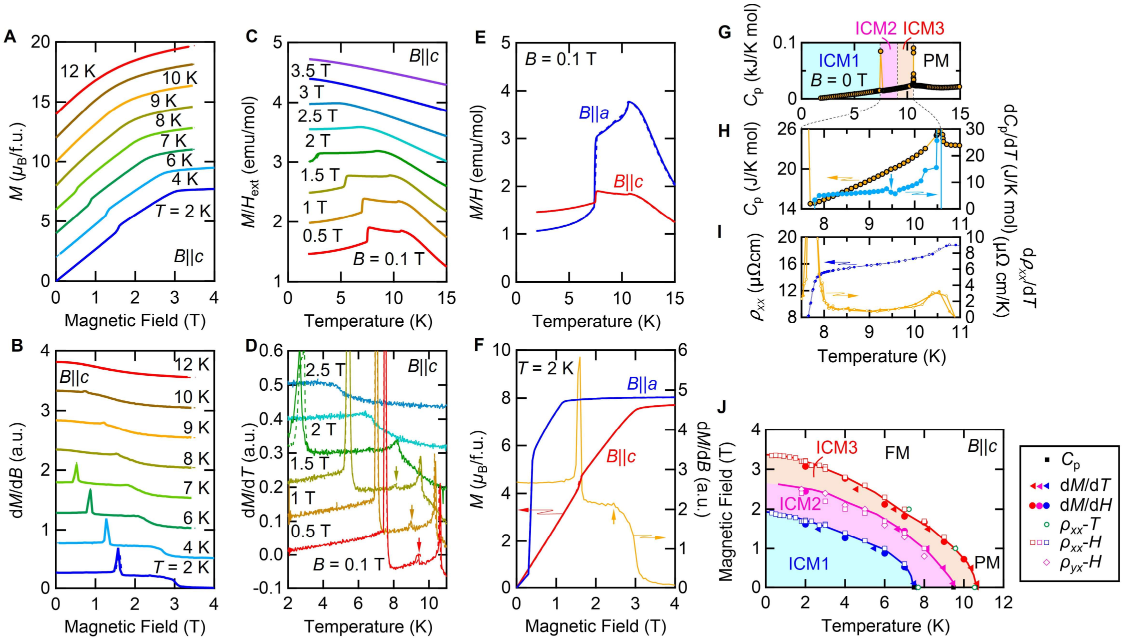

Fig. S9|Magnetizatic properties and magnetic phase diagrams of $EuAg_4Sb_2$.

A-B Magnetic field dependence of (**A**) magnetization (M) and (**B**) field-derivative (dM/dB) for $B \| c$ measured at various temperatures ($B = \mu_0 H_{ext}$, where H_{ext} is the applied magnetic field). **C-D** Temperature dependence of (**C**) magnetic susceptibility (M/H_{ext}) and (**D**) temperature-derivative (dM/dT) for $B \| c$ measured at various magnetic fields. Data in **A-D** are shifted for clarity. Solid (dashed) lines in **A-D** are for a field/temperature decreasing (increasing) process. **E** Temperature dependence of M/H_{ext} for $B \| c$ (red curve) and $B \| a$ (blue curve) at $B = 0.1$ T. **F** Corresponding magnetic field dependence of M at $T = 2$ K. dM/dB for $B \| c$ is also shown. **G** Temperature dependence of the specific heat (C_p). **H** Zoom-in of C_p and temperature-derivative (dC_p/dT) near the transition temperature between ICM2 and ICM3 (represented by cyan arrow). **I** Corresponding curves for the in-plane resistivity. **J** The magnetic phase diagram for $B \| c$ determined by specific heat, magnetization, resistivity, and Hall resistivity measurements. ICM1, ICM2, ICM3: incommensurate magnetically modulated phases; PM and FM: paramagnetic and field-induced ferromagnetic regions, respectively.

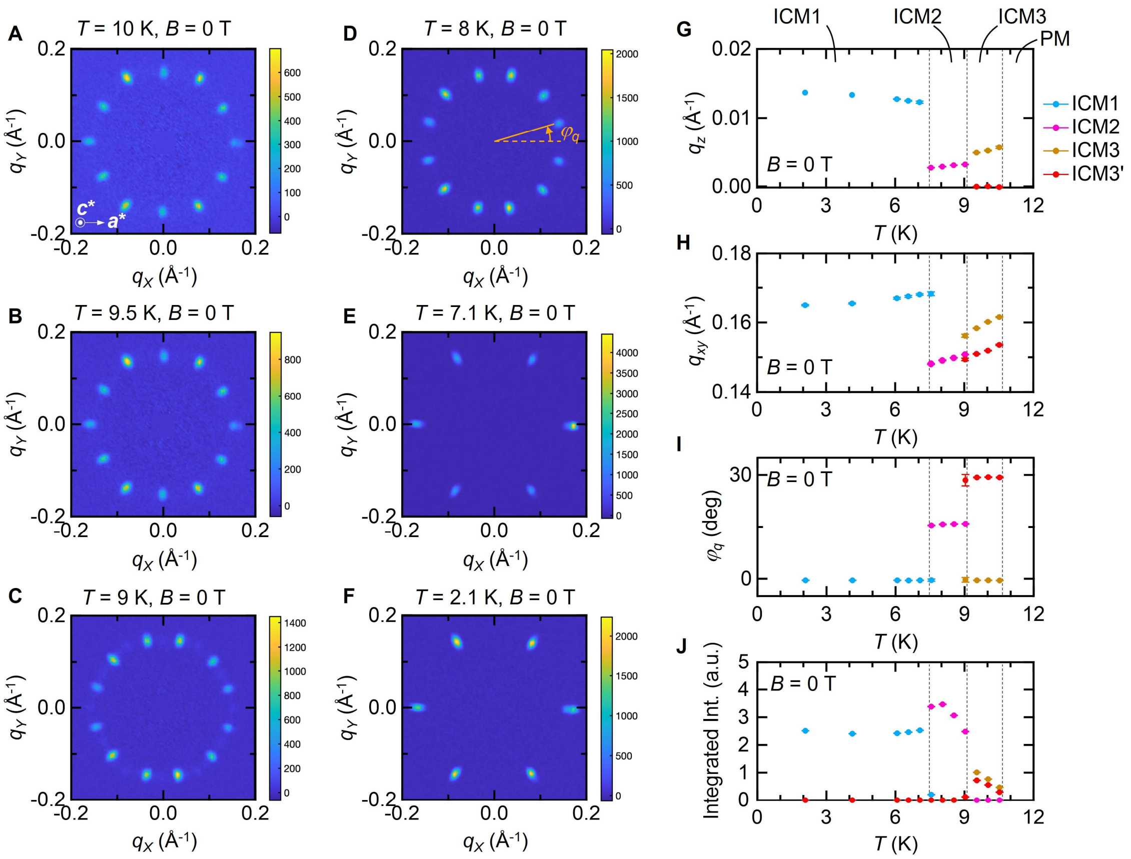

Fig. S10| Temperature dependence of magnetic SANS pattern of $EuAg_4Sb_2$ in zero field.

A-F Thermal evolution of the SANS patterns in zero field. The neutron beam is directed near parallel to the *c* axis, and the intensity was integrated in rocking-scans. **G-J** Temperature dependence of q_z, in-plane q (q_{xy}), azimuthal angle for the q vectors (φ_q), and integrated intensity. φ_q is measured from one of the high-symmetry directions nearby (see **D**). Error bar is standard deviation. See inset of Fig. 3A for legend.

fffff

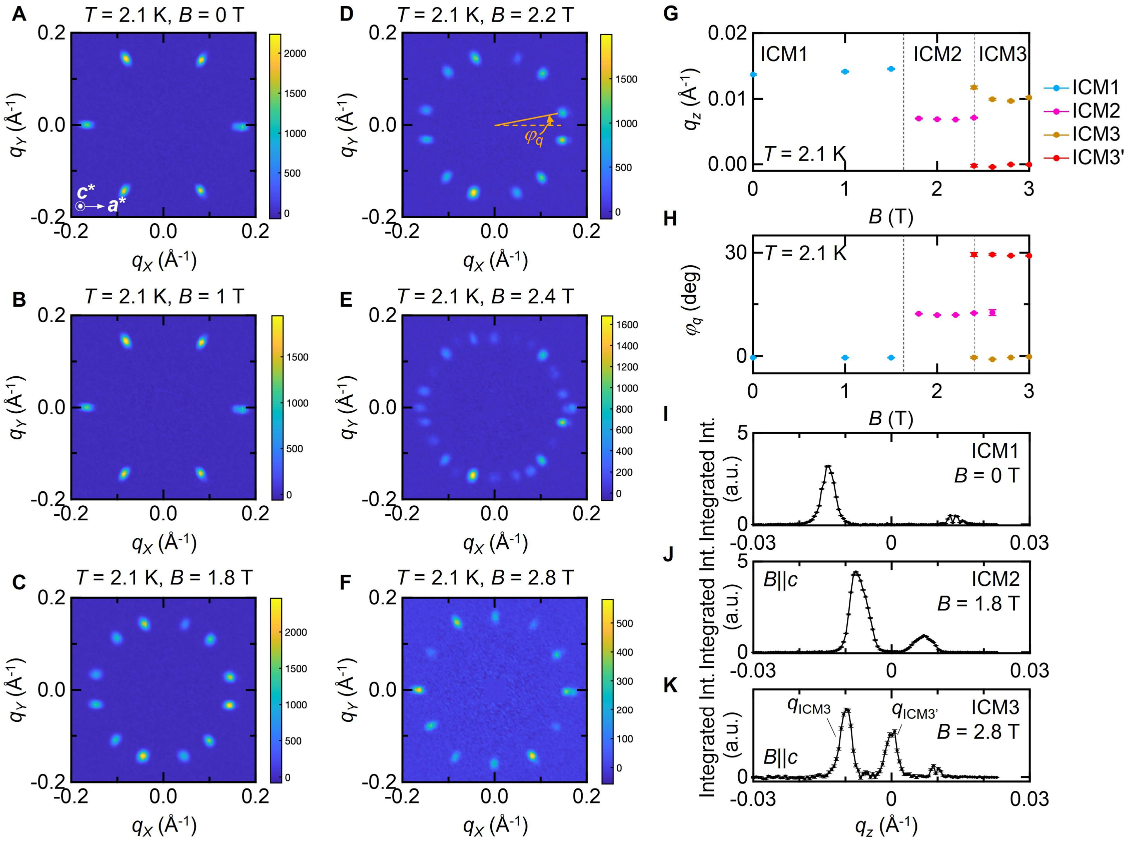

Fig. S11| Field dependence of magnetic SANS pattern of $EuAg_4Sb_2$ at $T = 2.1$ K.

A-F Field-induced evolution of the SANS patterns for $B \| c$. The neutron beam is directed near parallel to the c axis, and the intensity was integrated in rocking-scans. **G-H** Field dependence of **G** q_z, and **H** φ_q. **I-K** q_z dependence of magnetic Bragg peaks for each phase. Error bar is standard deviation. See inset of Fig. 3A for legend.

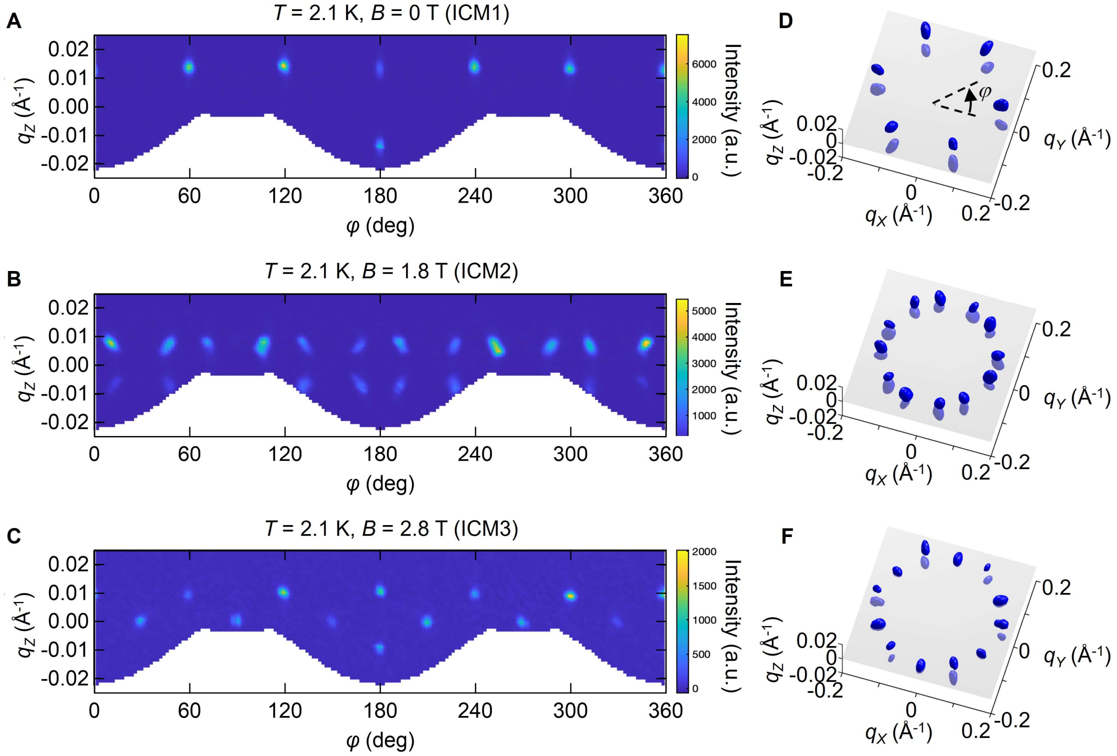

Fig. S12| The 3D visualization of the magnetic Bragg spots.

A-C Azimuthal angle (φ) dependence of the intensity of the magnetic Bragg scattering for ICM1, ICM2, and ICM3 states. **D-F** 3D visualization of the Bragg peaks. Intensities are symmetrized with respect to $\pm\boldsymbol{q}$. Gray square is the plane at $q_z = 0$.

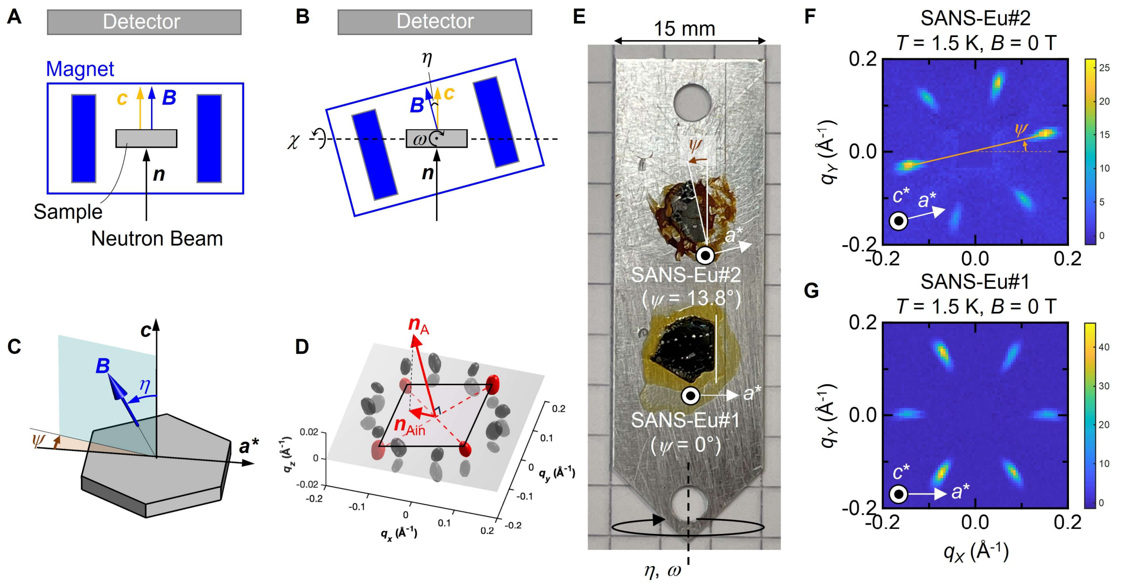

Fig. S13| Experimental setup for the tilt-field SANS experiment.

A-B Schematic geometry of SANS experiment with **A** $\eta = 0°$ and **B** $\eta > 0°$, where η is the tilt angle of the magnet from the sample c axis. The magnet is indicated in blue, sample in gray, detector in gray, and neutron beam in black. ω and χ are the axes of rotation fixed on the sample. In the rocking scan, sample and magnet are moved together so that the tilt angle η with respect to the c axis is unchanged. **C** Schematic geometric configuration between crystal axes and tilted magnetic field for SANS-Eu#2. Field-rotation plane (cyan plane) is rotated away from the c^*a^*-plane by ψ in order to lift the degeneracy of double-q domains. **D** Schematic illustration for the definition of $\boldsymbol{n}_{\mathrm{A}}$ and $\boldsymbol{n}_{\mathrm{Ain}}$. $\boldsymbol{n}_{\mathrm{A}}$ is the vector normal to the plane spanned by the q vectors (red dots) belonging to a double-q domain A for the ICM2 state. n_{Ain} is the $q_x q_y$-component of $\boldsymbol{n}_{\mathrm{A}}$. **E** Image of crystals used for SANS. SANS-Eu#1 (bottom) is fixed with the a^* axis in the horizontal plane, while SANS-Eu#2 (top) is rotated by $\psi = 13.8°$. **F-G** SANS pattern of each sample in the ICM1 state as a check of the sample orientation.

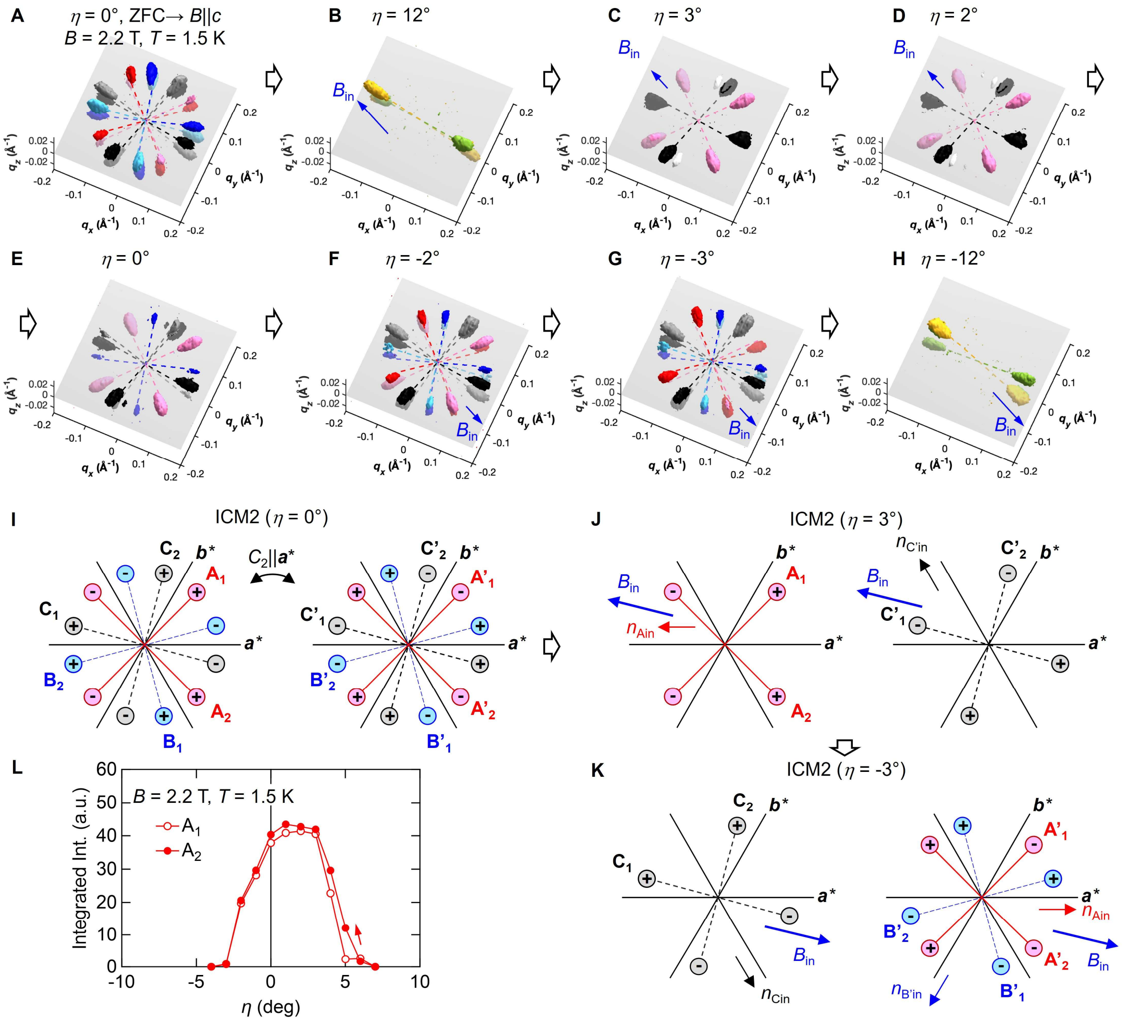

Fig. S14|Selection of multi-q domain by the tilt field.

A-E 3D visualization of the magnetic Bragg spots for the magnetic field tilting away from $B \| c$ ($\eta = 0°$) in the ICM2 phase at $B = 2.2$ T and $T = 1.5$ K to (**B**) $\eta = 12°$ (for a single-q state) and back to (**E**) $\eta = 0°$. Intensities are symmetrized with respect to $\pm\boldsymbol{q}$. **F-H** is the followed opposite tilting to (**H**) $\eta = -12°$. Magnetic diffraction peaks are color-coded based on the assignment of the double-q domains. Pink and black are the two ICM2 domains selected by the tilt. Yellow and green are domains for the in-plane-field-induced single-q state. White are potentially a single-q domain induced by field tilting. Blue arrow is the in-plane orientation of the tilted field (B_{in}). **I-K** Schematic illustration of the domain selection by tilted field. Left and right for each panel correspond to the obverse and reverse twin related by C_2 rotation along the $\boldsymbol{a}^*$ axis. Red, blue, and black dots are Bragg spots for the double-q domains A (composed of the spots A_1 and A_2), B (B_1, B_2), and C (C_1, C_2), respectively. Domain A', B', and C' are related to the A, B, C domains by the C_2 rotation around the a^* axis. Plus and minus signs in each circle denote that of the q_z coordinate. In **J**, the orientation of the B_{in} (blue arrow) is also denoted, which is $\psi = 13.8°$ away from the **-$\boldsymbol{a}^*$** axis (see Fig. S13C). n_{Ain}, $n_{\mathrm{C'in}}$, the $q_x q_y$-component of $\boldsymbol{n}_{\mathrm{A}}$ and $\boldsymbol{n}_{\mathrm{C}}$, are also shown (see Fig. S13D for definition). **L** Tilt-field-angle η dependence of the integrated intensity of peaks A_1 and A_2 belonging to the domain A.

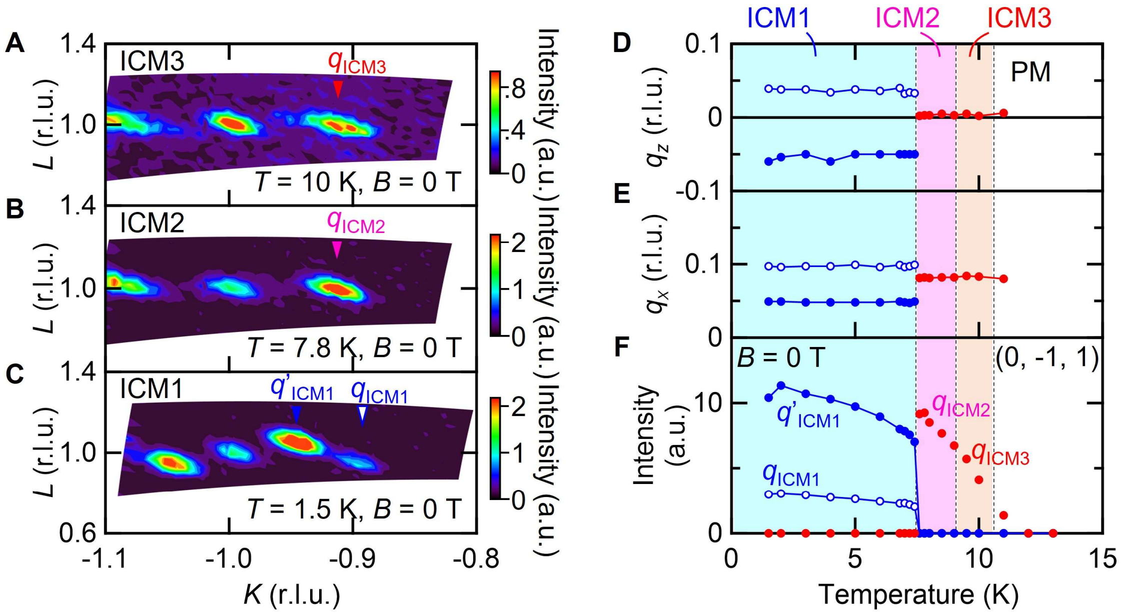

Fig. S15|Magnetic satellite peaks near (0, -1, 1) in neutron diffraction of $EuAg_4Sb_2$.

A-C Magnetic Bragg peaks around (0, -1, 1) in zero field at (**A**) T = 10 K for ICM3 phase, at (**B**) T = 7.8 K for ICM2 phase, and at (**C**) T = 1.5 K for ICM1 state. **D-E** The components of magnetic modulation vector along (**D**) c^* (q_z) and (**E**) $-b^*$ (q_x), respectively, measured around the Bragg point at (0, -1, 1) in zero field. Closed red circles are for the q vector for ICM2/ICM3 (q_{ICM2}, q_{ICM3}), and Open (closed) blue circles are for q_{ICM1} (q'_{ICM1}). **F** Temperature dependence of the integrated intensity for each magnetic Bragg peak.

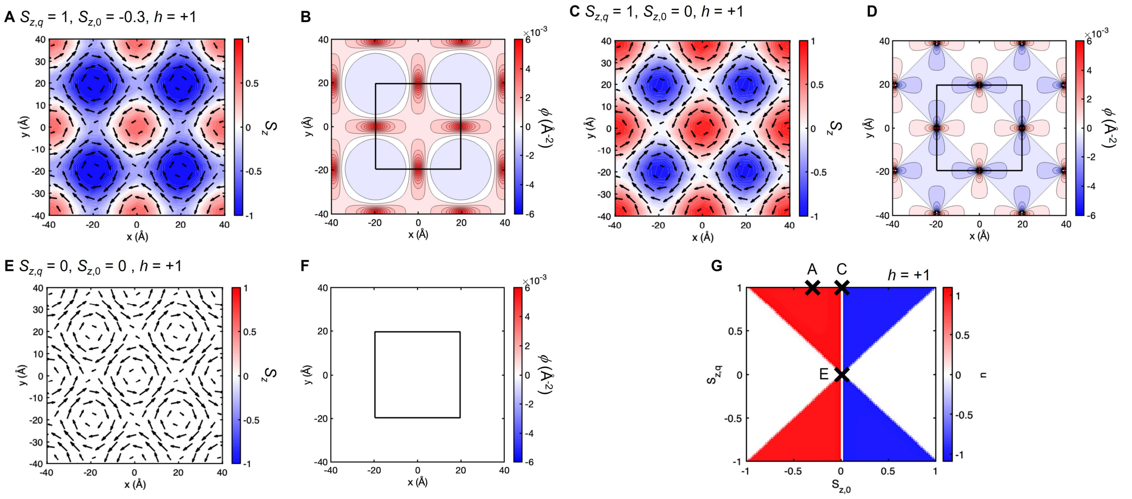

Fig. S16|Candidate spin configurations and skyrmion density.

A Square skyrmion lattice. Black arrows are the in-plane component of spins. The color scale indicates the out-of-plane component of spin (S_z). $S_{z,q}$, $S_{z,0}$, and h are parameters for the ansatz Eq. (S7). **B** skyrmion density ($\phi_{\mathrm{sk}} = \boldsymbol{n} \cdot \left(\frac{\partial \boldsymbol{n}}{\partial x} \times \frac{\partial \boldsymbol{n}}{\partial y}\right)$) of (**A**). **C**-**F** Corresponding figures for (**C**-**D**) meron-antimeron lattice, and (**E**-**F**) vortex lattice. **G** Phase diagram of topological number (n_{sk}: Eq. (S8)) as a function of $S_{0,z}$ and $S_{q,z}$. h is fixed to +1. The corresponding phase diagram for antiskyrmion lattice can be obtained by using h = -1 (therein the n_{sk} = +1 and -1 regions are inter changed).

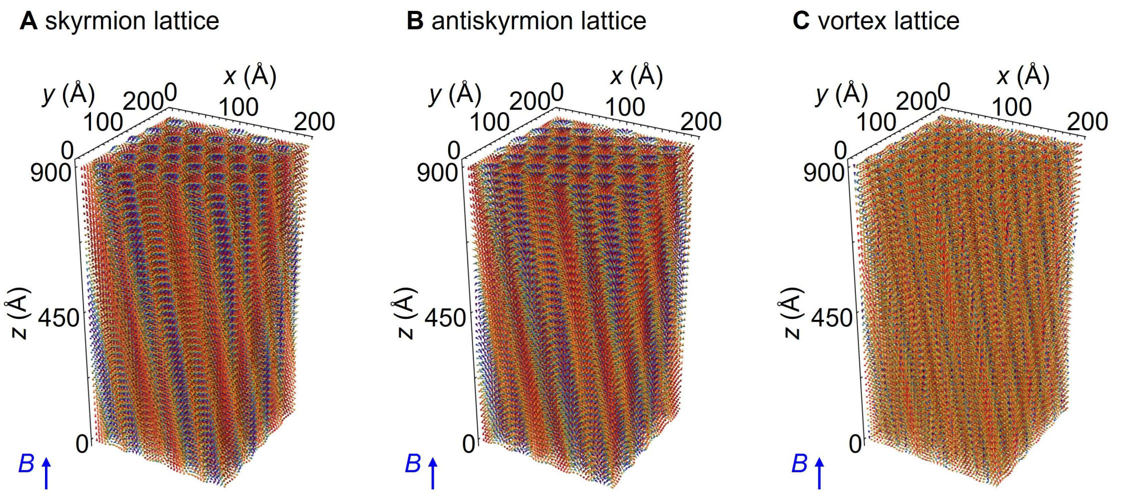

Fig. S17|Candidate spin configurations in three-dimensional space.

Candidate real-space spin configuration of the double-q ICM2 state under a finite magnetic field along the c-axis. Note the vertical z-axis scales are contracted for visibility. **A** Rhombic skyrmion lattice. **B** Rhombic antiskyrmion lattice. **C** Rhombic vortex lattice.

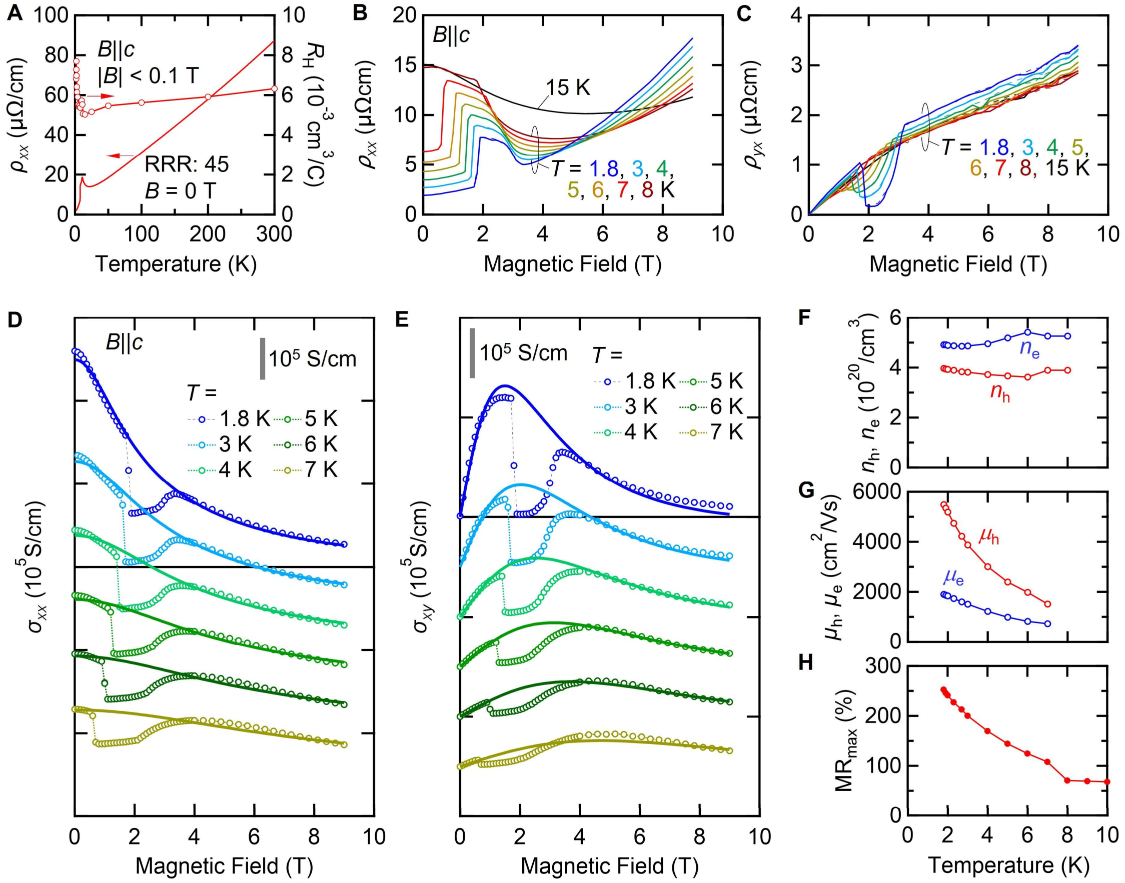

Fig. S18|Magnetotransport properties of $EuAg_4Sb_2$.

A Temperature dependence of the in-plane resistivity (ρ_{xx}) in zero field (left ordinate) and Hall coefficient (R_H) for $B\|c$ (right ordinate) that is estimated from the slope of ρ_{yx} in $|B| < 0.1$ T. **B-C** Magnetic field dependence of resistivity (ρ_{xx}) and Hall resistivity (ρ_{yx}) for $B\|c$ at various temperatures. Solid (dashed) lines are for a field increasing (decreasing) process. **D-E** Magnetic field dependence of (**D**) the conductivity (σ_{xx}) and (**E**) the Hall conductivity (σ_{xy}) for $B\|c$ at various temperatures. Data are shifted for clarity; the gray bar represents 10^5 S/cm; solid lines are two-band model fitting (See Sec. S6). **F-G** Obtained fitting parameters: (**F**) electron(hole)-type carrier density, n_e (n_h); (**G**) electron (hole) mobility, μ_e and (μ_h). **H** Temperature dependence of peak of MR for Fig. 3B, which effectively corresponds to the mass over carrier density.

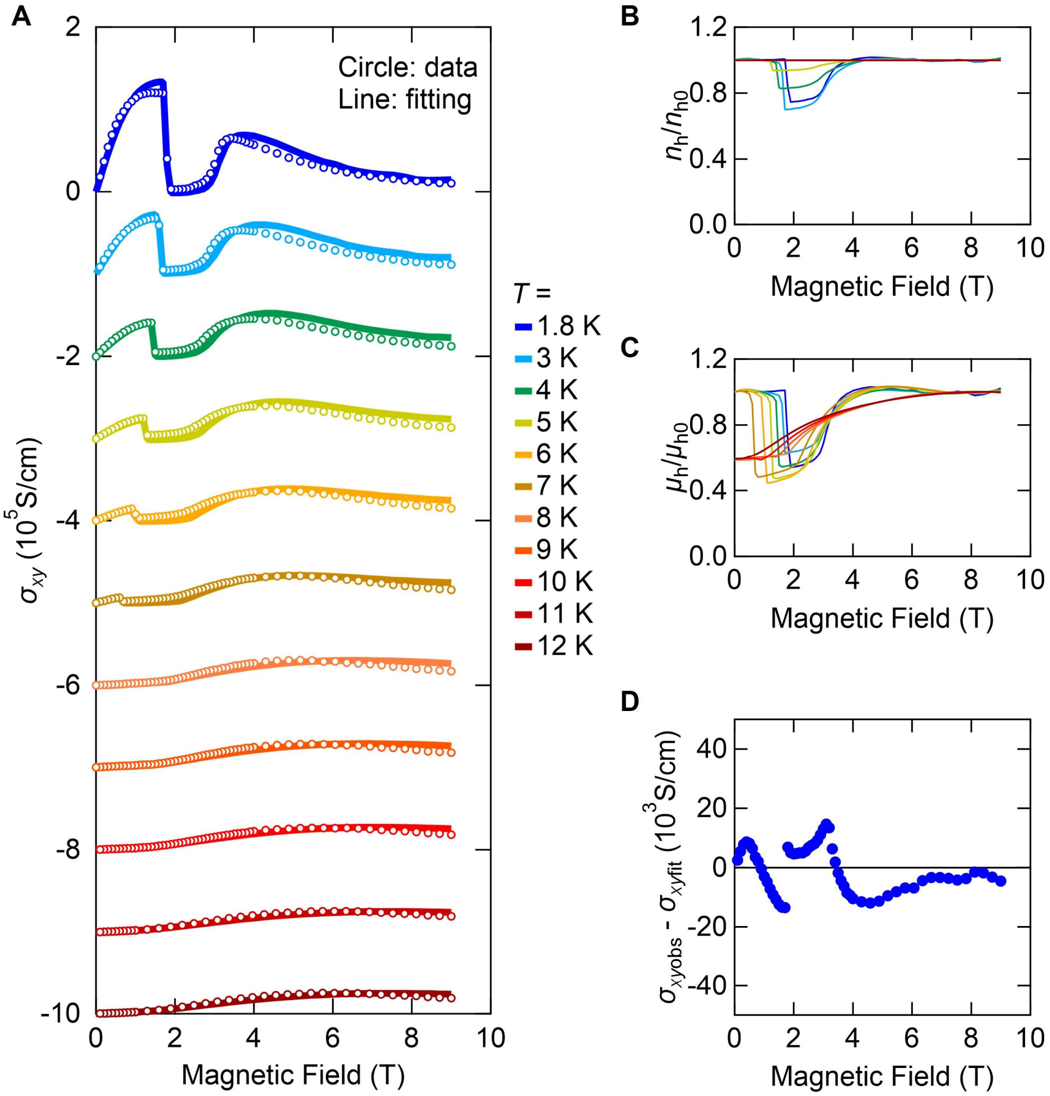

Fig. S19|Semiclassical two-band model analysis of σ_{xy}.

A Comparison between experimental data (circle) and fitting (solid line) at each temperature. **B-C** The hypothetical field-dependence of n_h/n_{h0} and μ_h/μ_{h0}, where n_{h0} and μ_{h0} is the carrier density of holes and mobility at high field. The field dependence of MR (Fig. 3) is used as a scale. (d) Difference between the observed (σ_{xyobs}) and fitted (σ_{xyfit}) Hall conductivity at 1.8 K providing estimate of uncertainty in modeling.

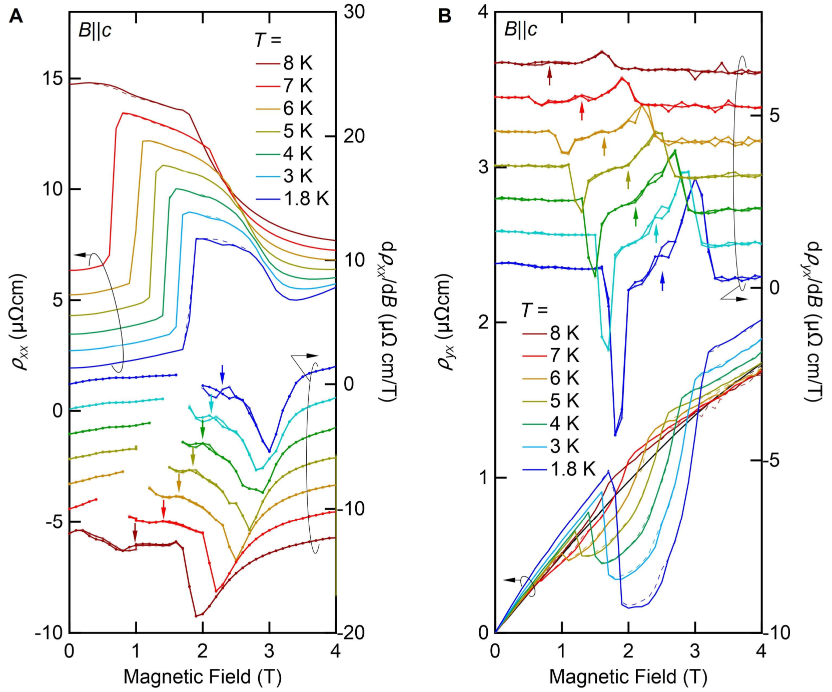

Fig. S20|Field-derivative of magnetotransport properties of $EuAg_4Sb_2$.

A Field dependence of the in-plane resistivity (ρ_{xx}, left ordinate) and field-derivative ($d\rho_{xx}/dB$, right ordinate). Solid (dashed) and closed (open) markers are for field-increasing and decreasing processes. Data are vertically shifted and sharp peak in $d\rho_{xx}/dB$ associated with the transition between ICM1 and ICM2 is removed for clarity. **B** Corresponding data for Hall resistivity (ρ_{yx}).

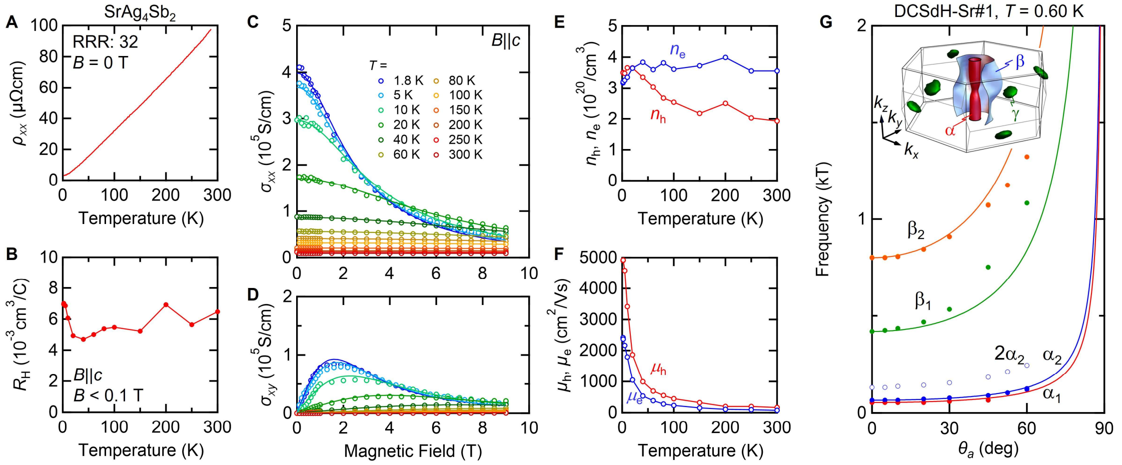

Fig. S21|Magnetotransport properties and two-band fitting for SrAg$_4$Sb$_2$.

A-B Temperature dependence of (**A**) the in-plane resistivity (ρ_{xx}) in zero field, and (**B**) the Hall coefficient (R_H) estimated from the slope of ρ_{yx} in $|B| < 0.1$ T for $B||c$. **C-D** Magnetic field dependence of (**C**) the conductivity (σ_{xx}) and (**D**) the Hall conductivity (σ_{xy}) for $B||c$ at various temperatures. Solid lines are for two-band model Fitting. **E-F** Obtained fitting parameters: (**E**) electron(hole)-type carrier density, n_e (n_h); (**F**) electron (hole) mobility, μ_e and (μ_h). **G** Angular (θ_a) dependence of the SdH oscillation frequency for each branch (closed circles). Open circles are for the second harmonics of α_2. Solid lines are fits with the model of a cylindrical Fermi surface. The inset is the Fermi surfaces of SrAg$_4$Sb$_2$ obtained by the DFT calculations. Red, blue, and green sheets are for the α (hole), β (hole) and γ (electron) pockets, respectively. Half of the β pocket is omitted for the visibility of the α pocket.

Table S2| Fermi surface parameters of $SrAg_4Sb_2$ estimated from SdH measurements and DFT Calculations.

m_{eff}: effective mass; m_0: free electron mass; f: oscillation frequency; k_F: Fermi wave number; v_F: Fermi velocity. Each parameter is estimated by the same methods for those of $EuAg_4Sb_2$.

DCSdH-Sr#1	α_1	α_2	β_1	β_2
Temperature dependence of FFT				
m_{eff}/m_0	0.401	0.054	0.280	0.263
f (T)	53.45	65.33	424.5	800.1
k_F (Å^{-1})	0.040	0.045	0.114	0.156
v_F (m/s)	1.2x10^5	9.5x10^5	4.7x10^5	6.9x10^5

DFT	α_1	α_2	β_1	β_2
k_F (Å^{-1})	0.040	0.070	0.211	0.261
v_F (m/s)	3.93x10^5	8.03x10^5	5.24x10^5	4.74x10^5

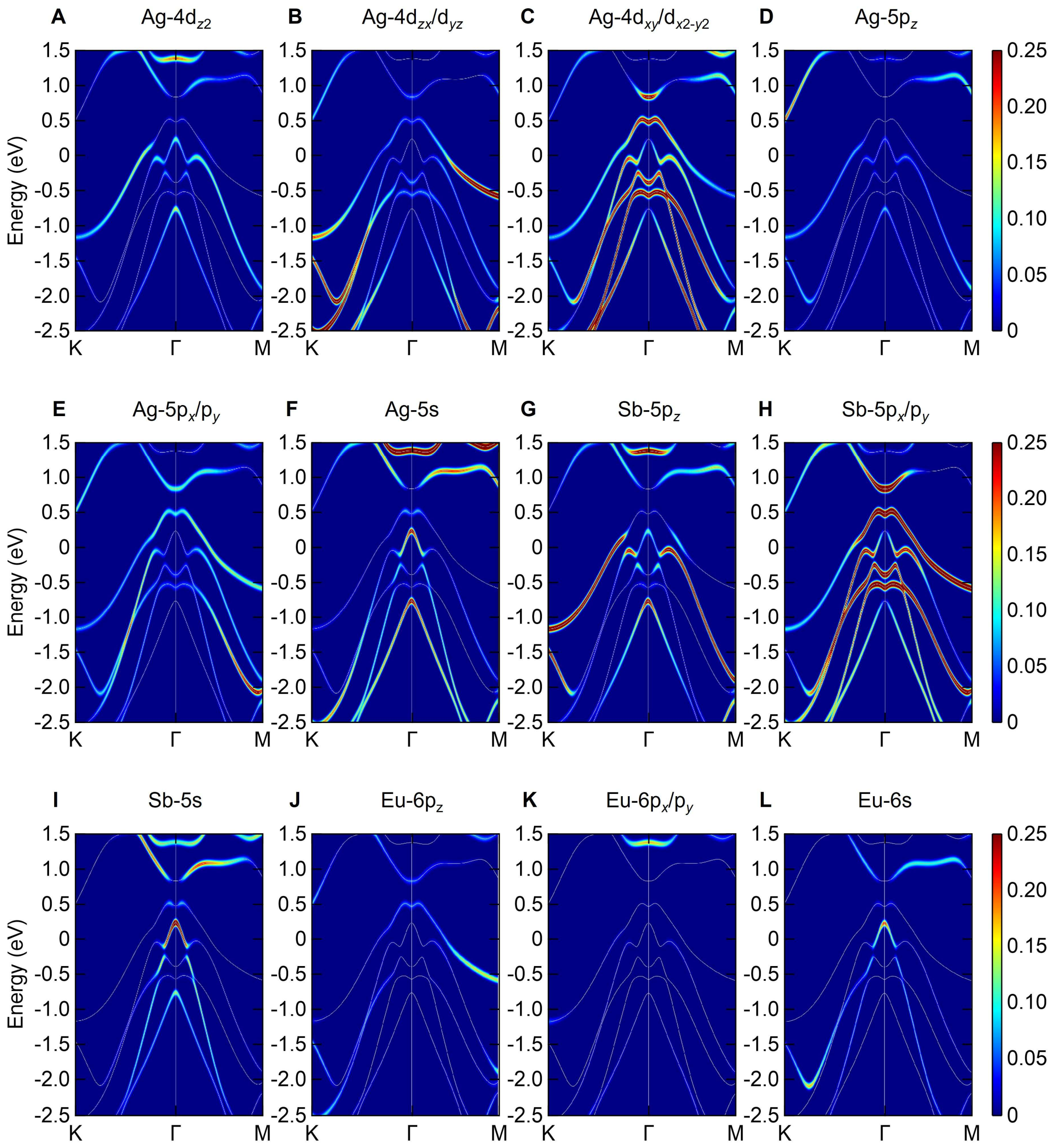

Fig. S22|Orbital character of each band in the K-Γ-M cut for $EuAg_4Sb_2$.

The color map of the weight of each orbital in the K-Γ-M cut of the band structure in $EuAg_4Sb_2$. **A** Ag-4d$_{z2}$, **B** Ag-4d$_{zx}$/d$_{yz}$, **C** Ag-4d$_{xy}$/d$_{x2\text{-}y2}$, **D** Ag-5p$_{z}$, **E** Ag-5p$_{x}$/p$_{y}$, **F** Ag-5s, **G** Sb-5p$_{z}$, **H** Sb-5p$_{x}$/p$_{y}$, **I** Sb-5s, **J** Eu-6p$_{z}$, **K** Eu-6p$_{x}$/p$_{y}$, and **L** Eu-6s. The Eu 4f bands are treated as a core electron shell in the pseudopotential and are not shown (see the details in Sec. S8).

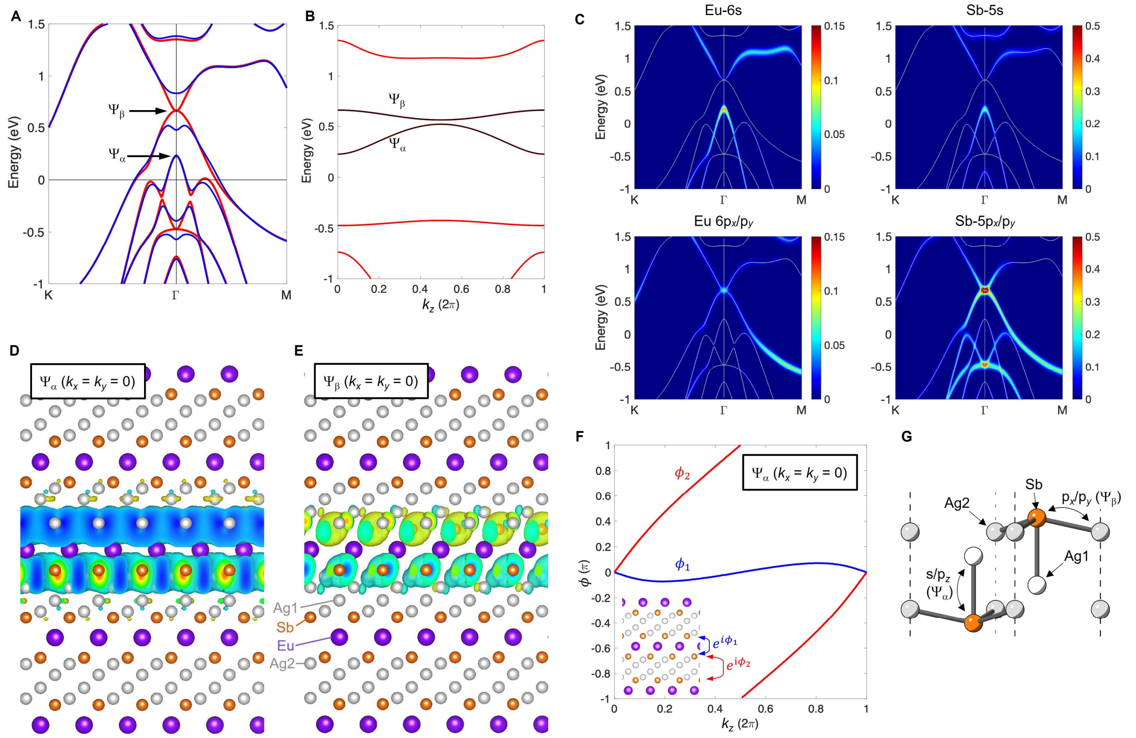

Fig. S23| The projections of Wannier functions and chemical bonds for electron pockets

A The electronic band structure for a non-magnetic ground state without (with) spin orbit coupling in red (blue), and the Ψ_α and Ψ_β states for Wannier transformation. **B** The k_z dispersions for the Ψ_α, Ψ_β states along Γ-A line, which are isolated from other bands and enable the definition for the proper projectors. **C** Band projections for Eu/Sb orbital states. Eu 6s/p_x/p_y are used as Wannier seeding functions for Ψ_α and Ψ_β states. **D-E** The converged Wannier functions at $k_x = k_y = 0$ in the real space for (**D**) Ψ_α and (**E**) Ψ_β. The latter is initialized with Eu p_x orbital. **F** The k_z dependence of the relative phase (ϕ) between wave functions at Sb s orbitals in neighboring Ag_2Sb layers. ϕ_1 (ϕ_2) is for that involving (not involving) the Eu layer (see the Inset). **G** Local bonding among Sb (orange) and Ag1 (white) and Ag2 (gray) atoms in the Ag_4Sb_2 bilayer.

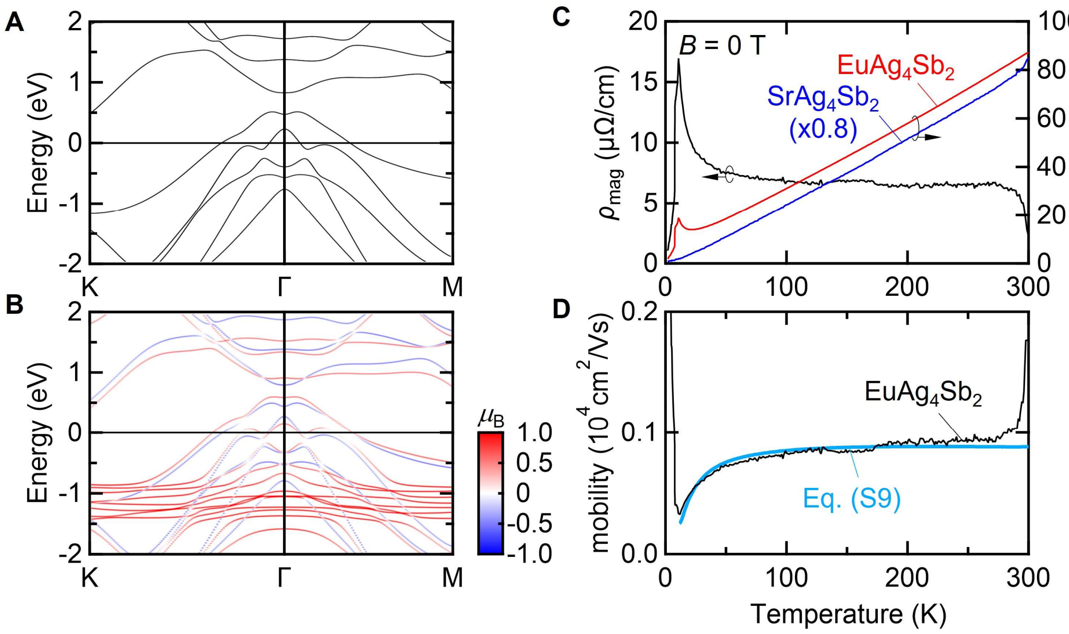

Fig. S24| Magnetic exchange coupling in the band structure

A-B The DFT band structures with spin-orbit coupling for a non-magnetic ground state (**A**) compared to the magnetic ground state (**B**) with the Eu magnetic moment align in the +*c* axis. The bands with the color scale indicating magnetic-moment projections parallel to the +*c* axis. The spin splitting energy at Γ is approximately 120 meV for the α pocket. **C** Temperature dependence of magnetic resistivity (ρ_{mag}) obtained by subtracting nonmagnetic resistivity in $EuAg_4Sb_2$ by using the resistivity of $SrAg_4Sb_2$ (right), which is scaled by 0.8. **D** Temperature dependence of the experimental magnetic part of Hall mobility (black) and the simulation (cyan) obtained by substituting $J = 90$ meV in Eq. (S11).

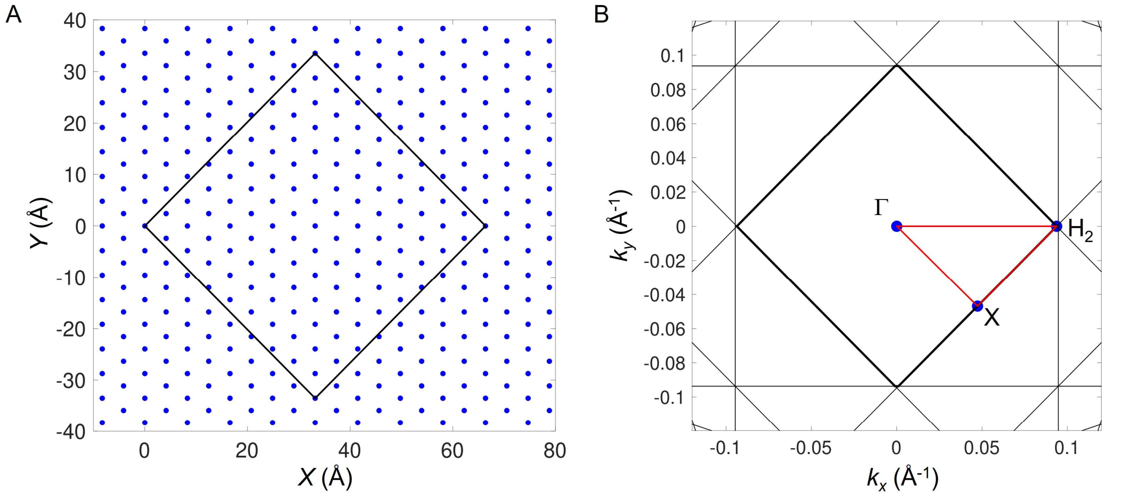

Fig. S25|Magnetic unit cell and magnetic BZ for the double-*q* spin structure.

A Real space magnetic super unit cell (black rhombus) for the double-*q* state on the triangular lattice of Eu atoms (blue dots). **B** Magnetic BZ (thick black lines) in the reciprocal space.

Table S3|Estimations for mean free path in various multi-*q* hosts.

Summary of the physical parameters of various multi-*q*-hosting materials taken (or estimated) from each reference. l_{mfp}: mean free path; a_{spin}: magnetic superlattice constant. "tr" stands for the transport mean free path, and "q" for the quantum lifetime measurements.

Noncentrosymmetric magnets											
Materials	MnSi	$Fe_{1-x}Co_xSi$	FeGe	FeGe	FeGe	FeGe	MnGe	EuPtSi	$Co_xZn_yMn_z$	$Mn_{1.5}Pd_{1-x}Pt_xSn$	$Fe_{1.5}Ni_{1-x}Pd_xP$
form	bulk	bulk	bulk	thin film 82 nm	thin film 18 nm, 300 nm	FIB	bulk	bulk	bulk	bulk/thin film 104 nm	bulk
method	dHvA	Hall	Hall	Hall	Hall	Hall	Hall	Hall	N/A	Hall	N/A
l_{mfp} (nm)	6 (28 K)	5-25	40	3	0.3<br>1.4	9.4	18	51	N/A	0.6	N/A
a_{spin} (nm)	22	50-266	81	81	81	81	3.2	2.0	132-216	156	231
R_H (cm^3/C)	-	-	-	2.6×10^{-4}	7.2×10^{-4}	7.3×10^{-3}	-	3.0×10^{-4}	N/A	1.7×10^{-4}	N/A
ρ ($\mu\Omega$cm)	-	-	-	50	1140<br>210	150	-	3.3	N/A	170	N/A
Ref.	27,28	29,30	31,32	33	35	34	31	23	36	37,38	39
**Centrosymmetric magnets**											
Materials	$EuAg_4Sb_2$	Gd_2PdSi_3	$Gd_3Ru_4Al_{12}$	$GdRu_2Si_2$	MnNiGa	$GdNi_2B_2C$	$EuAl_4$	$CeAuSb_2$	$SrFeO_3$		
Form	Bulk	bulk	bulk	bulk	bulk	Bulk	Bulk	Bulk	Bulk		
Method	Hall (tr)<br>SdH (q)	Hall	Hall	Hall	Hall	Hall	Hall	Hall	Hall		
l_{mfp} (nm)	253 (tr)<br>48 (q)	4.1	11.3	407	1.6	6.3	511	22	.9		
a_{spin} (nm)	4.6	2.9	3.2	1.9	208	7.1*	3.5	2.3	1.8		
R_H (cm^3/C)	-	6×10^{-4}	2×10^{-3}	4×10^{-4}	1.8×10^{-4}	1.2×10^{-4}	8.3×10^{-4}	33×10^{-4}	3.6×10^{-4}		
ρ ($\mu\Omega$cm)	-	65	52.5	0.5	75	15	0.65	37.5	200		
Ref.	This work	17	18	19	40	41,42	20	24,43	44		

REFERENCES AND NOTES

1. Y. Cao, V. Fatemi, S. Fang, K. Watanabe, T. Taniguchi, E. Kaxiras, P. Jarillo-Herrero, Unconventional superconductivity in magic-angle graphene superlattices. *Nature* **556**, 43–50 (2018).

2. M. Serlin, C. L. Tschirhart, H. Polshyn, Y. Zhang, J. Zhu, K. Watanabe, T. Taniguchi, L. Balents, A. F. Young, Intrinsic quantized anomalous Hall effect in a moiré heterostructure. *Science* **367**, 900–903 (2020).

3. K. Hamamoto, M. Ezawa, N. Nagaosa, Quantized topological Hall effect in skyrmion crystal. *Phys. Rev. B* **92**, 115417 (2015).

4. B. Göbel, A. Mook, J. Henk, I. Mertig, Unconventional topological Hall effect in skyrmion crystals caused by the topology of the lattice. *Phys. Rev. B* **95**, 094413 (2017).

5. Z. Wang, Y. Su, S.-Z. Lin, C. D. Batista, Skyrmion crystal from RKKY interaction mediated by 2D electron gas. *Phys. Rev. Lett.* **124**, 207201 (2020).

6. K. Shimizu, S. Okumura, Y. Kato, Y. Motome, Spin moiré engineering of topolgocial magnetism and emergent electromagnetic fields. *Phys. Rev. B* **103**, 184421 (2021).

7. I. Martin, C. D. Batista, Itinerant electron-driven chiral magnetic ordering and spontaneous quantum Hall effect in triangular lattice models. *Phys. Rev. Lett.* **101**, 156402 (2008).

8. K. Shimizu, S. Okumura, Y. Kato, Y. Motome, Phase degree of freedom and topology in multiple-Q spin texture. *Phys. Rev. B* **105**, 224405 (2022).

9. Y. Tokura, N. Kanazawa, Magnetic skyrmion materials. *Chem. Rev.* **121**, 2857–2897 (2021).

10. X. Gui, I. Pletikosic, H. Cao, H. J. Tien, X. Xu, R. Zhong, G. Wang, T. R. Chang, S. Jia, T. Valla, W. Xie, R. J. Cava, A new magnetic topological quantum material candidate by design. *ACS Cent. Sci.* **5**, 900–910 (2019).

11. S. Lei, J. Lin, Y. Jia, M. Gray, A. Topp, G. Farahi, S. Klemenz, T. Gao, F. Rodolakis, J. L. McChesney, C. R. Ast, A. Yazdani, K. S. Burch, S. Wu, N. P. Ong, L. M. Schoop, High mobility in a van der Waals layered antiferromagnetic metal. *Sci. Adv.* **6**, eaay6407 (2020).

12. H. Masuda, H. Sakai, M. Tokunaga, Y. Yamasaki, A. Miyake, J. Shiogai, S. Nakamura, S. Awaji, A. Tsukazaki, H. Nakao, Y. Murakami, T. H. Arima, Y. Tokura, S. Ishiwata, Quantum Hall effect in a bulk antiferromagnetic $EuMnBi_2$ with magnetically confined two-dimensional Dirac fermions. *Sci. Adv.* **2**, e1501117 (2016).

13. S. S. Stoyko, M. Khatun, C. S. Mullen, A. Mar, Ternary $CaCu_4P_2$-type pnictides $A$$Ag_4$$Pn_2$ (A = Sr, Eu; Pn = As, Sb). *J. Solid State Chem.* **192**, 325–330 (2012).

14. B. Gerke, C. Schwickert, S. S. Stoyko, M. Khatun, A. Mar, R. Pöttgen, Magnetic hyperfine field splitting in $EuAg_4As_2$ and $EuAg_4Sb_2$. *Solid State Sci.* **20**, 65–69 (2013).

15. S. Malick, H. Świątek, J. Bławat, J. Singleton, and T. Klimczuk, Large magnetoresistance and first-order phase transition in antiferromagnetic single-crystalline $EuAg_4Sb_2$. *Phys. Rev. B* **110**, 165149 (2024).

16. B. Shen, C. Hu, H. Cao, X. Gui, E. Emmanouilidou, W. Xie, N. Ni, Structural distortion and incommensurate noncollinear magnetism in $EuAg_4As_2$. *Phys. Rev. Mater.* **4**, 064419 (2020).

17. T. Kurumaji, T. Nakajima, M. Hirschberger, A. Kikkawa, Y. Yamasaki, H. Sagayama, H. Nakao, Y. Taguchi, T. H. Arima, Y. Tokura, Skyrmion lattice with a giant topological Hall effect in a frustrated triangular-lattice magnet. *Science* **365**, 914–918 (2019).

18. M. Hirschberger, T. Nakajima, S. Gao, L. Peng, A. Kikkawa, T. Kurumaji, M. Kriener, Y. Yamasaki, H. Sagayama, H. Nakao, K. Ohishi, K. Kakurai, Y. Taguchi, X. Yu, T. H. Arima, Y. Tokura, Skyrmion phases and competing magnetic orders on a breathing kagome lattice. *Nat. Commun.* **10**, 5831 (2019).

19. N. D. Khanh, T. Nakajima, X. Yu, S. Gao, K. Shibata, M. Hirschberger, Y. Yamasaki, H. Sagayama, H. Nakao, L. Peng, K. Nakajima, R. Takagi, T. H. Arima, Y. Tokura, S. Seki,

Nanometric square skyrmion lattice in a centrosymmetric tetragonal magnet. *Nat. Nanotech.* **15**, 444–449 (2020).

20. R. Takagi, N. Matsuyama, V. Ukleev, L. Yu, J. S. White, S. Francoual, J. R. L. Mardegan, S. Hayami, H. Saito, K. Kaneko, K. Ohishi, Y. Ōnuki, T.-H. Arima, Y. Tokura, T. Nakajima, S. Seki, Square and rhombic lattices of magnetic skyrmions in a centrosymmetric binary compound. *Nat. Commun.* **13**, 1472 (2022).

21. D. Singh, Y. Fujishiro, S. Hayami, S. H. Moody, T. Nomoto, P. R. Baral, V. Ukleev, R. Cubitt, N. J. Steinke, D. J. Gawryluk, E. Pomjakushina, Y. Ōnuki, R. Arita, Y. Tokura, N. Kanazawa, J. S. White, Transition between distinct hybrid skyrmion textures through their hexagonal-to-square crystal transformation in a polar magnet. *Nat. Commun.* **14**, 8050 (2023).

22. J. Green, E. Emmanouilidou, H. W. Morgan, W. T. Laderer, C. Hu, J. Loera, A. N. Alexandrova, and N. Ni. Fermiology and transport properties of the candidate topological crystalline insulator $SrAg_4Sb_2$. *Phys. Rev. Mater.* **8**, 054205, (2024).

23. M. Kakihana, D. Aoki, A. Nakamura, F. Honda, M. Nakashima, Y. Amako, S. Nakamura, T. Sakakibara, M. Hedo, T. Nakama, Y. Ōnuki, Giant Hall resistivity and magnetoresistance in cubic chiral antiferromagnet EuPtSi. *J. Phys. Soc. Jpn.* **87**, 023701 (2018).

24. G. G. Marcus, D.-J. Kim, J. A. Tutmaher, J. A. Rodriguez-Rivera, J. O. Birk, C. Niedermeyer, H. Lee, Z. Fisk, C. L. Broholm, Multi-q mesoscale magnetism in $CeAuSb_2$. *Phys. Rev. Lett.* **120**, 097201 (2018).

25. R. Ritz, M. Halder, C. Franz, A. Bauer, M. Wagner, R. Bamler, A. Rosch, C. Pfleiderer, Giant generic topological Hall resistivity of MnSi under pressure. *Phys. Rev. B* **87**, 134424 (2013).

26. K. Nakazawa, M. Bibes, H. Kohno, Topological Hall effect from strong to weak coupling. *J. Phys. Soc. Jpn.* **87**, 033705 (2018).

27. C. Pfleiderer, G. J. McMullan, S. R. Julian, G. G. Lonzarich, Magnetic quantum phase transition in MnSi under hydrostatic pressure. *Phys. Rev. B* **55**, 8330–8338 (1997).

28. L. Taillefer, G. G. Lonzarich, P. Strange, The band magnetism of MnSi. *J. Magn. Magn. Mater.* **54-57**, 957–958 (1986).

29. Y. Onose, N. Takeshita, C. Terakura, H. Takagi, Y. Tokura, Doping dependence of transport properties in $Fe_{1-x}Co_{x}Si$. *Phys. Rev. B* **72**, 224431 (2005).

30. J. Beille, J. Voiron, M. Roth, Long period helimagnetism in the cubic B20 $Fe_{x}Co_{1-x}Si$ and $Co_{x}Mn_{1-x}Si$ alloys. *Solid State Commun.* **47**, 399–402 (1983).

31. N. Kanazawa, Charge and Heat Transport Phenomena in Electronic and Spin Structures in B20-type Compounds (Springer, 2015).

32. B. Lebech, J. Bernhard, T. Freltoft, Magnetic structures of cubic FeGe studied by small-angle neutron scattering. *J. Phys. Condens. Matter* **1**, 6105–6122 (1989).

33. N. A. Porter, J. C. Gartside, C. H. Marrows, Scattering mechanisms in textured FeGe thin films: Magnetoresistance and the anomalous Hall effect. *Phys. Rev. B* **90**, 024403 (2014).

34. M. Leroux, M. J. Stolt, S. Jin, D. V. Pete, C. Reichhardt, B. Maiorov, Skyrmion lattice topological Hall effect near room temperature. *Sci. Rep.* **8**, 15510 (2018).

35. S. X. Huang, C. L. Chien, Extended skyrmion phase in epitaxial FeGe(111) thin films. *Phys. Rev. Lett.* **108**, 267201 (2012).

36. Y. Tokunaga, X. Z. Yu, J. S. White, H. M. Rønnow, D. Morikawa, Y. Taguchi, Y. Tokura, A new class of chiral materials hosting magnetic skyrmions beyond room temperature. *Nat. Commun.* **6**, 7638 (2015).

37. A. K. Nayak, V. Kumar, T. Ma, P. Werner, E. Pippel, R. Sahoo, F. Damay, U. K. Rößler, C. Felser, S. S. P. Parkin, Magnetic antiskyrmions above room temperature in tetragonal Heusler materials. *Nature* **548**, 561–566 (2017).

38. P. Swekis, A. Markou, D. Kriegner, J. Gayles, R. Schlitz, W. Schnelle, S. T. B. Goennenwein, C. Felser, Topological Hall effect in thin films of $Mn_{1.5}PtSn$. *Phys. Rev. Mater.* **3**, 013001(R) (2019).

39. K. Karube, L. Peng, J. Masell, X. Yu, F. Kagawa, Y. Tokura, Y. Taguchi, Room-temperature antiskyrmions and sawtooth surface textures in a non-centrosymmetric magnet with S_4 symmetry. *Nat. Mater.* **20**, 335–340 (2021).

40. W. Wang, Y. Zhang, G. Xu, L. Peng, B. Ding, Y. Wang, Z. Hou, X. Zhang, X. Li, E. Liu, S. Wang, J. Cai, F. Wang, J. Li, F. Hu, G. Wu, B. Shen, X.-X. Zhang, A centrosymmetric hexagonal magnet with superstable biskyrmion magnetic nanodomains in a wide temperature range of 100-340 K. *Adv. Mater.* **28**, 6887–6893 (2016).

41. P. Mandal, K. Winzer, The transport properties of magnetic and nonmagnetic borocarbides. *Solid State Commun.* **103**, 679–682 (1997).

42. P. S. Normile, M. Rotter, C. Detlefs, J. Jensen, P. C. Canfield, J. A. Blanco, Magnetic ordering in $GdNi_2B_2C$ revisited by resonant x-ray scattering: Evidence for the double-q model. *Phys. Rev. B* **88**, 054413 (2013).

43. K.-A. Lorenzer, A. M. Strydom, A. Thamizhavel, S. Paschen, Temperature-field phase diagram of quantum critical CeAuSb2. *Phys. Status Solidi B* **250**, 464–467 (2013).

44. S. Ishiwata, M. Tokunaga, Y. Kaneko, D. Okuyama, Y. Tokunaga, S. Wakimoto, K. Kakurai, T. Arima, Y. Taguchi, Y. Tokura, Versatile helimagnetic phases under magnetic fields in cubic perovskite $SrFeO_3$. *Phys. Rev. B* **84**, 054427 (2011).

45. N. Paul, Y. Zhang, L. Fu, Giant proximity exchange and flat Chern band in 2D magnet-semiconductor heterostructures. *Sci. Adv.* **9**, eabn1401 (2023).

46. D. Solenov, D. Mozyrsky, I. Martin, Chirality waves in two-dimensional magnets. *Phys. Rev. Lett.* **108**, 096403 (2012).

47. Z. Wang, C. D. Batista, Skyrmion crystals in the triangular Kondo lattice model. arXiv:2111.13976 [cond-mat.str-el] (2021).

48. M. Q. Arguilla, N. D. Cultrara, Z. J. Baum, S. Jiang, R. D. Ross, J. E. Goldberger, $EuSn_2As_2$: Exfoliatable magnetic layered Zintl-Klemm phase. *Inorg. Chem. Front.* **4**, 378–386 (2017).

49. P. M. Neves, J. S. White, GRASP integrated 3D plotter: GRIP. *J. Appl. Cryst.* **57**, 2030–2042 (2024).

50. J. W. Lynn, Y. Chen, S. Chang, Y. Zhao, S. Chi, W. Ratcliff II, B. G. Ueland, R. W. Erwin, Double-focusing thermal triple-axis spectrometer at the NCNR. *J. Res. Natl. Inst. Stand. Technol.* **117**, 61–79 (2012).

51. V. Brouet, W. L. Yang, X. J. Zhou, Z. Hussain, N. Ru, K. Y. Shin, I. R. Fisher, Z. X. Shen, Fermi surface reconstruction in the CDW state of $CeTe_3$ observed by photoemission. *Phys. Rev. Lett.* **93**, 126405 (2004).

52. J. Voit, L. Perfetti, F. Zwick, H. Berger, G. Margaritondo, G. Grüner, H. Höchst, M. Grioni, Electronic structure of solids with competing periodic potentials. *Science* **290**, 501–503 (2000).

53. H. Su, B. Gong, W. Shi, H. Yang, H. Wang, W. Xia, Z. Yu, P.-J. Guo, J. Wang, L. Ding, L. Xu, X. Li, X. Wang, Z. Zou, N. Yu, Z. Zhu, Y. Chen, Z. Liu, K. Liu, G. Li, Y. Guo, Magnetic exchange induced Weyl state in a semimetal $EuCd_2Sb_2$. *APL Mater.* **8**, 011109 (2020).

54. S. L. Bud'ko, L. Xiang, C. Hu, B. Shen, N. Ni, P. C. Canfield, Pressure tuning of structural and magnetic transitions in $EuAg_4As_2$. *Phys. Rev. B* **101**, 195112 (2020).

55. H. Takatsu, S. Yonezawa, C. Michioka, K. Yoshimura, Y. Maeno, Anisotropy in the magnetization and resistivity of the metallic triangular-lattice magnet $PdCrO_2$. *J. Phys. Conf. Ser.* **200**, 012198 (2010).

56. D. Schoenberg, *Magnetic Oscillations in Metals* (Cambridge Univ. Press, 1984).

57. D. H. Ryan, S. L. Bud'ko, C. Hu, N. Ni, Magnetic and structural transitions in $EuAg_4As_2$ studied using ^{151}Eu Mössbauer spectroscopy. *AIP Adv.* **9**, 125050 (2019).

58. V. Hardy, Y. Bréard, C. Martin, Derivation of the heat capacity anomaly at a first-order transition by using a semi-adiabatic relaxation technique. *J. Phys. Condens. Matter* **21**, 075403 (2009).

59. J. C. Lashley, M. F. Hundley, A. Migliori, J. L. Sarrao, P. G. Pagliuso, T. W. Darling, M. Jaime, J. C. Cooley, W. L. Hults, L. Morales, D. J. Thoma, J. L. Smith, J. Boerio-Goates, B. F. Woodfield, G. R. Stewart, R. A. Fisher, N. E. Phillips, Critical examination of heat capacity measurements made on a Quantum Design physical properties measurement system. *Cryogenics* **43**, 369–378 (2003).

60. Q. Zhu, L. Li, Z.-H. Yang, Z.-F. Lou, J.-H. Du, J.-H. Yang, B. Chen, H.-D. Wang, M.-H. Fang, Metamagnetic transitions and anomalous magnetoresistance in $EuAg_4As_2$ crystals. *Sci. China Phys. Mech. Astron.* **64**, 227011 (2021).

61. S. Gao, O. Zaharko, V. Tsurkan, Y. Su, J. S. White, G. S. Tucker, B. Roessli, F. Bourdarot, R. Sibille, D. Chernyshov, T. Fennell, A. Loidl, C. Rüegg, Spiral spin-liquid and the emergence of a vortex-like state in $MnSc_2S_4$. *Nat. Phys.* **13**, 157–161 (2017).

62. S. Bordács, A. Butykai, B. G. Szigeti, J. S. White, R. Cubitt, A. O. Leonov, S. Widmann, D. Ehlers, H.-A. K. von Nidda, V. Tsurkan, A. Loidl, I. Kézsmárki, Equilibrium skyrmion lattice ground state in a polar easy-plane magnet. *Sci. Rep.* **7**, 7584 (2017).

63. T. Adams, M. Garst, A. Bauer, R. Georgii, C. Pfleiderer, Response of the skyrmion lattice in MnSi to cubic magnetocrystalline anisotropy. *Phys. Rev. Lett.* **121**, 187205 (2018).

64. T. Okubo, S. Chung, H. Kawamura, Multiple-q states and the skyrmion lattice of the triangular-lattice Heisenberg antiferromagnet under magnetic fields. *Phys. Rev. Lett.* **108**, 017206 (2012).

65. Y. Fujima, N. Abe, Y. Tokunaga, T. Arima, Thermodynamically stable skyrmion lattice at low temperatures in a bulk crystal of lacunar spinel GaV_4Se_8. *Phys. Rev. B* **95**, 180410(R) (2017).

66. R. Ozawa, S. Hayami, Y. Motome, Zero-field skyrmions with a high topological number in itinerant magnets. *Phys. Rev. Lett.* **118**, 147205 (2017).

67. S. Hayami, Zero-field skyrmion, meron, and vortex crystals in centrosymmetric hexagonal magnets. *J. Magn. Magn. Mater.* **564**, 170036 (2022).

68. K. Kobayashi, S. Hayami, Skyrmion and vortex crystals in the Hubbard model. *Phys. Rev. B* **106**, L140406 (2022).

69. S. Hayami, Skyrmion crystals in centrosymmetric triangular magnets under hexagonal and trigonal single-ion anisotropy. *J. Magn. Magn. Mater.* **553**, 169220 (2022).

70. S. Hayami, Y. Motome, Noncoplanar multiple-Q spin textures by itinerant frustration: Effects of single-ion anisotropy and bond-dependent anisotropy. *Phys. Rev. B* **103**, 054422 (2021).

71. O. I. Utesov, Thermodynamically stable skyrmion lattice in a tetragonal frustrated antiferromagnet with dipolar interaction. *Phys. Rev. B* **103**, 064414 (2021).

72. R. Ozawa, S. Hayami, K. Barros, G.-W. Chern, Y. Motome, C. D. Batista, Vortex cyrstals with chiral stripes in itinerant magnets. *J. Phys. Soc. Jpn.* **85**, 103703 (2016).

73. S. Hayami, Rectangular and square skyrmion crystals on a centrosymmetric square lattice with easy-axis anisotropy. *Phys. Rev. B* **105**, 174437 (2022).

74. B. Shen, E. Emmanouilidou, X. Deng, A. McCollam, J. Xiang, G. Kotliar, A. I. Coldea, N. Ni, Significant change in the electronic behavior associated with structural distortions in monocrystalline $SrAg_4As_2$. *Phys. Rev. B* **98**, 235130 (2018).

75. G. Kresse, J. Furthmüller, Efficient iterative schemes for ab initio total-energy calculations using a plane-wave basis set. *Phys. Rev. B* **54**, 11169–11186 (1996).

76. G. Kresse, J. Furthmüller, Efficiency of ab-initio total energy calculations for metals and semiconductors using a plane-wave basis set. *Comput. Mater. Sci.* **6**, 15–50 (1996).

77. P. E. Blöchl, Projector augmented-wave method. *Phys. Rev. B* **50**, 17953–17979 (1994).

78. S. L. Dudarev, G. A. Botton, S. Y. Savrasov, C. J. Humphreys, A. P. Sutton, Electron-energy-loss spectra and the structural stability of nickel oxide: An LSDA+U study. *Phys. Rev. B* **57**, 1505–1509 (1998).

79. J. P. Perdew, K. Burke, M. Ernzerhof, Generalized gradient approximation made simple. *Phys. Rev. Lett.* **77**, 3865–3868 (1996).

80. H. J. Monkhorst, J. D. Pack, Special points for Brillouin-zone integrations. *Phys. Rev. B* **13**, 5188–5192 (1976).

81. N. Marzari, A. A. Mostofi, J. Yates, R. Yates, I. Souza, D. Vanderbilt, Maximally localized Wannier functions: Theory and applications. *Rev. Mod. Phys.* **84**, 1419–1475 (2012).

82. A. A. Mostofi, J. R. Yates, G. Pizzi, Y.-S. Lee, I. Souza, D. Vanderbilt, N. Marzari, An updated version of wannier90: A tool for obtaining maximally-localised Wannier functions. *Comput. Phys. Commun.* **185**, 2309–2310 (2014).

83. C. Tang, Z. Zhang, S. Lai, Q. Tan, W.-B. Gao, Magnetic proximity effect in graphene/$CrBr_3$ van der Waals heterostructures. *Adv. Mater.* **32**, e1908498 (2020).

84. J.-R. Soh, C. Donnerer, K. M. Hughes, E. Schierle, E. Weschke, D. Prabhakaran, A. T. Boothroyd, Magnetic and electronic structure of the layered rare-earth pnictide $EuCd_2Sb_2$. *Phys. Rev. B* **98**, 064419 (2018).

85. S. Fang, L. Ye, M. P. Ghimire, M. Kang, J. Liu, M. Han, L. Fu, M. Richer, J. van den Brink, E. Kaxiras, R. Comin, J. G. Checkelsky, Ferromagnetic helical line and Kane-Mele spin-orbit coupling in kagome metal Fe_3Sn_2. *Phys. Rev. B* **105**, 035107 (2022).

86. C. Haas, Spin-disorder scattering and magnetoresistance of magnetic semiconductors. *Phys. Rev.* **168**, 531–538 (1968).

87. A. W. Tyler, A. P. Mackenzie, S. NishiZaki, Y. Maeno, High-temperature resistivity of Sr_2RuO_4: Bad metallic transport in a good metal. *Phys. Rev. B* **58**, R10107(R) (1998).

88. M. Hirschberger, L. Spitz, T. Nomoto, T. Kurumaji, S. Gao, J. Masell, T. Nakajima, A. Kikkawa, Y. Yamasaki, H. Sagayama, H. Nakao, Y. Taguchi, R. Arita, T.-h. Arima, Y. Tokura, Topological Nernst effect of the two-dimensional skyrmion lattice. *Phys. Rev. Lett.* **125**, 076602 (2020).

89. T. Nomoto, T. Koretsune, R. Arita, Formation mechamism of the helical Q structure in Gd-based skyrmion materials. *Phys. Rev. Lett.* **125**, 117204 (2020).

90. B. Juba, E. Mendive-Tapia, S. Blügel, J. B. Staunton, Fermi-surface origin of skyrmion lattices in centrosymmetric rare-earth intermetallics. *Phys. Rev. Lett.* **128**, 157206 (2022).

91. S. Paul, S. Haldar, S. von Malottki, S. Heinze, Role of higher-order exchange interactions for skyrmion stability. *Nat. Commun.* **11**, 4756 (2020).

92. M. Azhar, M. Mostovoy, Incommensurate spiral order from double-exchange interactions. *Phys. Rev. Lett.* **118**, 027203 (2017).
